# Expression and Functional Analysis of Immuno-Micro-RNAs mir-146a and mir-326 in Colorectal Cancer

**DOI:** 10.3390/cimb46070421

**Published:** 2024-07-05

**Authors:** Ovidiu Farc, Liviuta Budisan, Florin Zaharie, Roman Țăulean, Dan Vălean, Elena Talvan, Ioana Berindan Neagoe, Oana Zănoagă, Cornelia Braicu, Victor Cristea

**Affiliations:** 1 Research Center for Functional Genomics, Biomedicine and Translational Medicine “Iuliu Hatieganu” University of Medicine and Pharmacy, 400337 Cluj-Napoca, Romania; farc.ovidiu@yahoo.com (O.F.); ioana.neagoe@umfcluj.ro (I.B.N.); oana.zanoaga@umfcluj.ro (O.Z.); braicucornelia@yahoo.com (C.B.); 2 Surgical Department, “Iuliu Hatieganu” University of Medicine and Pharmacy, 400347 Cluj-Napoca, Romania; florinzaharie@yahoo.com (F.Z.); axiromplus@gmail.com (R.Ț.); valean.d92@gmail.com (D.V.); 3 Faculty of Medicine Lucian Blaga, University of Sibiu, 550169 Sibiu, Romania; elena.talvan@ulbsibiu.ro; 4 Immunology Department, “Iuliu Hatieganu” University of Medicine and Pharmacy, 400347 Cluj-Napoca, Romania; victor.cristea@umfcluj.ro

**Keywords:** colorectal, cancer, micro-RNA, network, modules

## Abstract

Micro-RNAs (miRNAs) are non-coding RNAs with importance in the development of cancer. They are involved in both tumor development and immune processes in tumors. The present study aims to characterize the behavior of two miRNAs, the proinflammatory miR-326-5p and the anti-inflammatory miR-146a-5p, in colorectal cancer (CRC), to decipher the mechanisms that regulate their expression, and to study potential applications. Tissue levels of miR-326-5p and miR-146a-5p were determined by qrt-PCR (real-time quantitative reverse transcription polymerase chain reaction) in 45 patients with colorectal cancer in tumoral and normal adjacent tissue. Subsequent bioinformatic analysis was performed to characterize the transcriptional networks that control the expression of the two miRNAs. The biomarker potential of miRNAs was assessed. The expression of miR-325-5p and miR-146a-5p was decreased in tumors compared to normal tissue. The two miRNAs are regulated through a transcriptional network, which originates in the inflammatory and proliferative pathways and regulates a set of cellular functions related to immunity, proliferation, and differentiation. The miRNAs coordinate distinct modules in the network. There is good biomarker potential of miR-326 with an AUC (Area under the curve) of 0.827, 0.911 sensitivity (Sn), and 0.689 specificity (Sp), and of the combination miR-326-miR-146a, with an AUC of 0.845, Sn of 0.75, and Sp of 0.89. The miRNAs are downregulated in the tumor tissue. They are regulated by a transcriptional network in which they coordinate distinct modules. The structure of the network highlights possible therapeutic approaches. MiR-326 and the combination of the two miRNAs may serve as biomarkers in CRC.

## 1. Introduction

Micro-RNAs (miRNAs) are short, single-stranded RNA molecules that regulate gene expression by inhibiting RNA messages based on partial complementarity with target RNAs [1]. They are present in a wide range of biological processes, such as the control of metabolism, proliferation, differentiation, or embryo development, and in virtually all cellular processes involving DNA transcription. Their dysregulation is known to contribute to the development of various forms of cancer. Consequently, a lot of research work has been dedicated to the study of their mechanisms and diagnostic or prognostic implications in tumors [2]. There have been fewer studies that have focused on micro-RNAs with a role in tumor immunity, an area with growing importance and increasing role in cancer therapy [3]. The present study aims to address this field by considering two miRNAs with importance in the immune system, the proinflammatory miR-326-5p and the anti-inflammatory miR-146a-5p, and by studying their behavior in colorectal cancer (CRC).

MiR-146a is an intronic miRNA located on chromosome 5q33.3, on the gene of a long non-coding RNA (MIR3124 HG) [4]. Its synthesis is controlled by several transcription factors (TFs), among which are *NfkB*, *CEBPα* (CCAAT-enhancer binding protein α), and *TGFβ* [4]. Its expression increases after stimulation of TLRs (Toll-like receptors) 2,4, and 7, and it negatively regulates the immune pathways, targeting IL (interleukin)-6, IL-8, CCL (CC-chemokine-ligand) 2, and IRAK (IL-receptor-associated kinase)-1 [5]. Its action in macrophages favors the M2 anti-inflammatory profile and stimulates IL-10 [5]. Concerning the adaptive response, miR-146a stimulates Tregs (T regulatory lymphocytes) [5], the Th2 (T helper-2) profile in CD4+ LTs (lymphocytes) and inhibits the Th17 profile. It also increases under stimulation via the RAS-ETS1 pathway, resulting in the inhibition of cell cycle and proliferation [5]. 

In cancer, miR-146a has been shown to play a complex role, both pro- and antitumoral. In pancreatic cancer, its role is mainly antitumoral by targeting the EGFR and NfkB signaling, with a known protumoral role in these tumors. It decreases in tumor tissue. Its reexpression leads to the inhibition of tumor growth and invasiveness [6]. In esophageal cancer, it is downregulated in serum. Its decrease is associated with a worse prognosis [7]. In hormone-refractory prostatic tumors, there is a decreased level of miR-146a, and transfection of this miRNA markedly reduces the proliferation and invasiveness of tumor cells, mainly through the inhibition of the HA (hyaluronan)-ROCK-1 axis, a mechanism with a known role in the pathogenesis of this cancer [8]. MiR-146a levels are also decreased in ovarian cancer, where overexpression of this miRNA leads to reduced proliferation and increased apoptosis. In these tumors, the target of miR-1456a has been shown to be SOD (superoxide dismutase)-2, whose inhibition leads to an increase in ROS (reactive oxygen species) and consequent apoptosis [9]. Other tumors where miR-146a has been shown to act as a tumor-suppressor are lung [10] and gastric cancer [11]. Conversely, in melanoma, miR-146a has an oncogenic role and an increased level. Its overexpression leads to an accelerated proliferation. Its target in these tumors is NUMB, a Notch inhibitor, which stops progenitor proliferation and induces differentiation. [12]. In cervical cancer, miR-146a also has an increased level and a tumor-promoting role, which it exerts by inhibiting IRAK1 and TRAF-6 (TNF-receptor-associated factor 6) and stimulating Cyclin-D1 [13]. It has been studied as a biomarker in cancer [7]. Its role in colorectal cancer is complex, mostly antitumoral [14], through the inhibition of the cell cycle and of the IL-17-cell effector axis [15]. There are studies that report a prometastatic or immunosuppressive role [16,17]. Hwang et al. showed a protumoral effect of this miRNA in CRC, similar to that in melanoma, by inhibiting the NUMB-Notch axis and the differentiation of colon tumor cells [18]. miR-146a expression is increased in the colon, mostly in immunocytes (CD45+ cells), much less in the intestinal non-immune cells [19].

MiR-326 is also an intronic miRNA situated on the first intron of the β-arrestin-1 gene on chromosome 11q [20]. It plays a role in neuronal development, along with its host gene, by inhibiting the Notch pathway, which leads to neuronal differentiation. It also stimulates some other differentiations, such as adipocytic, osteoblastic, myogenic, or erythroid differentiation [21,22]. It also regulates the endocytic pathway and GPCR (G-protein coupled receptors) signaling [23,24].

In immunity, miR-326 is pro-inflammatory, and it stimulates the Th17 profile in CD4+ LTs [25]. In lung cancer, the downregulation of this miRNA has been associated with a worse prognosis. It acts by targeting Cyclin D1 and the metalloproteinase MMP-9, decreasing tumor proliferation and invasiveness [26]. In gastric cancer, it is a tumor suppressor, and its level is decreased in these tumors [27]. In breast cancer, there is also a low level of this tumor suppressor miRNA [28]. In prostatic tumors, its levels are decreased, and it exerts a tumor-suppressive role through targeting ELK1, a proliferative TF [29]. In conclusion, the evidence points toward a tumor-suppressive role of miR-326 in most tumors [30]. Its evaluation as a biomarker showed promising results [31]. In CRC, it is considered a tumor-suppressive miRNA through multiple mechanisms such as targeting of pro-metastatic (*NOB1*, *ADAM-17*, *ZEB1*) and proliferative (*CCND1*, *FGF)* elements [32]. In colorectal cancer, its expression was detected in the tumor cells, stroma, and immunocytes, as well as in cell lines [33].

## 2. Materials and Methods

This is an observational study in which 45 patients with colorectal adenocarcinoma with pathological confirmation were included. The patients were treated in the Third Surgical Clinic of the Regional Gastroenterology and Hepatology of Cluj-Napoca, Romania, and in the Surgical Clinic of the County Emergency Hospital of Sibiu, Romania, between November 2018 and March 2020. The exclusion criteria were patients who underwent therapeutic procedures that could alter tissue miRNA expression (chemotherapy, radiotherapy, others), patients with active infections or active neoplasms in other locations, patients with inflammatory bowel disease, and patients with severe organic deficiencies.

All subjects completed a standardized questionnaire on demographics (age, gender) and general health information, including comorbidities. Information on tumor stage, grade, location, and other clinical data and procedures were obtained from each patient’s hospital medical record. After obtaining the approvals of the Ethics Committees of the Iuliu Hatieganu Medicine and Pharmacy University from Cluj-Napoca, Romania (no. 40/02.04.2018), County Emergency Hospital of Sibiu, Romania (no. 10759/23 05.2019), and of the Regional Institute of Gastroenterology and Hepatology from Cluj-Napoca, Romania (no. 2769/1.03.2018), written informed consent was obtained from each patient.

Collection and storage of the samples. Colon and rectal tumor tissue, as well as normal adjacent tissue, were collected from patients by intraoperative section. Samples were stored at −170 °C in liquid nitrogen until testing.

miRNA extraction and quality control. Total RNA from the normal and tumor tissue was extracted using TriReagent (Thermo Fischer Scientific, Waltham, MA, USA), according to the manufacturer’s indications. RNA quality and concentration were evaluated using NanoDrop-1100. The purity of samples was evaluated based on the spectral data and purity ratios.

miRNAs and housekeeping gene sequences (according to miRbase):

hsa-miR-146a-UGAGAACUGAAUUCCAUGGGUU

hsa-miR-326-CCUCUGGGCCCUUCCUCCAG

RNU6B-CGCAAGGATGACACGCAAATTCGTGAAGCGTTCCATATTTTT

RNU48-GATGACCCCAGGTAACTCTGAGTGTGTCGCTGATGCCATCAC-CGCAGCGCTCTGACC

q*RT-PCR* (real-time quantitative reverse transcription polymerase chain reaction) analysis. qRT-PCR miRNA primer assays were obtained from Thermo Fischer Scientific (Waltham, MA, USA). For miR-146a, we used kit no. 002163 and for miR-326, kit no. 000518 was used. Assays no. 001006 for RNU48 and 001093 for RNU6B were used as housekeeping miRNAs.

A total of 50 ng RNA for miRNAs (hsa-miR-146a and hsa-miR-326) expression was transcribed into cDNA using a TaqMan MicroRNA Reverse Transcription Kit (Thermo Fischer Scientific, Waltham, MA, USA). For miRNA amplification, a TaqMan Fast Advanced Master Mix (Applied Biosystems, Thermo Fischer Scientific, Waltham, MA, USA) was used. The relative expression levels of miRNAs were computed using the ΔΔCt method. qRT-PCR was performed on a ViiA™ 7 System (Thermo Fischer Scientific, Waltham, MA, USA) in a 5 µL volume using a 384-well plate. All samples were evaluated in duplicate.

The reactions were set up as follows: the initial denaturation step at 50 °C for 2 min and 95 °C for 2 s (seconds), followed by 40 cycles of 95 °C for 1 s and 60 °C for 20 s. RNU48 and RNU6B were used as housekeeping miRNAs. For further statistical analysis, the results were processed using GraphPad Prism software v.6 (GraphPad Software, San Diego, CA, USA) [34].

Statistical analysis. Comparison of miRNA tissue levels in normal and tumoral tissues and between tumor locations was performed using the Mann–Whitney U-test for non-normally distributed data. Tissue levels of miRNAs are presented as median (IQR) (interquartile range). Analysis of miRNA tissue levels in different stages and tumor grades was performed using the Kruskal–Wallis test for data with non-normal distribution, followed by post-hoc Dunn’s test. Data normality was tested using the Shapiro–Wilk test. All mentioned statistical analyses were performed using XLstat software, version 2021.3.1 (from Addinsoft, New York, NY, USA) [35]. *p* < 0.05 was considered statistically significant.

To identify the transcription factors that regulate the expression of miRNAs, the online tool TransMir v. 2.0 [36] was used. The functional analysis of this network was performed using Cytoscape v. 10.2 [37] with the Reactome FI plug-in [38]. The TF-gene-miRNA network was built using the miRNet online tool, v. 2.0 [39]. The pathway analysis of common transcription factors between miRNAs was completed using the ShinyGo online software, v.0.80 [40].

## 3. Results

The biological and clinicopathological characteristics of the patients are presented in Table 1.

**Figure 1:**
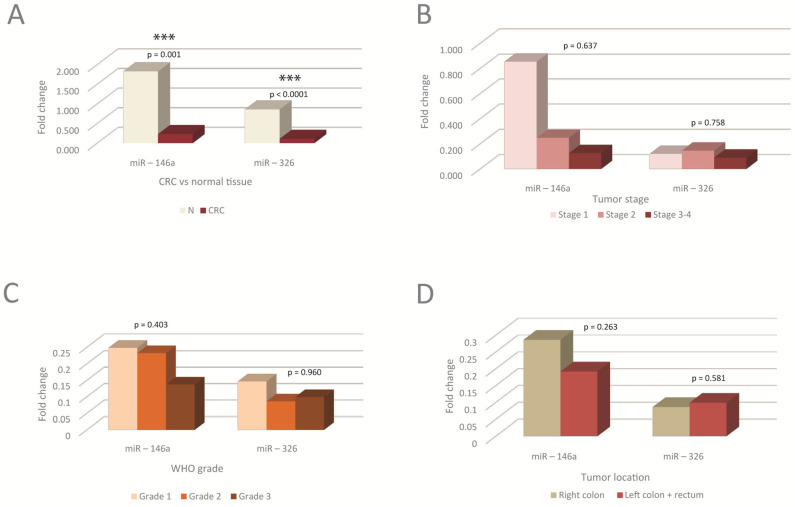
The tissue levels of miRNAs in the normal versus tumor tissue, as well as in different tumor stages, WHO grades, and tumor locations. (**A**) Tissue levels of miRNAs in tumoral compared to normal tissue. miRNAs were significantly downregulated in tumoral tissue, N-normal; (**B**–**D**) Tissue levels of miRNAs in different tumor TNM stages (**B**), WHO tumor grading (**C**), and tumor locations (**D**); no statistically significant differences were observed between miRNAs levels in different stages, WHO grades or tumor locations. G1, G2, G3-tumor WHO grade. MiRNA level is expressed as fold change (FC). *** *p* < 0.001. WHO-World Health Organization.

The tissue levels of miRNAs in normal versus tumor tissue, as well as in different stages, tumor WHO grades, and locations, are presented in Figure 1.

Significant differences were observed between tumoral compared to normal tissue, the levels in tumors being lower than in the corresponding normal tissue (Figure 1A). No significant differences were observed between miRNA levels in different tumor stages, grades, or locations. There was, however, a trend toward a decrease in miR-146a with the tumor stage and grade, as well as in left tumor locations (Figure 1B–D).

**Figure 2:**
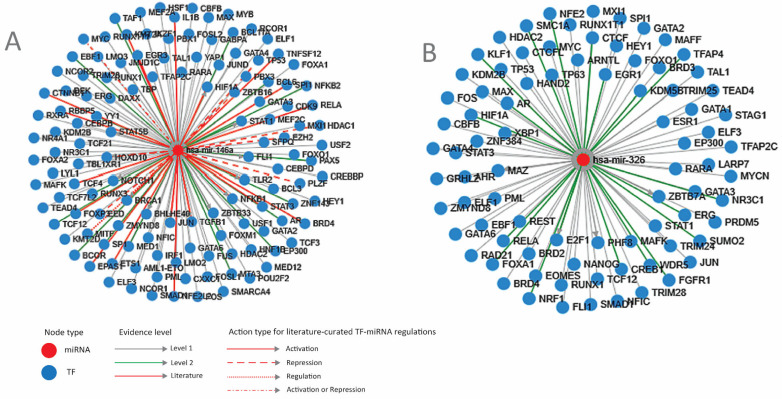
Transcription factors that regulate the two miRNAs. (**A**) miR-146A; (**B**) miR-326. miR-micro-RNA; Level 1—TF-miRNA regulation derived from ChIP-sequencing; Level 2 promoter—supported by high-throughput experimental data; blue circle—TF (transcription factor); red circle—mi-RNA (micro-RNA) (TransMir, v.2.0) [36].

Transcription factors that regulate miRNA expression. Next, we investigated the cellular mechanisms that control miRNA expression. First, an analysis of the miRNA regulation through transcription factors was performed. The results are shown in Figure 2A,B and also in Tables S1 and S2.

An overview of these transcription factors shows that most of them regulate proliferation (*FOS*, *MYC*, *JUN*, *FOXM1*) and differentiation (*RUNX1*, *GATA2*, *FLI1*, *FOXA1*) pathways, transcription *(TAF1*, *MED1*, *SFPQ*) or chromatin (*HDAC2*, *SMARCA4*). Some of them are genome-protective TFs (*BRCA1*, *TP53*) or regulators of inflammation and immunity (*NFKB1*, *IL1B*, *STAT3*), while others contribute to other cellular responses such as hypoxia (*HIF1A*), temperature (*HSF1*), or endoplasmic reticulum stress (*XBP1*).

The expression of all TFs in the intestinal epithelium, as well as in CRC, was checked using TCGA and The Human Protein Atlas [41,42]. TFs that are not expressed in the intestine and do not change expression in CRC, such as *NANOG*, *GATA4*, *ETS1*, *IKZF1*, *TAL1*, *MAFK*, *TFAP4*, or *KLF1*, were not considered for further analysis. TFs that direct differentiation in the non-intestinal tissues and have low or no expression in the colon, such as *GATA3*, *CEBPB*, *BCL6*, *PBX1*, *HEY1*, and *PU1*, were also excluded. TFs that direct differentiation in non-intestinal tissue but have expression in the colon and significant modifications in CRC, such as hepatic factors *HNF1A* and *FOXA1* or melanocytic master TF *MITF*, were considered for further analysis.

**Figure 3:**
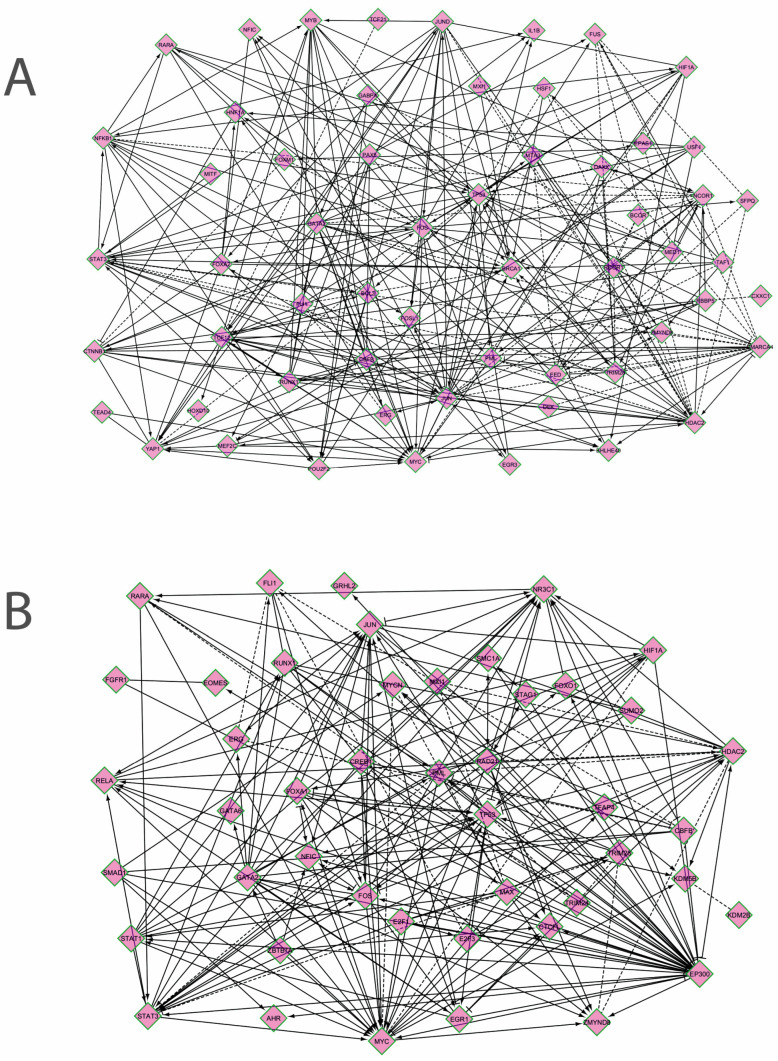
Correlation networks between the transcription factors that regulate the expression of miR-146a (**A**) and miR-326 (**B**). The genes are presented in the following order, in both networks, from left to right: genes in signaling pathways, lineage-specific or differentiation transcription factors, genes of the immediate-early response and of the cell cycle, DNA-damage-response genes, TFs that regulate chromatin, TFs that regulate transcription, others (inflammatory, hypoxia or stress response, metabolic TFs) (Cytoscape with Reactome FI plug-in) [37,38].

*Correlation network between TFs that regulate miRNAs.* To characterize the organization and the degree of correlation within the cellular system that regulates the two miRNAs, the correlation network between the TFs from the previous analysis was analyzed. Due to the fact that the two miRNAs are differentially expressed in different tissue compartments in the intestine, two different networks were built, one for each miRNA. To accurately represent biological correlations between TFs, directional networks were built using the Reactome FI plug-in of Cytoscape. The results are presented in Figure 3A,B, and also in Tables S3 and S4.

It can be seen that in both networks, the following directional relations are established: genes in signaling pathways, such as STAT1, SMAD1, TEAD4, or TCF12, regulate lineage or differentiation TFs (RUNX1, AHR, GATA2, HOXD10), which interact with the immediate-early response genes and cell cycle TFs (*JUN*, *FOS*, *MYC*, *EGR1*, *E2F1*); differentiation TFs, as well as cell cycle TFs, regulate transcription (*TFAP4*, *DEK*, *MED1*, *SFPQ*) and chromatin regulators (*HDAC2*, *ZMYND8*, *EED*, *KDM2B*); these genes, in turn, regulate the expression of many genes from signaling pathways and cell cycle (*SMARCA4 → MYC*; *EP300 → FOS*, *MYC*, *STAT3*, *RELA*, *AHR*; *HDAC2 ─│ MYC*, *FOS*, etc.); cell cycle TFs regulate DNA-damage-response genes (*TP53*, *BRCA1*). Correlations are also established between the TLR (Toll-like receptors)-activated genes and the hypoxia and DNA-damage response (*HIF1A-NFKB1-TP53*, *HSP-BRCA*). Certain signaling pathways genes exhibit correlations between them (*NFKB1-YAP1*, *STAT3-YAP1*, *RARA-NFKB1-STAT3*) (Figure 3A,B). There are fewer inverse correlations, where cell cycle genes such as *FOS*, *MYC*, or *E2F1* may regulate pathway genes (*CTNNB*, *STAT3*) or differentiation TFs such as *HNF1A* or *FOXA1*.

Overall, the correlation network that coordinates miR-146a had an average of 8,48 neighbors for each node and a clustering coefficient of 0,276. The top five hub genes in the order of the in-degree (regulated by many genes) were *TP53* (in-degree 20), *STAT3* (17), *SMARCA4* (15), *YAP1* (14), and *TCF12* (13). The top five hub genes in the order of the out-degree (which controls many genes) were *HDAC2* (out-degree 18), *FOS* (16), *BRCA1* (16), *JUN* (14), and *MYC* (12). MiR-326 network had 9,8 neighbors for each node and a clustering coefficient of 0,189. The top five genes keeping into account the in-degree were *TP53* (in-degree 17), *STAT3* (15), *MYC* (11), *NR3C1* (10), and *JUN* (9), and the top five out-degree genes were *EP300* (out-degree 27), *MYC* (13), *HDAC2* (13), *FOS* (12), and *CREB1* (10) [37,38].

**Figure 4:**
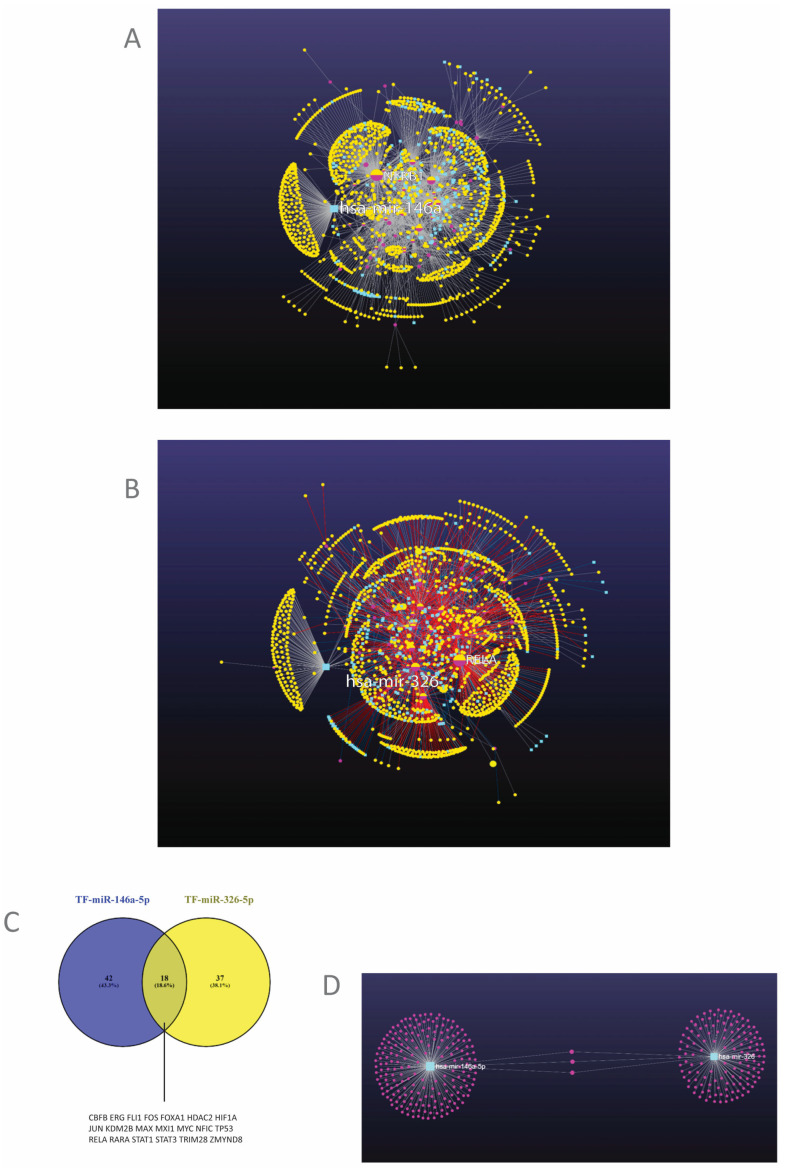
(**A**,**B**). The networks controlled by the TFs that regulate miRNAs. (**A**) The network in which miR-146a is regulated. (**B**) the network in which miR-326 is regulated. Light blue squares-miRNAs; mauve circles-transcription factors; yellow circles-genes. (**C**) Common TFs that can regulate both miRNAs. TF-transcription factor. (**D**) The targets of the two miRNAs. Light blue squares-miRNAs; mauve circles-genes (miRNet v. 2.0.) [39].

TF targets: genes and miRNAs. MiRNAs perform their roles in complex molecular networks and environments. Consequently, to have a proper understanding of their functions, they may be better studied in their biological context, considering the whole ensemble in which they perform their functions. Therefore, in the next step, we explored the networks coordinated by the TFs from the above analysis, comprising both target genes and target miRNAs of these TFs. MiR-146a and miR-326, together with their target genes, were analyzed as modules in the context of their networks. The results are presented in Figure 4A,B.

The analysis revealed two networks with a modular structure, in which each module was centered by one of the analyzed TFs and of the two miRNAs and included the target genes and miRNAs of each one of these. Functional analysis of each module was subsequently performed. The most relevant of the modules are presented in Table 2, together with their hub genes and the functional analysis of each module. The complete list of modules, with component genes and miRNAs, is presented for each network in Tables S5 and S6.

It can be seen that for the TFs, the analysis highlighted their regulatory role through a few sets of target genes organized in modules. The functions regulated by TFs through these modules are, in the case of miR-146a-5p, signaling pathways such as MAPK, TGFβ, JAK-STAT, WNT, or calcium signaling pathways; cell cycle, p53 or DNA damage-related processes; transcription regulation; cell adhesion; metabolic pathways (glycolysis, fatty acid synthesis, PPAR- peroxisome-proliferator-activated receptor- pathway), as well as inflammatory processes such as TLR, NLR (Nod-like receptors), chemokine, BCR (B-cell receptor) signaling, or leukocytes extravasation. Except for TP53 and ERG, the role of the other TFs in the mentioned pathways is generally stimulatory.

The network that generates miR-326, through its modules, also regulates a large array of functions, of which some are similar to the miR-146a network (MAPK, TGFβ, STAT pathways, cell cycle, and p53 pathway, transcription, cell adhesion, apoptosis) due to some common modules (modules 1–11) (Table 2). However, this network shows a predominance of metabolic regulation such as mucin, glycan, amino-acid, lipid, or glucose metabolic pathways, as well as cell adhesion, cytoskeleton, and cell motility regulation. Inflammatory-regulated elements like TLR, BCR, FCεR (FC-epsilon receptor for Immunoglobulin E) pathways, cytokine-receptor interaction, and leukocyte adhesion are also present in this network which also positively regulates signaling pathways, anabolism, and synthesis in the ER (endoplasmic reticulum), as well as inflammation.

MiRNA targets. miR-146a and miR-326 coordinate distinct modules in their network, which represent the target genes of these miRNAs (Figure 4A,B,D, Tables S5–S8). A pathway analysis of these modules is presented in Table 3. The relevant pathways in CRC and immunity are presented, in order of the FDR (False discovery rate). The complete list of functions and pathways targeted by the two miRNAs can be found in Tables S7 and S8.

It can be seen that miR-146a negatively regulates, within its network, inflammation (TLR pathway), proliferation pathways such as NOTCH or WNT, cell adhesion, and apoptosis. Meanwhile, miR-326, in its network, targets proliferation and developmental pathways (Hedgehog, TGFβ), cell adhesion and metabolic pathways (fatty acid synthesis, insulin pathways), transcription, and inflammatory or immune pathways such as complement, coagulation, FCγR (FC-gamma receptor for Immunoglobulin G) phagocytosis, or BCR pathway.

MiRNA co-regulation. To investigate the degree to which the two miRNAs are co-regulated and/or share common targets, a correlation analysis was performed first (Table 4).

The two molecules exhibited a good correlation with each other, stronger in the normal tissue.

To obtain an insight into how this co-regulation takes place, an analysis of the TFs that regulate both miR-146a and miR-326 was performed, which is presented as a Venn diagram in Figure 4C. There was an 18,6% superposition between the factors that regulate the two miRNAs (Figure 4C). Some of these common TFs that can regulate both miRNAs are *JUN*, *FOS*, *MYC*, *MAX*, *TP53*, *HDAC2*, *KDM2B*, *RELA*, *STAT1*, *STAT3*, and HIF1A (Figure 4C). A pathway analysis was performed on these TFs, which is presented in Table 5.

The analysis showed that the TFs that can regulate both miRNAs are present in proliferative, inflammatory, and immune pathways. The tight correlation between miRNAs may be explained by their co-regulation through these pathways with importance in cancer, particularly in CRC.

The targets of the two miRNAs, both common and specific to each one, are presented in Figure 4D. There were only three shared targets between the two miRNAs, which were *NOTCH1*, *NOTCH2*, and *CCND1* (Figure 4D, Tables S5 and S6).

**Figure 5:**
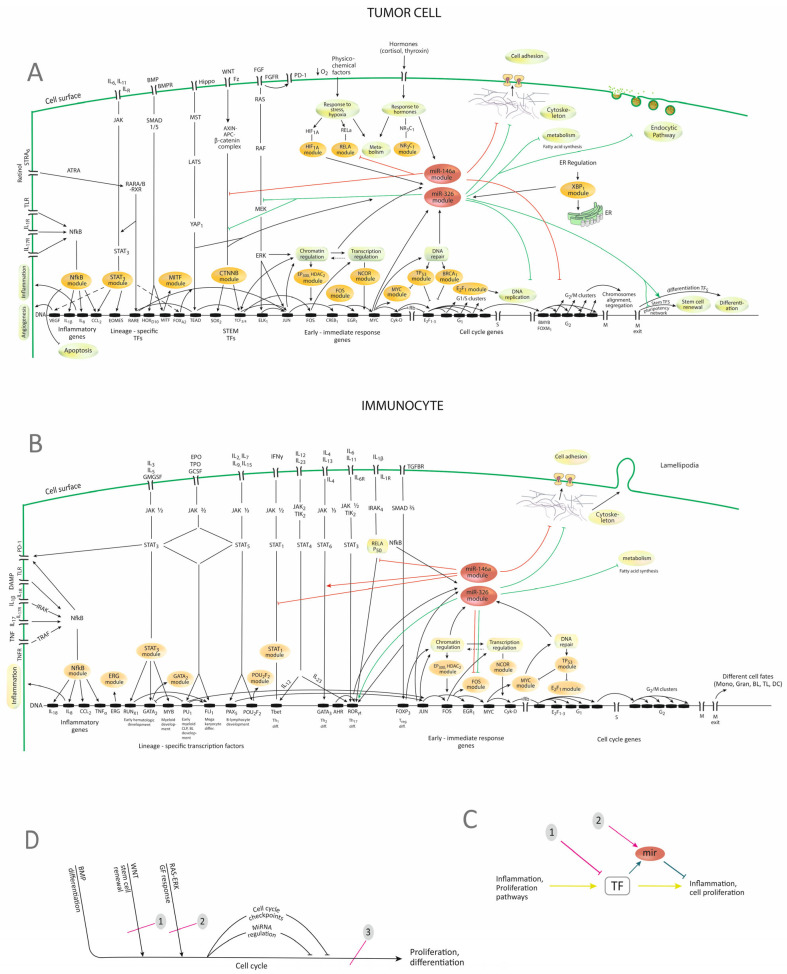
Proposed model concerning the regulatory networks that control the two miRNAs. (**A**) Representation of TF-genes-miRNAs networks in tumor cells. (**B**) Representation of TF-genes-miRNAs networks in immunocytes. The stimuli travel through the signaling pathways to the genome, where differentiation TFs are activated. These, in different combinations, coupled with the immediate-early response genes activate the successive stages in the cell cycle, ending with cell proliferation or differentiation. Other cellular responses, such as response to stress, hypoxia, and hormones, may occur as well, leading to metabolic or transcriptional responses. The present model integrates the correlations and the modules that the study highlighted (Figure 3, Table 2). It shows TFs and the corresponding modules as being correlated, explaining the correlations by their position in pathways and the cell cycle. (**C**) Schematic presentation of the regulatory relations within the network, with possible intervention strategies. The constitution of the networks allows both inhibitory interventions on the TFs (1) or approaching the network by using miRNAs (2). (**D**) Schematic diagram presenting the general organization of pathways and genomic activation, with possible intervention strategies. It can be seen that all the pathways that signal proliferation and differentiation converge on a common pathway—the cell cycle. This may open therapeutic opportunities. 1,2-inhibition of individual pathways; 3-inhibition of the cell cycle. JAK—Janus-kinase; miR, miRNA—micro-RNA; IL—interleukin; EPO—erythropoietin; TPO—thrombopoietin; GMCSF—granulocyte-macrophage colony-stimulating factor; GCSF—granulocyte colony-stimulating factor; DAMP—danger-associated molecular pattern; TNF—tumor-necrosis factor; IRAK—interleukin-1-receptor-associated kinase; TRAF—TNF-receptor-associated factor; TGF—transforming-growth factor; SMAD—Similar to Mother-against-decapentaplegic; STRA6—stimulated by retinoic acid receptor-6; ATRA—all-trans-retinoic acid; RARA—retinoic acid receptor encoding gene; RXR—retinoid X receptor; RARE—retinoid acid response element; ITG—integrin; FAK—Focal-adhesion kinase; TF—transcription factor; TLR—toll-like receptor; TLR—toll-like receptors; PD-1—programmed-death-1 receptors; VEGF—vascular endothelial growth factor; G1, S, G2, M—stages of the cell cycle; ER—endoplasmic reticulum; Th1, Th2, Th-17—T-helper 1,2 and 17 lymphocytes; Mono—monocytes; Gran—granulocytes; BL—B lymphocyte; TL—T lymphocyte; DC—dendritic cell; miR—micro-RNA; TF—transcription factor; GF—growth factor; red lines—regulation of targets by miR-146a; green lines—regulation of targets by miR-326; brown ovals—modules of TFs according to Table 2; → stimulation; ─ inhibition. The model was built based on the data from the present study and also on other data [4,5,15,21,22,23,24,43,44]. The position of different TFs in the diagram was confirmed by comparison to KEGG pathways (05200N, 05210N, 04110N, 05202, 04659 and 05235) [40,45] (Figure S1). Correlations between different TF were represented according to the analysis that was performed which is presented in Figure 3A,B.

Finally, a *model* that summarizes the data from the study is presented (Figure 5).

The diagrams show that TFs are generated in a set of pathways that may be grouped into inflammatory, proliferative pathways, as well as responses to stress, hypoxia, or hormones. In the meantime, the activation of TFs, caused by the activation of the pathway to which they belong, will increase the expression of miRNAs, including miR-146a and miR-326, through which the system is fine-tuned and regulation and balance between functions are achieved. The diagrams also highlight possible implications of the observed decrease in the two miRNAs, which may be a reduced inhibition of proliferation or, in the case of miR-146a, of inflammation. A certain organization of pathways is also highlighted, with the convergence of many proliferation, differentiation, or developmental pathways (RAS-ERK, WNT, NOTCH, Hippo, BMP) on a unique final effector which is the cell cycle. It can also be noticed that in the proposed model, the modules from the analysis (Table 2) string out along the proliferation and differentiation pathways so that interfering with many of them becomes a question of interfering with one or two pathways.

**Figure 6:**
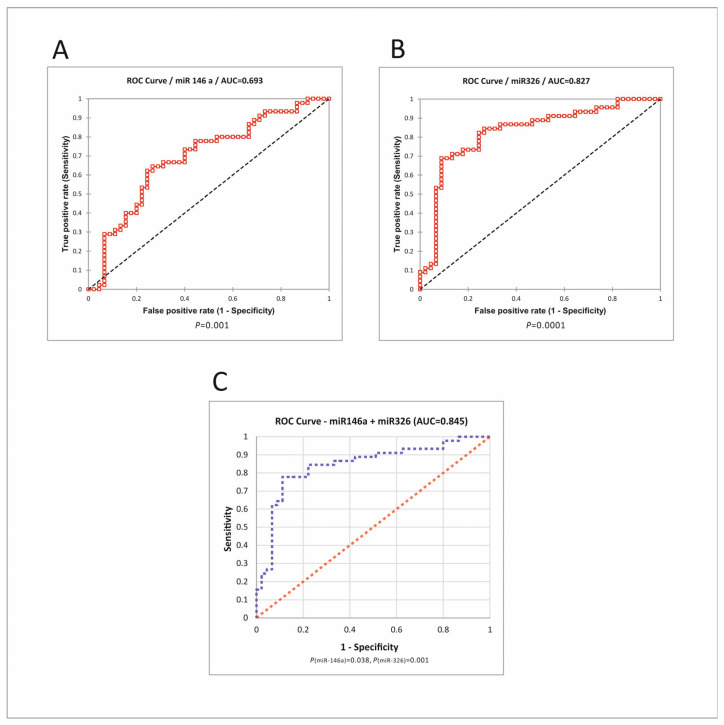
ROC analysis of miRNAs. (**A**) Roc curve for miR-146a. (**B**) Roc curve for miR-326. (**C**) Logistic regression for the combination of two miRNAs. *P*(miR-146), *P*(miR-326)—a statistical measure of each miRNA’s contribution to the regression model. ROC—receiver operating characteristic; AUC—area under the curve [35].

Biomarker potential of miRNAs. To investigate the biomarker potential of the two miRNAs, alone or in combination, ROC (Receiver operating characteristic) analysis was performed. The results are presented in Figure 6.

MiR-326 had a good biomarker potential, with an AUC (Area under the curve) of 0.827, 0.911 sensitivity (Sn), and 0.689 specificity (Sp) at the optimal cutoff value of 0.289. MiR-146a had less discriminative potential, with an AUC of 0.693. The combination of miRNAs had a slightly better performance than miR-326 alone, with an AUC of 0.845, Sn of 0.75, and Sp of 0.89.

The analysis of the biomarker potential was performed in different tumor TNM stages and locations; the results are presented in Table 6 and Figure S1.

The best performance was recorded for miR-326 and the combination of two, in all stages and locations. In the early stages, there was a *Sn* of 0.91, with *Sp* approaching 0.7. In the later stages, there was a slightly better potential of miR-326 and of the two miRNAs, with *Sn* reaching 0.91 at *Sp* of 0.76. The analysis by locations showed a better performance in the right colon tumors, with *Sn* of 0.952 and *Sp* of 0.76 (Table 6 and Figure S2).

## 4. Discussion

MiRNAs have been intensely studied as potential biomarkers or therapeutic targets in CRC and other cancers [2]. Network approaches are increasingly frequent in the study of genomic processes in cancer [46]. The present study considered two miRNAs with roles in the tumor immunity and cell cycle of immune and intestinal cells- miR-146a and miR-325.

The level of miR-146a in tissues was found to be significantly lower in tumor tissue compared to paired adjacent normal tissue. Other authors found decreased levels of miR-146a in tumoral tissue [14,47], while others have found increased levels [48,49]. These variations in the level of this anti-inflammatory miRNA, which is synthesized under the influence of the inflammatory pathways [4], could be due to the variability of the development of the inflammatory compartment in CRC. In a previous study [50], we found decreased levels of IL-10, as well as high levels of IL-8, reflecting a predominance of inflammation over anti-inflammatory activity in these tumors, while others found different levels of pro-inflammatory or anti-inflammatory cytokines [51,52], outlining a picture of extreme heterogeneity in CRC immunology. Another explanation may be the global reduction in miRNAs in cancer [53], due to epigenomic silencing, among others [54].

The tissue level of miR-326 was found to be reduced in CRC in our study, which is generally consistent with what others have found [55]. miR-326 is an oncosuppressive miRNA [56], which may also explain its low level in tumors. The expression of the two miRNAs has not been significantly correlated to the tumor stage, grade, or tumor location (Figure 1), which likely points to the homogeneity of mechanisms of these miRNAs in tumors, regardless of the stage or grade that the tumors present.

Regarding the mechanisms of the two miRNAs, as the present study has shown, their synthesis is controlled by a network of transcription factors that belongs to the inflammatory and immune pathways, as well as to the proliferative and differentiation processes.

In this general framework, miRNAs constitute a module in the corresponding TF network, where they have their own targets in these and related pathways. Their role in this system would be the fine-tuning of functions, quantitative regulation, or the inhibition of competing functions (e.g., miR-326 stimulates certain differentiations, in the meantime inhibiting the cell cycle) [57]. Thus, miRNAs might be considered as a derivative pathway with a role in regulating the main pathway (Figure 5C).

As for the expression and role in CRC, the evidence is that the two miRNAs are located in the immune, as well as in the epithelial compartments of tumors [10,19], being correspondingly activated by immune and proliferation pathways [4,14,16], which the present study has also highlighted. Their targets are immune and non-immune, a fact that the present study and others [3] have shown. Although the analyzed miRNAs were shown to act as immuno-miRNAs [5,15,25], due to the complex regulation of each one of them, as well as the wide range of targeted biological functions (Figure 4A,B), their role cannot be restrained to the immune system, but encompasses a host of other functions, particularly in cell cycle and proliferation [14,22]. The immune role and cell cycle inhibition may be connected during LT differentiation, the latest process involving cell cycle and stem gene inhibition [25].

In spite of the tight correlation between the two miRNAs (Table 3), the analysis of their regulatory mechanisms showed a low degree of co-regulation (Figure 4C). Since there are not many TFs that regulate both miRNAs, the explanation could concern not the TFs themselves, but the mechanisms that control them. Indeed, in a previous study, we showed that there is a high degree of connectivity in the network that regulates cell proliferation, which may explain the tight correlation between the miRNAs [58].

The constitution of the network also permits some therapeutic considerations. If, for example, inhibition of inflammation or the cell cycle is desired, the main axis TFs-target genes may be approached for inhibition, (Figure 5C,D), but the miRNA loop may also be used (Figure 5C).

Concerning the first approach, the functional relations between TFs have to be understood as much as possible, through both bioinformatic and experimental approaches. In this regard, the convergence of pathways on the cell cycle that was highlighted in the present study (Figure 5A–C) may have significance concerning approach strategies. Indeed, some of the therapeutic failures of pathway inhibition, which were explained by the activation of secondary pathways, caused either by the apparition of mutations in these other pathways or by communications between pathways that may bypass the inhibition [59,60], might be overcome by choosing to inhibit a common pathway where all stimuli converge, such as the cell cycle (Figure 5B). There are indeed promising results, for example, in the therapy of breast cancer, by using inhibitors of CDK 4/6 [61].

Concerning the second approach, miR-146a has already been proposed as an anti-inflammatory mean [15], while both miRNAs are considered for cancer therapy [30].

MiRNAs have also been studied as biomarkers in various tumors, including CRC. In the present study, we found a good biomarker potential of miR-326, as well as of the combinations of two miRNAs (Figure 6), including for the early stages (Table 6). In a setting in which the golden standard of colonoscopy with histopathological examination may have failures, with dramatic consequences [62], the use of additional criteria such as miRNA tissue levels may add precision to the diagnostic.

In a previous study, we showed that there is a high level of connectivity in the cellular networks that ensures cell proliferation, which was explained by the causal succession of the signaling and transcriptional events that are at the basis of these processes [58]. The therapeutic implication is that this flow of events may be targeted as a unique network. The present study adds details concerning the configuration of the system, which may address some of the current setbacks in targeted pathway therapy (Figure 5C,D).

The present study also adds details concerning various correlations between TFs, which could also be used in targeted approaches (Figure 3A,B and Figure 5A,B). For example, genes in pathways that have many targets, such as *CTNNB1*, *STAT3*, or *RAF*, genes downstream of pathways that may drive proliferation and have also many targets such as *FOS* or *MYC* or chromatin regulators such as *EP300* or *HDAC2* are potential targets that have been considered for cancer therapy [63,64,65]. Differentiation-inducing TFs are not suitable targets, as they may reverse one of the cancer hallmarks which is disrupted differentiation [66]. Factors such as the out-degree, connexions, and position in the detailed and overall network of a certain gene may be considered in target selection, along with the analysis of the individually activated or mutated pathway.

One of the limitations of the study is represented by the relatively small sample size. Although the results are consistent with literature data, confirmation on larger patient samples would be necessary before translation into the clinic. Another limitation is linked to the fact that the configuration of the signaling and transcriptional networks was established through bioinformatic analysis and did not benefit from experimental confirmations such as knock-down or overexpression of miRNAs or interfering with transcription factors or signaling pathways. The analysis brings contributions to the understanding of the pathways involved in CRC. It remains, however, to be confirmed through the appropriate experimental approaches.

A weak point of the study is method-specific: as with any pathway analysis, the present analysis lacks tissue specificity. This disadvantage was partially compensated for by the selection and analysis of tissue-specific TFs and the exclusion of TFs with no known role and expression in the intestine and in CRC. The study does not explain the low levels of the two miRNAs in CRC. However, the consequences of their decrease, as well as of their eventual therapeutical usage, may be estimated from the presented data and model.

## 5. Conclusions

MiR-146a and miR-326 exhibited decreased levels in CRC compared to normal tissue.

The two miRNAs were regulated in the context of the immune and proliferative pathways, in which they perform anti-proliferative roles. Concerning inflammation, they played opposite roles, with miR-146a regulating pro-inflammatory targets, while miR-326 differentiates inflammatory cells. This suggests that the two miRNAs are associated with both the immune compartment in tumors and with tumor cell biology.

The study highlights the role of the two miRNAs in the overall transcriptional regulatory networks of the cell, and also suggests potential approach strategies that take into account the configuration of these regulatory networks. In this regard, both miRNAs may have potential as antiproliferative drugs, while miR-146a may play an anti-inflammatory role.

MiR-326, as well as the combination of two miRNAs, showed a good biomarker potential for CRC.

In a context where miRNAs are increasingly being considered for both diagnostic and therapy of cancer, inflammation, and other diseases, the present study shows that indeed miRNAs that were considered, in the context of their networks, may have a utility in both diagnostic and treatment of colorectal tumors and associated inflammation.

## Figures and Tables

**Table 1:** The clinicopathological characteristics of the patients.

1. Mean age (years)		67.51 (11.4)
2. Sex	Male	21 (46.66%)
	Female	24 (53.33%)
3. Tumor histology	Adenocarcinoma	45 (100%)
4. Tumor TNM stage	1	6 (13.33%)
	2	19 (42.22%)
	3–4	20 (44.44%)
5. Tumor WHO grade	1	9 (20%)
	2	27 (60%)
	3	9 (20%)
6. Tumor location	Right colon	21 (46.66%)
	Left colon	19 (42.22%)
	Rectum	5 (11.11)
7. Comorbid conditions	Inflammatory conditions (RA, IBD)	0 (0%)
	Diabetes mellitus	5 (11.11%)
	Cirrhosis	1 (2.22)
	Ascites	1 (2.22)
	CKD	1 (2.22)
	Chronic bronchitis	2 (4.44)

[1–7]—Mean (standard deviation); WHO—World Health Organization; RA—rheumatoid arthritis; IBD—inflammatory bowel disease; CKD—chronic kidney disease.

**Table 2:** Modules in the two networks and associated functions and pathways.

miR-146a Network			miR-326 Network	
No.	Module (Hub Gene)	Functions (KEGG Pathways)	Module (Hub Gene)	Functions (KEGG Pathways)
1	*FOS*	MAPK signaling pathway Pathways in cancer Osteoclastic differentiation Colorectal cancer Cytokine–cytokine receptor interaction Apoptosis	*FOS*	MAPK signaling pathway Pathways in cancer Osteoclastic differentiation Colorectal cancer Cytokine–cytokine receptor interaction Apoptosis
2	*MYC*	Cell cycle Antigen processing P53 signaling pathway Cell adhesion molecules Colorectal cancer	*MYC*	Cell cycle Antigen processing P53 signaling pathway Cell adhesion molecules Colorectal cancer
3	*ERG*	Leukocytes transendothelial migration Cell adhesion molecules Pathways in cancer NK-mediated cytotoxicity Rheumatoid arthritis	*ERG*	Leukocytes transendothelial migration Cell adhesion molecules Pathways in cancer NK-mediated cytotoxicity Rheumatoid arthritis
4	*TP53*	P53 pathway Cell cycle NLR pathway Pathways in cancer	*TP53*	P53 pathway Cell cycle NLR pathway Pathways in cancer
5	*NFIC*	ECM-ligand interaction Focal adhesion Steroid hormone biosynthesis Purine metabolism	*NFIC*	ECM-ligand interaction Focal adhesion Steroid hormone biosynthesis Purine metabolism
7	*HDAC2*	p53 signaling pathway TGFβ signaling pathway Cell cycle Transcription misregulation in cancer	*HDAC2*	P53 signaling pathway TGFβ signaling pathway Cell cycle Transcription misregulation in cancer
8	*STAT3*	Jak-STAT signaling pathway Influenza A Hepatitis C Complement and coagulation cascade P53 signaling Pathways in cancer	*STAT3*	Jak-STAT signaling pathway Influenza A Hepatitis C Complement and coagulation cascade P53 signaling Pathways in cancer
9	*GATA2*	Asthma Fatty acid metabolism FCεRI pathway Leucocyte transendothelial migration	*GATA2*	Asthma Fatty acid metabolism FCεRI pathway Leucocyte transendothelial migration
10	*HIF1A*	Pathways in cancer Glycolysis, gluconeogenesis Toxoplasmosis TLR signaling pathway	*HIF1A*	Pathways in cancer Glycolysis, gluconeogenesis Toxoplasmosis TLR signaling pathway
11	*miR-335*	Cytokine–cytokine receptor interaction	*miR-335*	Cytokine–cytokine receptor interaction
12	*CTNNB1*	Colorectal cancer Pathways in cancer Adheens junction WNT signaling pathway	*EOMES*	P53 signaling pathway Hepatitis C Cell cycle
13	*TAF1*	Erbb signaling pathway HTLV1 infection	*EGR1*	Glycosamine degradation HTLV1 infection Focal adhesion Autoimmune thyroid disease
14	*GABPA1*	Calcium signaling pathway	*CREB1*	Dopaminergic synapse Antigen processing and presentation Complement and coagulation cascade Calcium signaling pathway Fatty acid biosynthesis
15	*POU2F2*	Antigen processing Cell adhesion molecules	*MYCN*	Antigen processing and presentation Epstein Barr virus infection Cell cycle NK mediated cytotoxicity Neutrophylin signaling
16	*MITF*	Melanogenesis Pathways in cancer BCR signaling pathway	*MAX*	Cell adhesion molecules Cytokine–cytokine receptor interaction Regulation of actin cytoskeleton Leukocyte transepithelial migration Tight junction
17	*FOSL1*	ECM-receptor interaction Cytokine–cytokine receptor interaction Cell cycle TLR signaling pathway Pathways in cancer	*E2F1*	Cell cycle P53 signaling pathway Rig-1-like receptor pathway Pathways in cancer
18	*BRCA1*	Measles JAK-STAT signaling pathway Tuberculosis TLR signaling pathway	*EP300*	Pathways in cancer HTLV-1 infection Cell cycle TGFβ signaling pathway Bile secretion Notch signaling pathway
19	*MXI1*	Cytokine–cytokine receptor interaction Measles Regulation of actin cytoskeleton Leukocyte transendothelial migration Tight junction	*PML*	Pathways in cancer Colorectal cancer P53 signaling pathway Apoptosis
20	*EED*	Cell cycle Pathways in cancer Oocyte meiosis p53 signaling pathway Rheumatoid arthritis Linoleic acid metabolism	*ZBTB7A*	Glycolysis/gluconeogenesis Pentose phosphate pathway Pyruvate metabolism P53 signaling pathway Cell cycle Pathways in cancer
21	*USF1*	Leishmaniasis Antigen processing and presentation Glycolysis-gluconeogenesis	*NR3C1*	Phenylalanine metabolism P53 signaling pathway
22	*NCOR1*	Transcription misregulation in cancer PPAR signaling pathway P53 signaling pathway Adherens junction	*NRF1*	RNA transport Citrate cycle Mucin glycan A biosynthesis
23	*HSF1*	Legionellosis NK mediated cytotoxicity	*XBP1*	Protein processing in the endoplasmic reticulum Aminoacid metabolism
24	*NFKB1*	Cytokine–cytokine receptor interaction Chemokine signaling pathway NLR pathway Intestinal immune network for IgA secretion	*RELA*	Cytokine–cytokine receptor interaction Chemokine signaling pathway NLR pathway Apoptosis Intestinal immune network for IgA secretion
25			*STAT1*	TLR receptor signaling Hepatitis C Cytokine-receptor interaction JAK-STAT pathway Intestinal immune network for IgA secretion

TGFβ—Transforming growth factor beta; NLR—nod-like receptor; TLR—toll-like receptor; ECM—extracellular matrix; MAPK—mitogen-activated protein-kinase; FCεRI—FC-epsilon receptor for Immunoglobulin E; NK—natural killer (lymphocyte); BCR—B-cell receptor; HTLV-1—human T-cell lymphotropic virus-1; PPAR—peroxisome-proliferator-activated receptor. FDR cutoff was set to 0.05. (miRNet v. 2.0) [39].

**Table 3:** Function of miRNAs-regulated target genes.

MiRNA	Pathway (KEGG)	FDR
miR-146a module	TLR signaling	0.029
	Adherens junction	0.029
	Notch signaling pathway	0.029
	Axon guidance	0.029
	RIG-1 receptor signaling	0.029
	Colorectal cancer	0.029
	Apoptosis	0.029
	Neutrophin signaling pathway	0.029
	Pertussis	0.029
	Chagas disease	0.030
	WNT signaling pathway	0.047
miR-326 module	Hedgehog signaling pathway	0.111
	Insulin pathway signaling	0.263
	Vibrio cholerae infection	0.308
	Tight junction	0.308
	Fatty acid biosynthesis	0.501
	Notch signaling pathway	0.501
	Dorso-ventral axis formation	0.55
	Complement and coagulation cascade	0.55
	Adherens junction	0.55
	B-cell receptor signaling pathway	0.55
	Transcriptional misregulation in cancer	0.55
	TGFβ signaling pathway	0.556
	Systemic lupus erythematosus	0.559
	FCγR-mediated phagocytosis	0.608
	Regulation of actin cytoskeleton	0.608

FDR—False discovery rate; TLR—toll-like receptor; FCγR—FC-gamma receptor for Immunoglobulin G (miRNet v.2.0) [39].

**Table 4:** Correlation analysis between the two miRNAs.

Correlation	Normal Tissue	Tumoral Tissue
MiR-146a-miR-326	0.844 *	0.797 *

* Spearman correlation coefficient [35].

**Table 5:** Pathway analysis of the transcription factors that regulate both miRNAs.

Pathway	FDR
Pathways in cancer	3.6 × 10^−10^
Transcriptional misregulation in cancer	5 × 10^−9^
Th17 differentiation	7.1 × 10^−9^
PD-1 expression and PD-L regulation in cancer	1.7 × 10^−7^
Colorectal cancer	7.9 × 10^−6^
MAPK pathway	3.6 × 10^−5^
Micro-RNAs in cancer	6.9 × 10^−5^

FDR—false discovery rate; Th17—T-helper17 lymphocyte; PD-1—programmed-death receptor-1. Only the top seven pathways with relevance in CRC are presented. The FDR cutoff was set to 0.05. [40].

**Table 6:** The biomarker potential of miRNAs in different tumor TNM stages and locations.

Clinical Parameter		Micro-RNA	AUC	Sensitivity	Specificity
TNM stage	Stages 1–2	miR-146a	0.655	0.76	0.56
		miR-326	0.808	0,91	0.68
		miR146a_ + miR-326	0.824	0.76	0.89
	Stages 3–4	miR-146a	0.741	0.69	0.80
		miR-326	0.850	0.91	0.77
		miR146a_ + miR-326	0.872	0.91	0.76
Tumor location	Right colon	miR-146a	0.742	0.60	0.86
		miR-326	0.878	0.91	0.72
		miR146a_ + miR-326	0.888	0.952	0.76
	Left colon+ rectum	miR-146a	0.651	0.778	0.583
		miR-326	0.781	0.911	0.625
		miR146a_ + miR-326	0.807	0.75	0.89

AUC—area under the curve [35].

## Data Availability

The data that support the conclusion of this study will be made available from the authors upon reasonable request.

## Supplementary Materials

The following supporting information can be downloaded at: https://www.mdpi.com/article/10.3390/cimb46070421/s1, Table S1: Transcription factors that regulate miR-146a; Table S2: Transcription factors that regulate miR-326; Table S3: Correlations in the directed network of TFs that regulate miR-146a; Table S4: Correlations in the directed network of TFs that regulate miR-326; Table S5: Modules of miR-146a network; Table S6: Modules of miR-326 network; Table S7: Functions of miR-146a module; Table S8: Functions of miR-326 module; Figure S1: KEGG pathways that were used to build the models from the present study; Figure S2: ROC analysis of miRNAs in different tumor TNM stages and locations.
