# Supplementary material for: Expression and Functional Analysis of Immuno-Micro-RNAs mir-146a and mir-326 in Colorectal Cancer
Source: Curr Issues Mol Biol. 2024 Jul 5;46(7):7065–85. doi: 10.3390/cimb46070421 (PMC11276483; doi:10.3390/cimb46070421)
Supplement: Supplementary file 1 [file cimb-46-00421-s001.zip › Figure S1- KEGG pathways that were used for model building.pdf]

Figure S1- KEGG pathways that were used for model building [41,42]

A-Pathway 05200N-Pathways in cancer

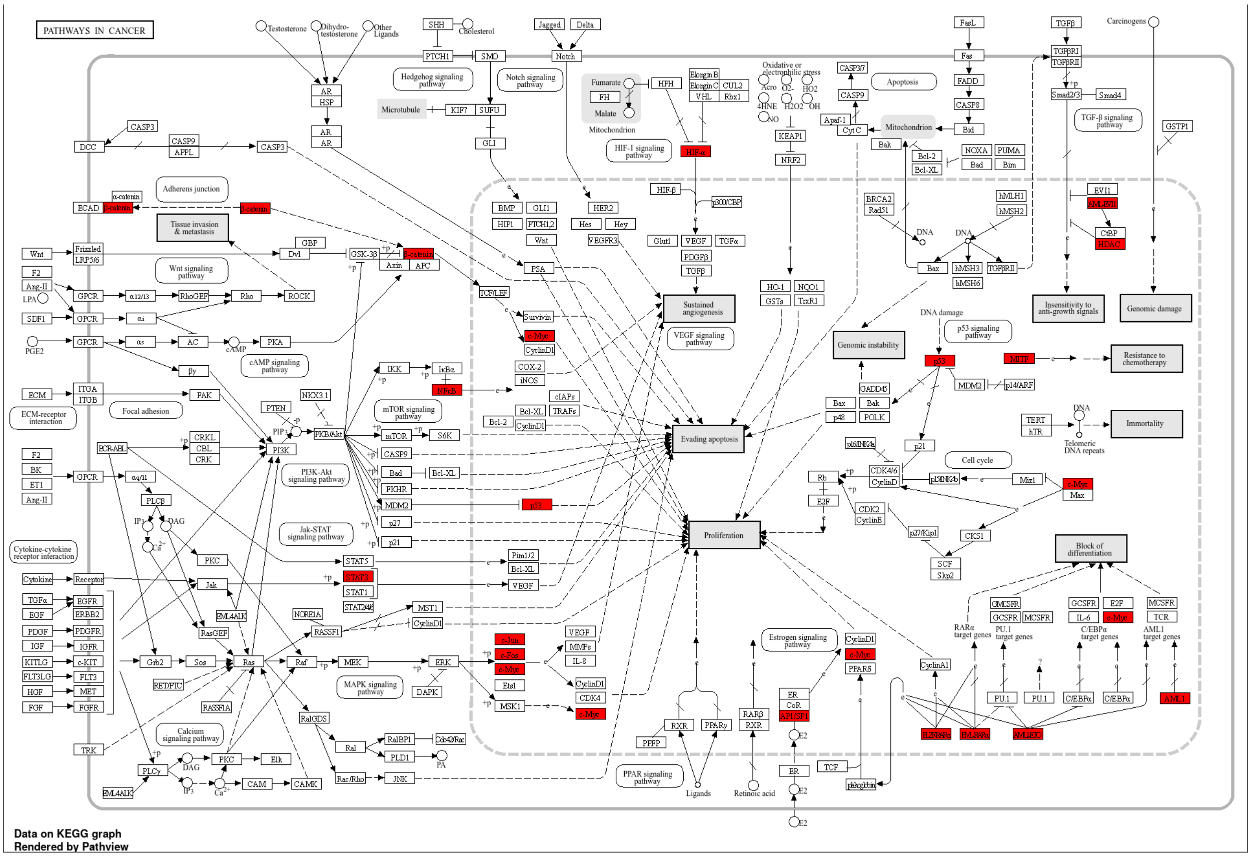

B-Pathway 05210N-Colorectal cancer.

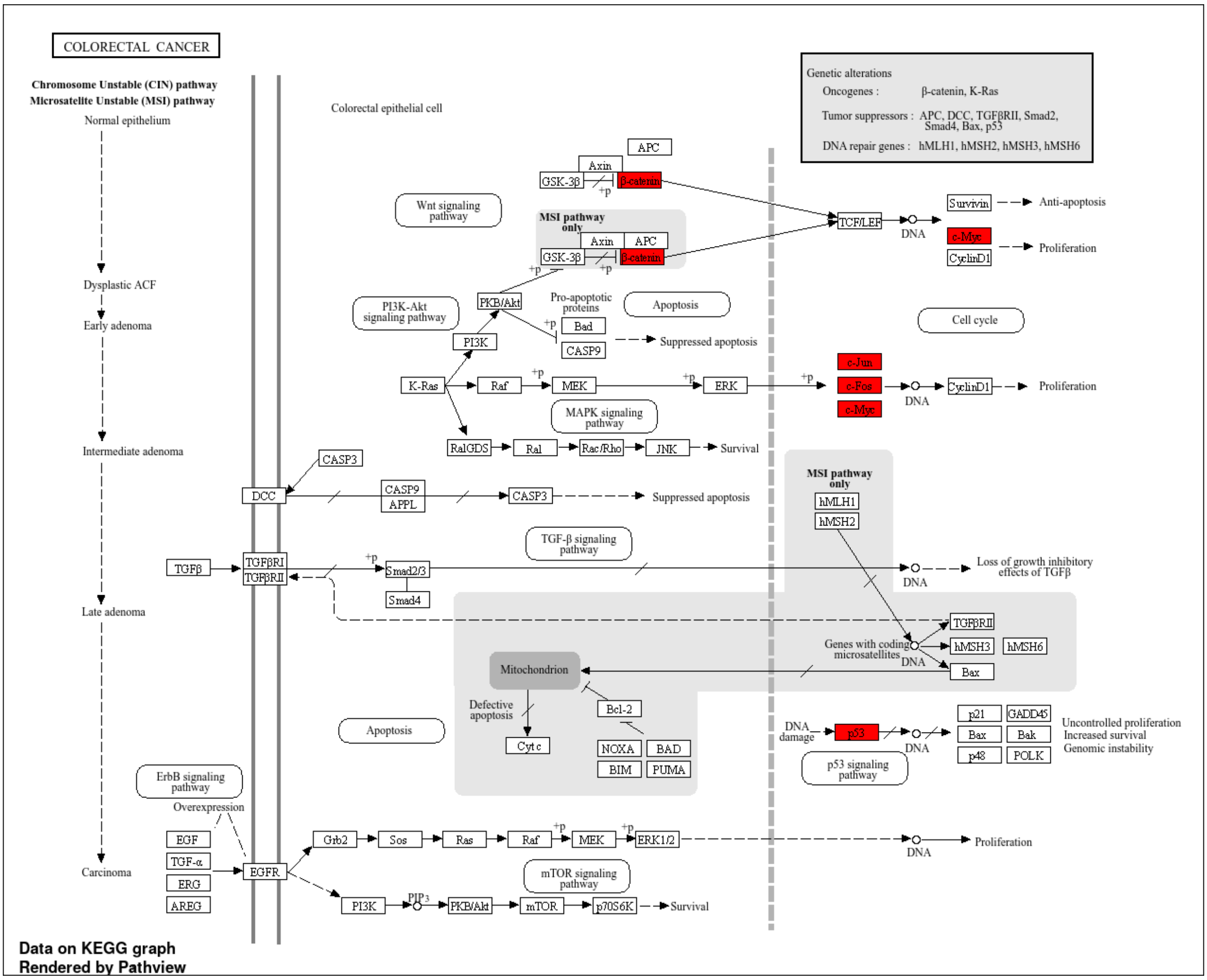

C-Pathway 04110N-Cell cycle.

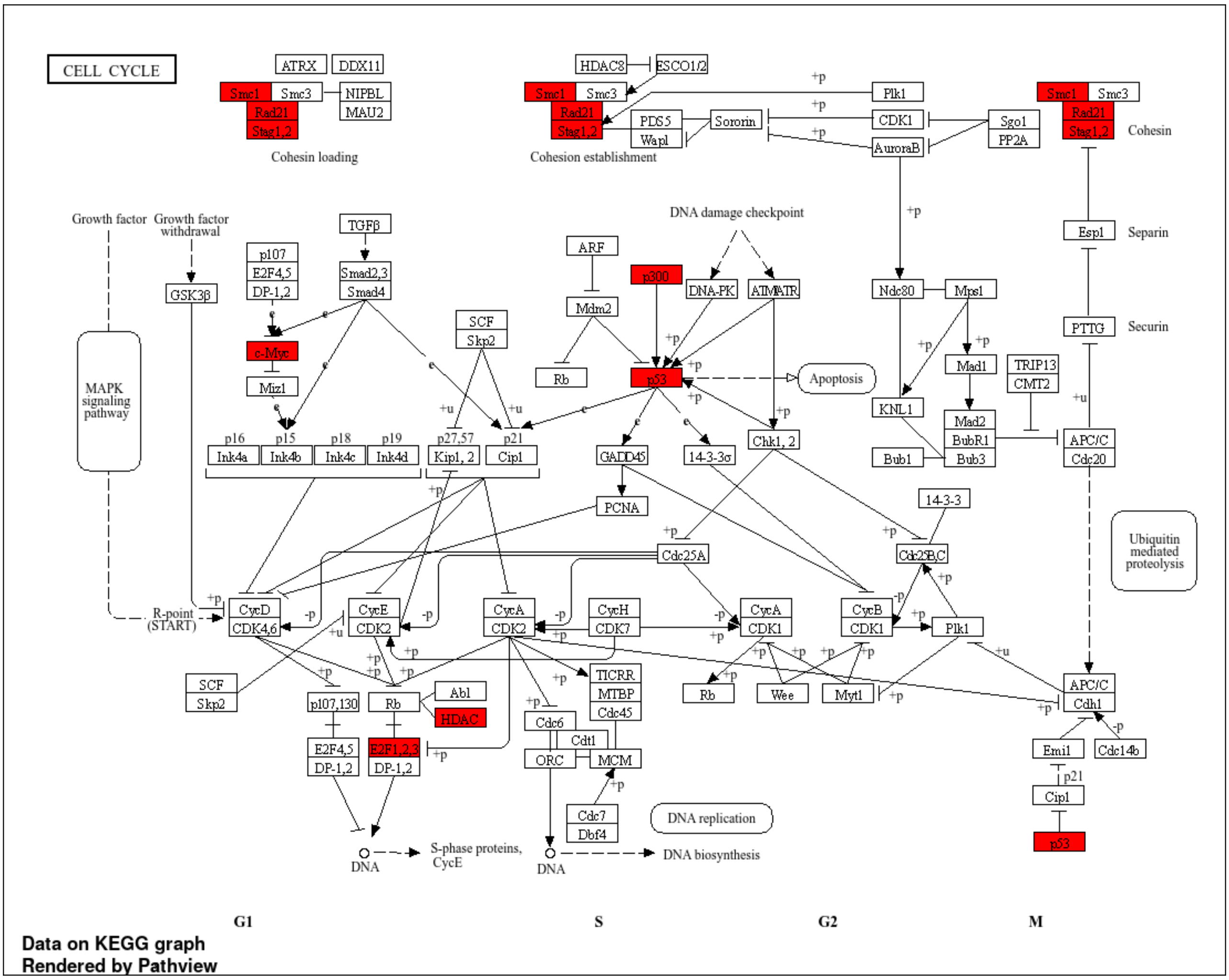

D-Pathway 05202-Transcription misregulation in cancer

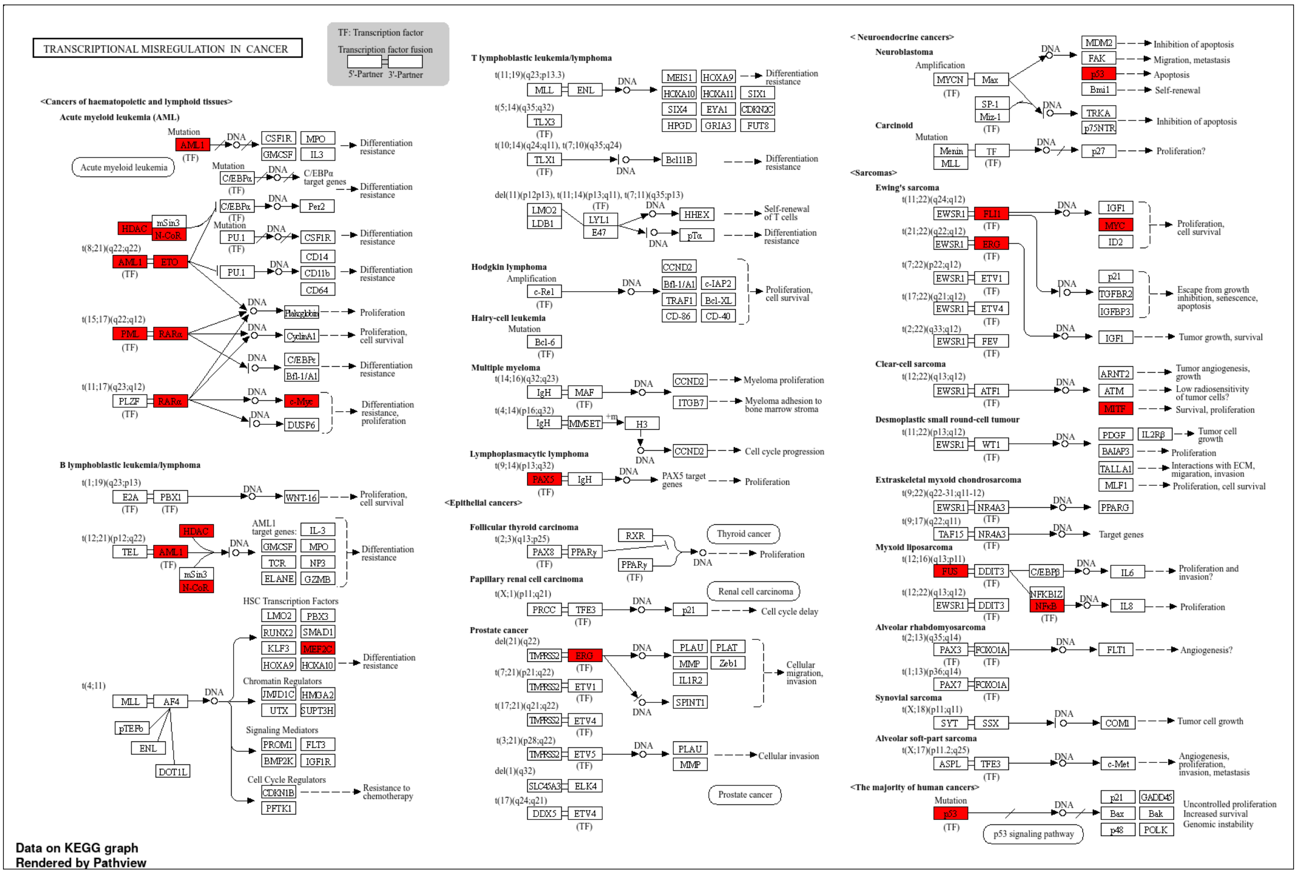

E-Pathway 04659-Th17 differentiation.

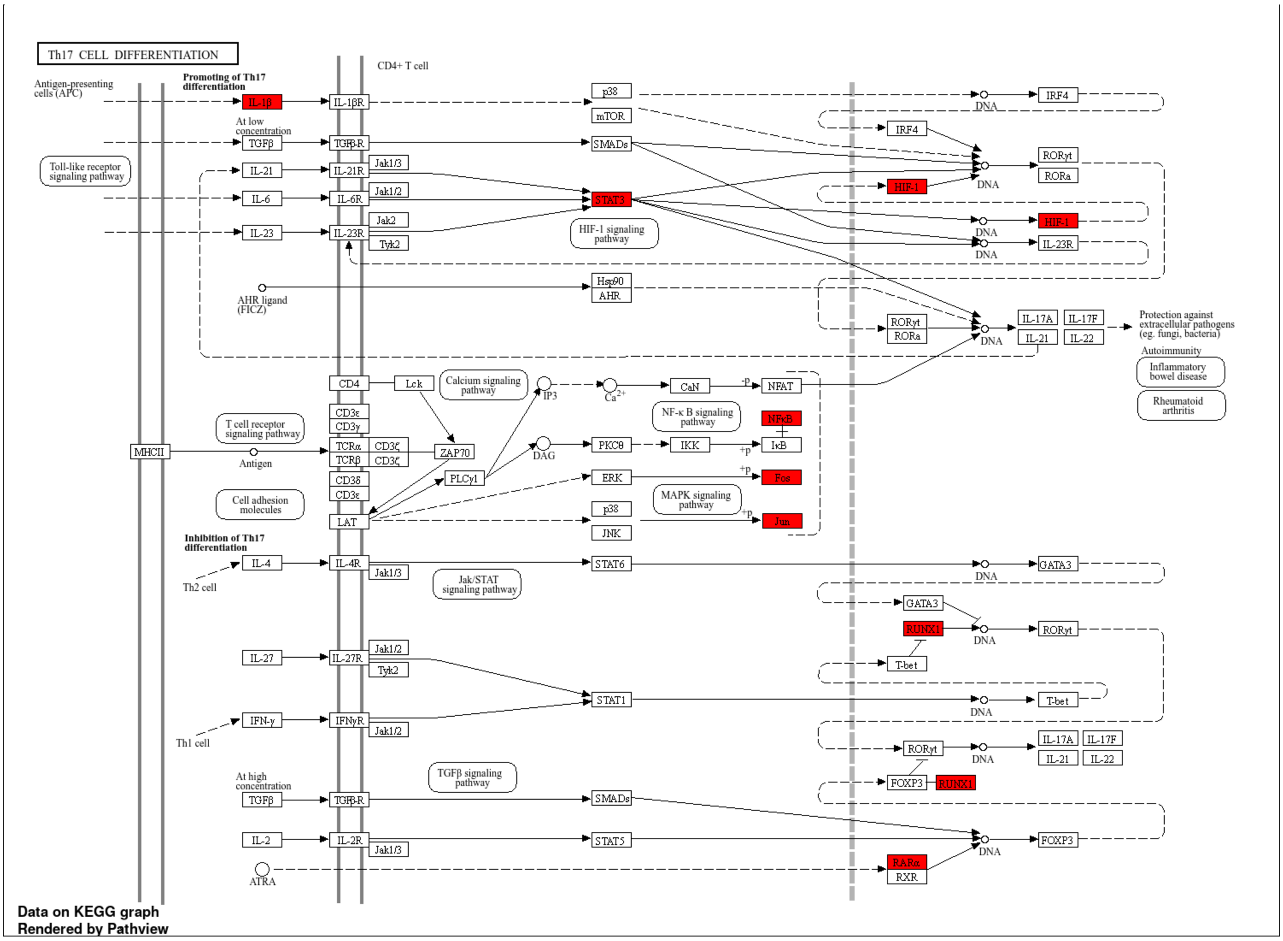

F-Pathway 05235-PD-L1 expression and PD-1 checkpoint pathway in cancer .

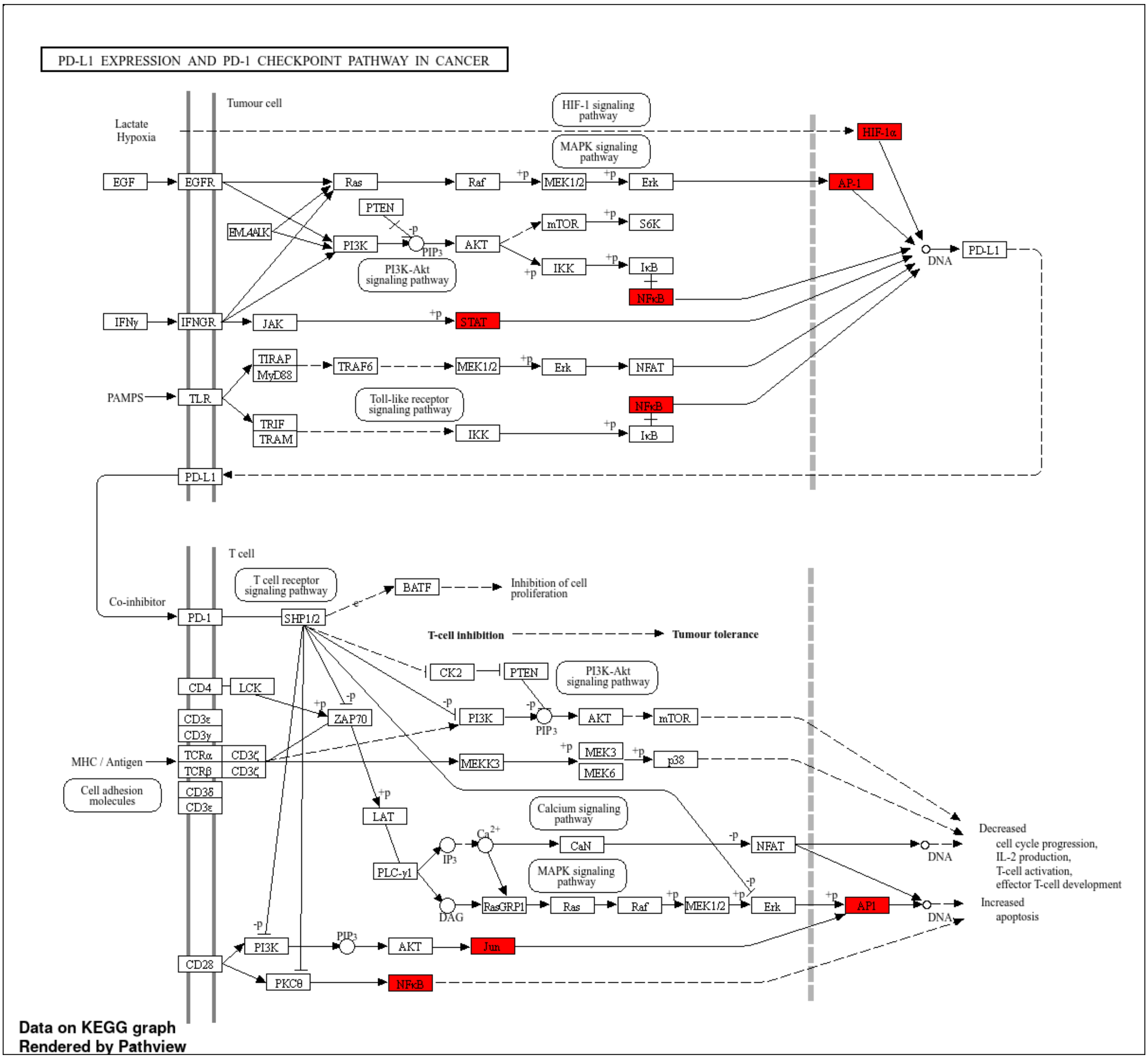

Figure S1-KEGG pathways that were used to build the model. A-Pathway 05200N-Pathways in cancer; B-Pathway 05210N- Colorectal cancer; C-Pathway 04100N-Cell cycle; D-Pathway 05202-Transcriptional misregulation in cancer; E-Pathway 04659-Th17 differentiation; F- Pathway 05235- PD-L1 expression and PD-1 checkpoint pathway in cancer [41,42]. In red-analyzed genes.
