# Supplementary material for: Expression and Functional Analysis of Immuno-Micro-RNAs mir-146a and mir-326 in Colorectal Cancer
Source: Curr Issues Mol Biol. 2024 Jul 5;46(7):7065–85. doi: 10.3390/cimb46070421 (PMC11276483; doi:10.3390/cimb46070421)
Supplement: Supplementary file 1 [file cimb-46-00421-s001.zip › Figure S2- ROC analysis of miRNAs in different tumor TNM stages and locations..pdf]

Figure S2- ROC analysis of miRNAs in different tumor stages and locations.

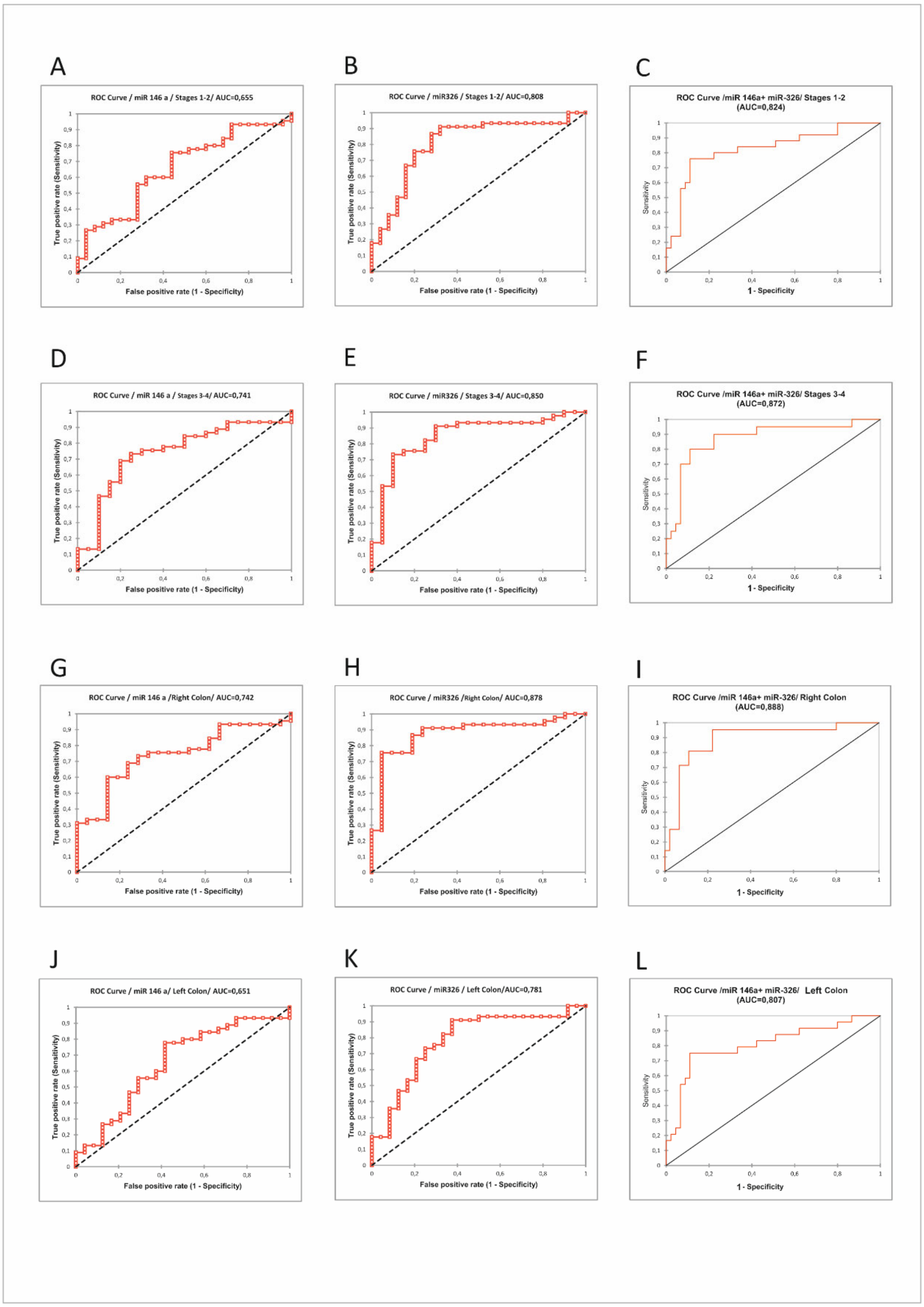

Figure S2. A-C: ROC curves of miRNAs in stages 1-2; A-miR-146a; B-miR-326; C-miR-146a+miR-326. D-F: ROC curves of miRNAs in stages 3-4; D-miR-146a; E-miR-326; F-miR-146a+miR-326. G-I: ROC curves of miRNAs in right colon tumors; G-miR-146a; H-miR-326; I-miR-146a+miR-326. J-L: ROC curves of miRNAs in left colon tumors; J-miR-146a; K-miR-326; L-miR-146a+miR-326. ROC-Receiver operating characteristics; AUC- Area under the curve [35].
