# Supplementary material for: Expression and Functional Analysis of Immuno-Micro-RNAs mir-146a and mir-326 in Colorectal Cancer
Source: Curr Issues Mol Biol. 2024 Jul 5;46(7):7065–85. doi: 10.3390/cimb46070421 (PMC11276483; doi:10.3390/cimb46070421)
Supplement: Supplementary file 1 [file cimb-46-00421-s001.zip › Table S1-Transcription factors that regulate the expression of mir-146a.pdf]

Table S1-Transcription factors that regulate the expression of mir-146a

TF name	miRNA name	TSS	Binding site	Action type	SRAID/PMID	Evidence	Tissue	Species
AML1-ETO	hsa-mir-146a	chr5: 160468251(NR_132748)	chr5: 160467620-160467967(score=1000)	Regulation	SRX259613	level 1	Blood	H.sapiens
AML1-ETO	hsa-mir-146a	chr5: 160468251(NR_132748)	chr5: 160467650-160467932(score=724)	Regulation	SRX259614	level 1	Blood	H.sapiens
AML1-ETO	hsa-mir-146a	chr5: 160468251(NR_132748)	chr5: 160467697-160467897(score=523)	Regulation	SRX1756593	level 1	Blood	H.sapiens
AR	hsa-mir-146a	chr5: 160468252	chr5: 160467998-160468159(score=523)	Regulation	SRX433197	level 2	Prostate	H.sapiens
AR	hsa-mir-146a	chr5: 160468251(NR_132748)	chr5: 160467836-160468177(score=864)	Regulation	SRX433201	level 1	Prostate	H.sapiens
AR	hsa-mir-146a	chr5: 160468251(NR_132748)	chr5: 160467838-160468173(score=502)	Regulation	SRX433198	level 1	Prostate	H.sapiens
AR	hsa-mir-146a	chr5: 160468251(NR_132748)	chr5: 160467910-160468161(score=440)	Regulation	SRX433202	level 1	Prostate	H.sapiens
BCL11A	hsa-mir-146a	chr5: 160468252	chr5: 160468095-160468249(score=605)	Regulation	SRX100433	level 2	Blood	H.sapiens
BCL11A	hsa-mir-146a	chr5: 160468251(NR_132748)	chr5: 160467725-160467869(score=418)	Regulation	SRX100433	level 1	Blood	H.sapiens
BCL3	hsa-mir-146a	chr5: 160468251(NR_132748)	chr5: 160467731-160467941(score=772)	Regulation	SRX100387	level 1	Blood	H.sapiens
BCL6	hsa-mir-146a	chr5: 160468252	chr5: 160468115-160468295(score=428)	Regulation	SRX130608	level 2	Blood	H.sapiens
BCL6	hsa-mir-146a	chr5: 160468252	chr5: 160468144-160468310(score=355)	Regulation	SRX084599	level 2	Blood	H.sapiens
BCL6	hsa-mir-146a	chr5: 160468251(NR_132748)	chr5: 160467717-160468074(score=418)	Regulation	SRX1012539	level 1	Blood	H.sapiens
BCL6	hsa-mir-146a	chr5: 160468251(NR_132748)	chr5: 160467722-160467960(score=449)	Regulation	SRX1012538	level 1	Blood	H.sapiens
BCL6	hsa-mir-146a	chr5: 160468251(NR_132748)	chr5: 160467730-160468155(score=1000)	Regulation	SRX689470	level 1	Blood	H.sapiens
BCL6	hsa-mir-146a	chr5: 160468251(NR_132748)	chr5: 160467766-160467919(score=382)	Regulation	SRX130608	level 1	Blood	H.sapiens
BCL6	hsa-mir-146a	chr5: 160468251(NR_132748)	chr5: 160467815-160467967(score=415)	Regulation	SRX244191	level 1	Blood	H.sapiens
BCL6	hsa-mir-146a	chr5: 160468251(NR_132748)	chr5: 160468222-160468389(score=648)	Regulation	SRX689470	level 1	Blood	H.sapiens
BCOR	hsa-mir-146a	chr5: 160468252	chr5: 160468068-160468253(score=510)	Regulation	SRX130609	level 2	Blood	H.sapiens
BCOR	hsa-mir-146a	chr5: 160468251(NR_132748)	chr5: 160467743-160467997(score=536)	Regulation	SRX130609	level 1	Blood	H.sapiens

BCOR hsa-mir-146a chr5: 160468251(NR_132748) chr5: 160467743-
160468051(score=613) Regulation SRX689472 level 1 Blood H.sapiens
BHLHE40 hsa-mir-146a chr5: 160468251(NR_132748) chr5: 160467633-
160468224(score=793) Regulation(feedback) SRX150509 level 1 Blood
H.sapiens
BRCA1 hsa-mir-146a n/a n/a Activation(feedback) 19190326
literature n/a H.sapiens
BRCA1 hsa-mir-146a n/a n/a Regulation(feedback) 22936386
literature n/a H.sapiens
BRCA1 hsa-mir-146a n/a n/a Activation(feedback) 25417703
literature n/a H.sapiens
BRD4 hsa-mir-146a chr5: 160468252 chr5: 160468079-
160468350(score=326) Regulation SRX204903 level 2 Blood H.sapiens
BRD4 hsa-mir-146a chr5: 160468251(NR_132748) chr5: 160467657-
160468148(score=1000) Regulation SRX204905 level 1 Blood H.sapiens
BRD4 hsa-mir-146a chr5: 160468251(NR_132748) chr5: 160467718-
160467829(score=238) Regulation ERX378355 level 1 Blood H.sapiens
BRD4 hsa-mir-146a chr5: 160468251(NR_132748) chr5: 160467633-
160467833(score=324) Regulation SRX2267440 level 1 Epidermis
H.sapiens
BRD4 hsa-mir-146a chr5: 160468251(NR_132748) chr5: 160463787-
160464061(score=806) Regulation SRX327751 level 1 Neural
H.sapiens
BRD4 hsa-mir-146a chr5: 160468251(NR_132748) chr5: 160463842-
160464041(score=627) Regulation SRX327752 level 1 Neural
H.sapiens
BRD4 hsa-mir-146a n/a n/a Activation 26855180 literature n/a
H.sapiens
CBFB hsa-mir-146a chr5: 160468251(NR_132748) chr5: 160467707-
160468010(score=826) Regulation SRX265219 level 1 Blood H.sapiens
CBFB hsa-mir-146a chr5: 160468251(NR_132748) chr5: 160467749-
160467964(score=405) Regulation SRX265238 level 1 Blood H.sapiens
CDK9 hsa-mir-146a chr5: 160468251(NR_132748) chr5: 160467551-
160467826(score=486) Regulation SRX203391 level 1 Blood H.sapiens
CDK9 hsa-mir-146a chr5: 160468251(NR_132748) chr5: 160467761-
160467921(score=272) Regulation SRX244177 level 1 Blood H.sapiens
CEBPB hsa-mir-146a chr5: 160468251(NR_132748) chr5: 160463785-
160463973(score=520) Regulation SRX1823809 level 1 Bone H.sapiens
CEBPB hsa-mir-146a chr5: 160468251(NR_132748) chr5: 160463796-
160464013(score=548) Regulation SRX1823810 level 1 Bone H.sapiens
CEBPB hsa-mir-146a chr5: 160468251(NR_132748) chr5: 160463831-
160463956(score=766) Regulation SRX190345 level 1 Breast
H.sapiens
CEBPB hsa-mir-146a chr5: 160468251(NR_132748) chr5: 160463801-
160463998(score=977) Regulation SRX190308 level 1 Digestive tract
H.sapiens
CEBPB hsa-mir-146a chr5: 160468251(NR_132748) chr5: 160463825-
160463982(score=732) Regulation SRX096352 level 1 Digestive tract
H.sapiens
CEBPB hsa-mir-146a chr5: 160468251(NR_132748) chr5: 160463826-
160463975(score=546) Regulation SRX096351 level 1 Digestive tract
H.sapiens

CEBPB hsa-mir-146a chr5: 160468251(NR_132748) chr5: 160463811-160463992(score=1000) Regulation SRX150572 level 1 Liver H.sapiens
CEBPB hsa-mir-146a chr5: 160468251(NR_132748) chr5: 160463818-160463970(score=1000) Regulation SRX150701 level 1 Liver H.sapiens
CEBPB hsa-mir-146a chr5: 160468251(NR_132748) chr5: 160463830-160463978(score=1000) Regulation SRX190234 level 1 Liver H.sapiens
CEBPB hsa-mir-146a chr5: 160468251(NR_132748) chr5: 160463781-160464041(score=1000) Regulation SRX150598 level 1 Lung H.sapiens
CEBPB hsa-mir-146a chr5: 160468251(NR_132748) chr5: 160463815-160463993(score=708) Regulation SRX150709 level 1 Lung H.sapiens
CEBPB hsa-mir-146a chr5: 160468251(NR_132748) chr5: 160463839-160463978(score=703) Regulation SRX190327 level 1 Lung H.sapiens
CEBPB hsa-mir-146a chr5: 160468251(NR_132748) chr5: 160463766-160464062(score=1000) Regulation SRX150632 level 1 Uterus H.sapiens
CEBPB hsa-mir-146a chr5: 160468251(NR_132748) chr5: 160463841-160463955(score=529) Regulation SRX190258 level 1 Uterus H.sapiens
CEBPD hsa-mir-146a chr5: 160468251(NR_132748) chr5: 160463762-160464047(score=1000) Regulation SRX2355057 level 1 Cardiovascular H.sapiens
CREBBP hsa-mir-146a chr5: 160468251(NR_132748) chr5: 160467639-160468320(score=1000) Regulation SRX2339193 level 1 Blood H.sapiens
CREBBP hsa-mir-146a chr5: 160468251(NR_132748) chr5: 160467662-160468280(score=1000) Regulation SRX2339191 level 1 Blood H.sapiens
CTNNB1 hsa-mir-146a n/a n/a Activation 24561623 literature n/a H.sapiens
CXXC1 hsa-mir-146a chr5: 160468251(NR_132748) chr5: 160467908-160468091(score=365) Regulation SRX1424886 level 1 Blood H.sapiens
DAXX hsa-mir-146a chr5: 160468251(NR_132748) chr5: 160463889-160464031(score=466) Regulation SRX1021630 level 1 Prostate H.sapiens
DEK hsa-mir-146a chr5: 160468251(NR_132748) chr5: 160467691-160468058(score=495) Regulation SRX685530 level 1 Blood H.sapiens
EBF1 hsa-mir-146a chr5: 160468252 chr5: 160468118-160468311(score=351) Regulation SRX150455 level 2 Blood H.sapiens
EBF1 hsa-mir-146a chr5: 160468251(NR_132748) chr5: 160467632-160467959(score=976) Regulation SRX150455 level 1 Blood H.sapiens
EBF1 hsa-mir-146a chr5: 160468251(NR_132748) chr5: 160467690-160467885(score=546) Regulation SRX1456193 level 1 Blood H.sapiens
EBF1 hsa-mir-146a chr5: 160468251(NR_132748) chr5: 160467737-160467870(score=426) Regulation SRX100431 level 1 Blood H.sapiens
EBF1 hsa-mir-146a chr5: 160468251(NR_132748) chr5: 160468155-160468373(score=839) Regulation SRX100431 level 1 Blood H.sapiens
EED hsa-mir-146a n/a n/a Repression 24699235 literature n/a H.sapiens
EGR3 hsa-mir-146a n/a n/a Activation 23733368 literature n/a H.sapiens
ELF1 hsa-mir-146a chr5: 160468251(NR_132748) chr5: 160467745-160468111(score=778) Regulation SRX100541 level 1 Blood H.sapiens

ELF3 hsa-mir-146a chr5: 160468251(NR_132748) chr5: 160467712-160468317(score=677) Regulation SRX825394 level 1 Adipocyte H.sapiens
ELF3 hsa-mir-146a chr5: 160468251(NR_132748) chr5: 160467746-160468160(score=604) Regulation SRX1389378 level 1 Adipocyte H.sapiens
ELF3 hsa-mir-146a chr5: 160468251(NR_132748) chr5: 160467781-160468138(score=468) Regulation SRX1389380 level 1 Adipocyte H.sapiens
EP300 hsa-mir-146a chr5: 160468252 chr5: 160468058-160468248(score=865) Regulation SRX475753 level 2 Blood H.sapiens
EP300 hsa-mir-146a chr5: 160468252 chr5: 160468067-160468247(score=721) Regulation SRX392819 level 2 Blood H.sapiens
EP300 hsa-mir-146a chr5: 160468252 chr5: 160468068-160468270(score=622) Regulation SRX392820 level 2 Blood H.sapiens
EP300 hsa-mir-146a chr5: 160468252 chr5: 160468072-160468265(score=644) Regulation SRX150374 level 2 Blood H.sapiens
EP300 hsa-mir-146a chr5: 160468252 chr5: 160468075-160468259(score=767) Regulation SRX475754 level 2 Blood H.sapiens
EP300 hsa-mir-146a chr5: 160468252 chr5: 160468075-160468287(score=474) Regulation SRX475750 level 2 Blood H.sapiens
EP300 hsa-mir-146a chr5: 160468252 chr5: 160468076-160468274(score=725) Regulation SRX150641 level 2 Blood H.sapiens
EP300 hsa-mir-146a chr5: 160468252 chr5: 160468086-160468227(score=505) Regulation SRX475751 level 2 Blood H.sapiens
EP300 hsa-mir-146a chr5: 160468252 chr5: 160468112-160468230(score=384) Regulation SRX100432 level 2 Blood H.sapiens
EP300 hsa-mir-146a chr5: 160468251(NR_132748) chr5: 160467557-160468087(score=1000) Regulation SRX2043231 level 1 Blood H.sapiens
EP300 hsa-mir-146a chr5: 160468251(NR_132748) chr5: 160467561-160468138(score=1000) Regulation SRX265233 level 1 Blood H.sapiens
EP300 hsa-mir-146a chr5: 160468251(NR_132748) chr5: 160467606-160468305(score=1000) Regulation SRX475752 level 1 Blood H.sapiens
EP300 hsa-mir-146a chr5: 160468251(NR_132748) chr5: 160467613-160467926(score=775) Regulation SRX150374 level 1 Blood H.sapiens
EP300 hsa-mir-146a chr5: 160468251(NR_132748) chr5: 160467623-160468012(score=1000) Regulation SRX656350 level 1 Blood H.sapiens
EP300 hsa-mir-146a chr5: 160468251(NR_132748) chr5: 160467646-160467947(score=901) Regulation SRX392820 level 1 Blood H.sapiens
EP300 hsa-mir-146a chr5: 160468251(NR_132748) chr5: 160467657-160467943(score=1000) Regulation SRX475753 level 1 Blood H.sapiens
EP300 hsa-mir-146a chr5: 160468251(NR_132748) chr5: 160467661-160467927(score=1000) Regulation SRX475754 level 1 Blood H.sapiens
EP300 hsa-mir-146a chr5: 160468251(NR_132748) chr5: 160467664-160467939(score=955) Regulation SRX392819 level 1 Blood H.sapiens
EP300 hsa-mir-146a chr5: 160468251(NR_132748) chr5: 160467673-160467890(score=628) Regulation SRX475749 level 1 Blood H.sapiens
EP300 hsa-mir-146a chr5: 160468251(NR_132748) chr5: 160467673-160467902(score=631) Regulation SRX475748 level 1 Blood H.sapiens
EP300 hsa-mir-146a chr5: 160468251(NR_132748) chr5: 160467690-160467861(score=551) Regulation SRX150641 level 1 Blood H.sapiens

EP300 hsa-mir-146a chr5: 160468251(NR_132748) chr5: 160467690-160467959(score=546) Regulation SRX2043228 level 1 Blood H.sapiens
EP300 hsa-mir-146a chr5: 160468251(NR_132748) chr5: 160467693-160467946(score=853) Regulation SRX475750 level 1 Blood H.sapiens
EP300 hsa-mir-146a chr5: 160468251(NR_132748) chr5: 160467728-160467946(score=701) Regulation SRX475751 level 1 Blood H.sapiens
EP300 hsa-mir-146a chr5: 160468251(NR_132748) chr5: 160467730-160467902(score=381) Regulation SRX392808 level 1 Blood H.sapiens
EP300 hsa-mir-146a chr5: 160468251(NR_132748) chr5: 160467732-160467982(score=1000) Regulation ERX378364 level 1 Blood H.sapiens
EP300 hsa-mir-146a chr5: 160468251(NR_132748) chr5: 160467735-160468028(score=592) Regulation SRX1514284 level 1 Blood H.sapiens
EP300 hsa-mir-146a chr5: 160468251(NR_132748) chr5: 160467743-160467918(score=471) Regulation ERX378367 level 1 Blood H.sapiens
EP300 hsa-mir-146a chr5: 160468251(NR_132748) chr5: 160467759-160467908(score=330) Regulation SRX747574 level 1 Blood H.sapiens
EP300 hsa-mir-146a chr5: 160468251(NR_132748) chr5: 160467580-160467817(score=328) Regulation SRX1521303 level 1 Breast H.sapiens
EP300 hsa-mir-146a chr5: 160468252 chr5: 160468028-160468272(score=575) Regulation SRX2055844 level 2 Kidney H.sapiens
EP300 hsa-mir-146a chr5: 160468251(NR_132748) chr5: 160467521-160467823(score=645) Regulation SRX100544 level 1 Liver H.sapiens
EP300 hsa-mir-146a chr5: 160468251(NR_132748) chr5: 160463853-160464002(score=554) Regulation SRX212184 level 1 Lung H.sapiens
EP300 hsa-mir-146a chr5: 160468251(NR_132748) chr5: 160463853-160464002(score=558) Regulation SRX147007 level 1 Lung H.sapiens
EP300 hsa-mir-146a chr5: 160468251(NR_132748) chr5: 160463860-160464006(score=550) Regulation SRX212185 level 1 Lung H.sapiens
EP300 hsa-mir-146a chr5: 160468251(NR_132748) chr5: 160467704-160467882(score=399) Regulation SRX212185 level 1 Lung H.sapiens
EP300 hsa-mir-146a chr5: 160468251(NR_132748) chr5: 160463773-160464105(score=1000) Regulation SRX190219 level 1 Neural H.sapiens
EP300 hsa-mir-146a chr5: 160468251(NR_132748) chr5: 160463792-160464084(score=973) Regulation SRX100540 level 1 Neural H.sapiens
EP300 hsa-mir-146a chr5: 160468251(NR_132748) chr5: 160463872-160464035(score=278) Regulation SRX186631 level 1 Neural H.sapiens
EP300 hsa-mir-146a chr5: 160468251(NR_132748) chr5: 160463800-160464083(score=1000) Regulation SRX150579 level 1 Uterus H.sapiens
EPAS1 hsa-mir-146a n/a n/a Activation 26045293 literature n/a H.sapiens
ERG hsa-mir-146a chr5: 160468251(NR_132748) chr5: 160467692-160468007(score=1000) Regulation SRX682376 level 1 Blood H.sapiens
ERG hsa-mir-146a chr5: 160468251(NR_132748) chr5: 160467692-160468111(score=1000) Regulation SRX265230 level 1 Blood H.sapiens
ERG hsa-mir-146a chr5: 160468251(NR_132748) chr5: 160467693-160468162(score=1000) Regulation SRX026639 level 1 Blood H.sapiens

ERG hsa-mir-146a chr5: 160468251(NR_132748) chr5: 160467695-160468097(score=1000) Regulation SRX1514277 level 1 Blood H.sapiens
ERG hsa-mir-146a chr5: 160468251(NR_132748) chr5: 160467696-160468107(score=1000) Regulation SRX682375 level 1 Blood H.sapiens
ERG hsa-mir-146a chr5: 160468251(NR_132748) chr5: 160467699-160467886(score=612) Regulation SRX026660 level 1 Blood H.sapiens
ERG hsa-mir-146a chr5: 160468251(NR_132748) chr5: 160467699-160467972(score=1000) Regulation SRX026638 level 1 Blood H.sapiens
ERG hsa-mir-146a chr5: 160468251(NR_132748) chr5: 160467737-160467998(score=583) Regulation SRX026642 level 1 Blood H.sapiens
ERG hsa-mir-146a chr5: 160468251(NR_132748) chr5: 160467784-160468097(score=757) Regulation SRX026657 level 1 Blood H.sapiens
ERG hsa-mir-146a chr5: 160468251(NR_132748) chr5: 160467785-160467874(score=287) Regulation SRX026641 level 1 Blood H.sapiens
ERG hsa-mir-146a chr5: 160468251(NR_132748) chr5: 160467797-160467947(score=515) Regulation SRX026648 level 1 Blood H.sapiens
ETS1 hsa-mir-146a chr5: 160468251(NR_132748) chr5: 160467807-160467913(score=365) Regulation SRX100555 level 1 Blood H.sapiens
ETS1 hsa-mir-146a n/a n/a Activation 21738483 literature n/a H.sapiens
EZH2 hsa-mir-146a n/a n/a Repression 24699235 literature n/a H.sapiens
FLI1 hsa-mir-146a chr5: 160468251(NR_132748) chr5: 160467645-160468206(score=1000) Regulation(feedback) SRX026640 level 1 Blood H.sapiens
FLI1 hsa-mir-146a chr5: 160468251(NR_132748) chr5: 160467693-160468038(score=998) Regulation(feedback) SRX117011 level 1 Blood H.sapiens
FLI1 hsa-mir-146a chr5: 160468251(NR_132748) chr5: 160467705-160468088(score=622) Regulation(feedback) SRX117010 level 1 Blood H.sapiens
FLI1 hsa-mir-146a chr5: 160468251(NR_132748) chr5: 160467712-160468119(score=1000) Regulation(feedback) SRX1514276 level 1 Blood H.sapiens
FOS hsa-mir-146a chr5: 160468251(NR_132748) chr5: 160463871-160464073(score=909) Regulation SRX150477 level 1 Breast H.sapiens
FOS hsa-mir-146a chr5: 160468251(NR_132748) chr5: 160463871-160464092(score=888) Regulation SRX150517 level 1 Breast H.sapiens
FOS hsa-mir-146a chr5: 160468251(NR_132748) chr5: 160463884-160464070(score=923) Regulation SRX150478 level 1 Breast H.sapiens
FOS hsa-mir-146a chr5: 160468251(NR_132748) chr5: 160463892-160464081(score=782) Regulation SRX150476 level 1 Breast H.sapiens
FOS hsa-mir-146a chr5: 160468251(NR_132748) chr5: 160463837-160464125(score=658) Regulation SRX150664 level 1 Cardiovascular H.sapiens
FOS hsa-mir-146a chr5: 160468251(NR_132748) chr5: 160463860-160464112(score=550) Regulation SRX070879 level 1 Cardiovascular H.sapiens

FOSL1 hsa-mir-146a chr5: 160468251(NR_132748) chr5: 160463905-160464044(score=452) Regulation SRX2585779 level 1 Breast H.sapiens
FOSL2 hsa-mir-146a chr5: 160468251(NR_132748) chr5: 160463865-160464102(score=1000) Regulation SRX190296 level 1 Neural H.sapiens
FOXA1 hsa-mir-146a chr5: 160468251(NR_132748) chr5: 160463793-160464085(score=459) Regulation SRX825395 level 1 Adipocyte H.sapiens
FOXA1 hsa-mir-146a chr5: 160468251(NR_132748) chr5: 160467466-160467910(score=781) Regulation SRX1389379 level 1 Adipocyte H.sapiens
FOXA1 hsa-mir-146a chr5: 160468251(NR_132748) chr5: 160467493-160467943(score=979) Regulation SRX825395 level 1 Adipocyte H.sapiens
FOXA1 hsa-mir-146a chr5: 160468251(NR_132748) chr5: 160467495-160467955(score=656) Regulation SRX1389381 level 1 Adipocyte H.sapiens
FOXA1 hsa-mir-146a chr5: 160468251(NR_132748) chr5: 160466944-160467080(score=450) Regulation SRX100506 level 1 Liver H.sapiens
FOXA1 hsa-mir-146a chr5: 160468251(NR_132748) chr5: 160467513-160467822(score=983) Regulation SRX100506 level 1 Liver H.sapiens
FOXA1 hsa-mir-146a chr5: 160468251(NR_132748) chr5: 160467525-160467839(score=612) Regulation SRX100477 level 1 Liver H.sapiens
FOXA2 hsa-mir-146a chr5: 160468251(NR_132748) chr5: 160466937-160467047(score=424) Regulation SRX886445 level 1 Digestive tract H.sapiens
FOXA2 hsa-mir-146a chr5: 160468251(NR_132748) chr5: 160466977-160467078(score=434) Regulation SRX100448 level 1 Liver H.sapiens
FOXA2 hsa-mir-146a chr5: 160468251(NR_132748) chr5: 160467543-160467789(score=628) Regulation SRX100448 level 1 Liver H.sapiens
FOXA2 hsa-mir-146a chr5: 160485352 chr5: 160480806-160480936(score=639) Regulation SRX100448 level 1 Liver H.sapiens
FOXM1 hsa-mir-146a chr5: 160468252 chr5: 160468055-160468270(score=639) Regulation SRX190187 level 2 Blood H.sapiens
FOXM1 hsa-mir-146a chr5: 160468251(NR_132748) chr5: 160467646-160467928(score=769) Regulation SRX190187 level 1 Blood H.sapiens
FOXM1 hsa-mir-146a chr5: 160468251(NR_132748) chr5: 160467659-160467857(score=336) Regulation SRX185912 level 1 Breast H.sapiens
FOXO1 hsa-mir-146a chr5: 160468251(NR_132748) chr5: 160467689-160468048(score=1000) Regulation SRX1735651 level 1 Blood H.sapiens
FOXP3 hsa-mir-146a n/a n/a Activation 25712342 literature n/a H.sapiens
FUS hsa-mir-146a chr5: 160468251(NR_132748) chr5: 160467740-160467957(score=808) Regulation SRX682382 level 1 Blood H.sapiens
GABPA hsa-mir-146a chr5: 160468251(NR_132748) chr5: 160467793-160467915(score=303) Regulation SRX326887 level 1 Blood H.sapiens
GATA2 hsa-mir-146a chr5: 160468251(NR_132748) chr5: 160467647-160468114(score=1000) Regulation SRX2574235 level 1 Blood H.sapiens
GATA2 hsa-mir-146a chr5: 160468251(NR_132748) chr5: 160467692-160468010(score=1000) Regulation SRX2574236 level 1 Blood H.sapiens

GATA2 hsa-mir-146a chr5: 160468251(NR_132748) chr5: 160463770-160464056(score=642) Regulation SRX150427 level 1 Cardiovascular H.sapiens
GATA3 hsa-mir-146a chr5: 160468251(NR_132748) chr5: 160467708-160467887(score=544) Regulation SRX1492213 level 1 Blood H.sapiens
GATA3 hsa-mir-146a chr5: 160468251(NR_132748) chr5: 160467708-160467887(score=545) Regulation SRX1041802 level 1 Blood H.sapiens
GATA3 hsa-mir-146a chr5: 160468251(NR_132748) chr5: 160467710-160467878(score=464) Regulation SRX158108 level 1 Blood H.sapiens
GATA3 hsa-mir-146a chr5: 160468251(NR_132748) chr5: 160467742-160467895(score=585) Regulation SRX092315 level 1 Blood H.sapiens
GATA3 hsa-mir-146a chr5: 160468251(NR_132748) chr5: 160467746-160467953(score=452) Regulation SRX092314 level 1 Blood H.sapiens
GATA3 hsa-mir-146a chr5: 160468251(NR_132748) chr5: 160463758-160464139(score=1000) Regulation SRX190194 level 1 Neural H.sapiens
GATA4 hsa-mir-146a chr5: 160468251(NR_132748) chr5: 160463773-160464029(score=1000) Regulation SRX367641 level 1 Digestive tract H.sapiens
GATA6 hsa-mir-146a chr5: 160468251(NR_132748) chr5: 160466971-160467115(score=646) Regulation SRX367638 level 1 Digestive tract H.sapiens
GATA6 hsa-mir-146a chr5: 160468251(NR_132748) chr5: 160467696-160467922(score=1000) Regulation SRX288274 level 1 Pancreas H.sapiens
HDAC1 hsa-mir-146a chr5: 160468251(NR_132748) chr5: 160467581-160467834(score=427) Regulation SRX2140207 level 1 Blood H.sapiens
HDAC1 hsa-mir-146a chr5: 160468251(NR_132748) chr5: 160467785-160467997(score=355) Regulation SRX1514285 level 1 Blood H.sapiens
HDAC1 hsa-mir-146a n/a n/a Repression 26224317 literature n/a H.sapiens
HDAC2 hsa-mir-146a chr5: 160468251(NR_132748) chr5: 160467770-160467951(score=397) Regulation SRX1514286 level 1 Blood H.sapiens
HEY1 hsa-mir-146a chr5: 160468251(NR_132748) chr5: 160467525-160467634(score=259) Regulation SRX100493 level 1 Liver H.sapiens
HEY1 hsa-mir-146a chr5: 160468251(NR_132748) chr5: 160467754-160468007(score=874) Regulation SRX100493 level 1 Liver H.sapiens
HIF1A hsa-mir-146a n/a n/a Activation(feedback) 26045293 literature n/a H.sapiens
HIF1A hsa-mir-146a n/a n/a Activation(feedback) 27783707 literature n/a H.sapiens
HNF1B hsa-mir-146a chr5: 160468251(NR_132748) chr5: 160467512-160468021(score=883) Regulation SRX825396 level 1 Adipocyte H.sapiens
HOXD10 hsa-mir-146a n/a n/a Repression(feedback) 25010866 literature n/a H.sapiens
HSF1 hsa-mir-146a chr5: 160468251(NR_132748) chr5: 160467788-160468081(score=730) Regulation SRX262143 level 1 Blood H.sapiens
HSF1 hsa-mir-146a chr5: 160468251(NR_132748) chr5: 160467922-160468027(score=332) Regulation SRX690823 level 1 Bone H.sapiens

HSF1 hsa-mir-146a chr5: 160468251(NR_132748) chr5: 160467776-160468113(score=843) Regulation SRX767115 level 1 Epidermis H.sapiens
IKZF1 hsa-mir-146a chr5: 160468251(NR_132748) chr5: 160467751-160468412(score=560) Regulation SRX150521 level 1 Blood H.sapiens
IL1B hsa-mir-146a n/a n/a Activation 19333945 literature n/a H.sapiens
IRF1 hsa-mir-146a chr5: 160468251(NR_132748) chr5: 160467631-160468099(score=345) Regulation SRX825397 level 1 Adipocyte H.sapiens
JMJD1C hsa-mir-146a chr5: 160468251(NR_132748) chr5: 160467695-160467976(score=1000) Regulation SRX763970 level 1 Blood H.sapiens
JMJD1C hsa-mir-146a chr5: 160468251(NR_132748) chr5: 160467696-160467928(score=1000) Regulation SRX1304576 level 1 Blood H.sapiens
JMJD1C hsa-mir-146a chr5: 160468251(NR_132748) chr5: 160467725-160467918(score=456) Regulation SRX1304579 level 1 Blood H.sapiens
JUN hsa-mir-146a chr5: 160468251(NR_132748) chr5: 160463858-160464091(score=540) Regulation(feedback) SRX883584 level 1 Breast H.sapiens
JUN hsa-mir-146a chr5: 160468251(NR_132748) chr5: 160463918-160464066(score=443) Regulation(feedback) SRX150358 level 1 Cardiovascular H.sapiens
JUN hsa-mir-146a chr5: 160468251(NR_132748) chr5: 160463881-160464054(score=520) Regulation(feedback) SRX150421 level 1 Uterus H.sapiens
JUN hsa-mir-146a n/a n/a Activation(feedback) 26718614 literature n/a H.sapiens
JUND hsa-mir-146a chr5: 160468251(NR_132748) chr5: 160463811-160464112(score=1000) Regulation SRX190357 level 1 Neural H.sapiens
JUND hsa-mir-146a chr5: 160468251(NR_132748) chr5: 160463898-160464059(score=445) Regulation SRX186637 level 1 Neural H.sapiens
JUND hsa-mir-146a chr5: 160468251(NR_132748) chr5: 160463895-160464095(score=606) Regulation SRX150408 level 1 Uterus H.sapiens
KDM2B hsa-mir-146a chr5: 160468251(NR_132748) chr5: 160467736-160468186(score=1000) Regulation SRX1873447 level 1 Blood H.sapiens
KMT2A hsa-mir-146a n/a n/a Activation 24699235 literature n/a H.sapiens
KMT2D hsa-mir-146a chr5: 160468251(NR_132748) chr5: 160467701-160468212(score=580) Regulation SRX971780 level 1 Blood H.sapiens
LMO2 hsa-mir-146a chr5: 160468251(NR_132748) chr5: 160467679-160468010(score=1000) Regulation SRX682385 level 1 Blood H.sapiens
LMO2 hsa-mir-146a chr5: 160468251(NR_132748) chr5: 160467716-160468057(score=1000) Regulation SRX698186 level 1 Blood H.sapiens
LMO3 hsa-mir-146a chr5: 160468251(NR_132748) chr5: 160467721-160468031(score=1000) Regulation SRX698187 level 1 Blood H.sapiens
LYL1 hsa-mir-146a chr5: 160468251(NR_132748) chr5: 160467656-160468124(score=1000) Regulation SRX1514281 level 1 Blood H.sapiens
LYL1 hsa-mir-146a chr5: 160468251(NR_132748) chr5: 160467710-160467983(score=1000) Regulation SRX1304574 level 1 Blood H.sapiens

LYL1 hsa-mir-146a chr5: 160468251(NR_132748) chr5: 160467718-160467924(score=822) Regulation SRX682387 level 1 Blood H.sapiens
LYL1 hsa-mir-146a chr5: 160468251(NR_132748) chr5: 160467745-160467937(score=718) Regulation SRX1304580 level 1 Blood H.sapiens
LYL1 hsa-mir-146a chr5: 160468251(NR_132748) chr5: 160467753-160467914(score=545) Regulation SRX1304577 level 1 Blood H.sapiens
MAFK hsa-mir-146a chr5: 160468251(NR_132748) chr5: 160463358-160463492(score=430) Regulation SRX150391 level 1 Blood H.sapiens
MAFK hsa-mir-146a chr5: 160468251(NR_132748) chr5: 160463350-160463506(score=726) Regulation SRX150385 level 1 Liver H.sapiens
MAFK hsa-mir-146a chr5: 160468251(NR_132748) chr5: 160463353-160463501(score=553) Regulation SRX150483 level 1 Lung H.sapiens
MAX hsa-mir-146a chr5: 160468251(NR_132748) chr5: 160467840-160468165(score=498) Regulation SRX129084 level 1 Blood H.sapiens
MAX hsa-mir-146a chr5: 160468251(NR_132748) chr5: 160467859-160468056(score=851) Regulation SRX150723 level 1 Blood H.sapiens
MAX hsa-mir-146a chr5: 160468251(NR_132748) chr5: 160467549-160467728(score=575) Regulation SRX190321 level 1 Liver H.sapiens
MAX hsa-mir-146a chr5: 160468251(NR_132748) chr5: 160467839-160468080(score=585) Regulation SRX190321 level 1 Liver H.sapiens
MED1 hsa-mir-146a chr5: 160468252 chr5: 160468075-160468340(score=307) Regulation SRX203396 level 2 Blood H.sapiens
MED1 hsa-mir-146a chr5: 160468251(NR_132748) chr5: 160467598-160468216(score=1000) Regulation SRX1873451 level 1 Blood H.sapiens
MED1 hsa-mir-146a chr5: 160468251(NR_132748) chr5: 160467654-160467925(score=421) Regulation SRX327756 level 1 Blood H.sapiens
MED1 hsa-mir-146a chr5: 160468251(NR_132748) chr5: 160467658-160468389(score=651) Regulation SRX244178 level 1 Blood H.sapiens
MED1 hsa-mir-146a chr5: 160468251(NR_132748) chr5: 160467669-160468388(score=653) Regulation SRX204906 level 1 Blood H.sapiens
MED1 hsa-mir-146a chr5: 160468251(NR_132748) chr5: 160467675-160468081(score=563) Regulation SRX327755 level 1 Blood H.sapiens
MED1 hsa-mir-146a chr5: 160468251(NR_132748) chr5: 160467737-160468368(score=402) Regulation SRX327757 level 1 Blood H.sapiens
MED1 hsa-mir-146a chr5: 160468251(NR_132748) chr5: 160468113-160468353(score=368) Regulation SRX327756 level 1 Blood H.sapiens
MED1 hsa-mir-146a chr5: 160468251(NR_132748) chr5: 160468175-160468386(score=377) Regulation SRX327755 level 1 Blood H.sapiens
MED1 hsa-mir-146a chr5: 160468251(NR_132748) chr5: 160467722-160468061(score=267) Regulation SRX1013331 level 1 Liver H.sapiens
MED1 hsa-mir-146a chr5: 160468251(NR_132748) chr5: 160463841-160464030(score=423) Regulation SRX129090 level 1 Neural H.sapiens
MED12 hsa-mir-146a chr5: 160468251(NR_132748) chr5: 160467708-160467905(score=633) Regulation SRX1477666 level 1 Blood H.sapiens
MEF2A hsa-mir-146a chr5: 160468251(NR_132748) chr5: 160467642-160467830(score=704) Regulation SRX100556 level 1 Blood H.sapiens
MEF2C hsa-mir-146a chr5: 160468251(NR_132748) chr5: 160467679-160467823(score=369) Regulation SRX100465 level 1 Blood H.sapiens
MITF hsa-mir-146a chr5: 160468251(NR_132748) chr5: 160467729-160467971(score=404) Regulation SRX346924 level 1 Digestive tract H.sapiens

MITF hsa-mir-146a chr5: 160468251(NR_132748) chr5: 160467748-160468047(score=608) Regulation SRX346921 level 1 Epidermis H.sapiens
MITF hsa-mir-146a n/a n/a Regulation 19056895 literature n/a H.sapiens
MTA3 hsa-mir-146a chr5: 160468252 chr5: 160468178-160468322(score=309) Regulation SRX190185 level 2 Blood H.sapiens
MXI1 hsa-mir-146a chr5: 160468251(NR_132748) chr5: 160467592-160467770(score=408) Regulation SRX150510 level 1 Blood H.sapiens
MYB hsa-mir-146a chr5: 160468251(NR_132748) chr5: 160467574-160467938(score=1000) Regulation SRX725584 level 1 Blood H.sapiens
MYB hsa-mir-146a chr5: 160468251(NR_132748) chr5: 160467608-160467924(score=1000) Regulation SRX658610 level 1 Blood H.sapiens
MYB hsa-mir-146a chr5: 160468251(NR_132748) chr5: 160467621-160467924(score=1000) Regulation SRX725582 level 1 Blood H.sapiens
MYB hsa-mir-146a chr5: 160468251(NR_132748) chr5: 160467628-160467914(score=1000) Regulation SRX658611 level 1 Blood H.sapiens
MYB hsa-mir-146a chr5: 160468251(NR_132748) chr5: 160467651-160467890(score=922) Regulation SRX725578 level 1 Blood H.sapiens
MYC hsa-mir-146a chr5: 160468252 chr5: 160468189-160468346(score=404) Regulation SRX082165 level 2 Blood H.sapiens
MYC hsa-mir-146a chr5: 160468251(NR_132748) chr5: 160467598-160468153(score=634) Regulation SRX129116 level 1 Blood H.sapiens
MYC hsa-mir-146a chr5: 160468251(NR_132748) chr5: 160467638-160468206(score=690) Regulation SRX1497394 level 1 Blood H.sapiens
MYC hsa-mir-146a chr5: 160468251(NR_132748) chr5: 160467826-160468022(score=661) Regulation SRX150722 level 1 Blood H.sapiens
MYC hsa-mir-146a chr5: 160468251(NR_132748) chr5: 160467877-160468122(score=392) Regulation SRX1497384 level 1 Blood H.sapiens
MYC hsa-mir-146a n/a n/a Repression 18066065 literature n/a H.sapiens
MYC hsa-mir-146a n/a n/a Activation 24550252 literature n/a H.sapiens
NCOR1 hsa-mir-146a chr5: 160468251(NR_132748) chr5: 160467700-160467945(score=977) Regulation SRX747573 level 1 Blood H.sapiens
NCOR2 hsa-mir-146a chr5: 160468251(NR_132748) chr5: 160467743-160467911(score=567) Regulation SRX084601 level 1 Blood H.sapiens
NFE2L2 hsa-mir-146a n/a n/a Activation 25623956 literature n/a H.sapiens
NFIC hsa-mir-146a chr5: 160468251(NR_132748) chr5: 160467651-160468249(score=915) Regulation SRX190337 level 1 Blood H.sapiens
NFIC hsa-mir-146a chr5: 160468251(NR_132748) chr5: 160467553-160467774(score=264) Regulation SRX190197 level 1 Liver H.sapiens
NFIC hsa-mir-146a chr5: 160468251(NR_132748) chr5: 160463820-160464116(score=1000) Regulation SRX190336 level 1 Neural H.sapiens
NFKB1 hsa-mir-146a chr5: 160468252 chr5: 160468052-160468189(score=602) Regulation(feedback) SRX790970 level 2 Blood H.sapiens
NFKB1 hsa-mir-146a chr5: 160468252 chr5: 160468075-160468161(score=484) Regulation(feedback) SRX790969 level 2 Blood H.sapiens

NFKB1 hsa-mir-146a chr5: 160468251(NR_132748) chr5: 160467733-160467946(score=1000) Regulation(feedback) SRX790970 level 1 Blood H.sapiens
NFKB1 hsa-mir-146a chr5: 160468251(NR_132748) chr5: 160467760-160467875(score=847) Regulation(feedback) SRX790969 level 1 Blood H.sapiens
NFKB1 hsa-mir-146a n/a n/a Activation(feedback) 19021527 literature n/a H.sapiens
NFKB1 hsa-mir-146a n/a n/a Activation(feedback) 20061417 literature n/a H.sapiens
NFKB1 hsa-mir-146a n/a n/a Regulation(feedback) 22927641 literature n/a H.sapiens
NFKB1 hsa-mir-146a n/a n/a Activation(feedback) 24107356 literature n/a H.sapiens
NFKB1 hsa-mir-146a n/a n/a Activation(feedback) 24985472 literature n/a H.sapiens
NFKB2 hsa-mir-146a chr5: 160468252 chr5: 160468065-160468197(score=679) Regulation SRX790972 level 2 Blood H.sapiens
NFKB2 hsa-mir-146a chr5: 160468252 chr5: 160468104-160468191(score=356) Regulation SRX790971 level 2 Blood H.sapiens
NFKB2 hsa-mir-146a chr5: 160468251(NR_132748) chr5: 160467732-160467942(score=1000) Regulation SRX790972 level 1 Blood H.sapiens
NFKB2 hsa-mir-146a chr5: 160468251(NR_132748) chr5: 160467751-160467913(score=653) Regulation SRX790971 level 1 Blood H.sapiens
NOTCH1 hsa-mir-146a chr5: 160468251(NR_132748) chr5: 160467683-160468037(score=463) Regulation(feedback) SRX159138 level 1 Blood H.sapiens
NR3C1 hsa-mir-146a chr5: 160468251(NR_132748) chr5: 160463822-160464056(score=431) Regulation SRX1672124 level 1 Lung H.sapiens
NR3C1 hsa-mir-146a chr5: 160468251(NR_132748) chr5: 160463848-160464054(score=378) Regulation SRX1672123 level 1 Lung H.sapiens
NR3C1 hsa-mir-146a chr5: 160468251(NR_132748) chr5: 160463861-160464082(score=472) Regulation SRX2609659 level 1 Lung H.sapiens
NR4A1 hsa-mir-146a chr5: 160468251(NR_132748) chr5: 160467786-160467943(score=387) Regulation SRX1653204 level 1 Blood H.sapiens
NR4A1 hsa-mir-146a chr5: 160468251(NR_132748) chr5: 160467814-160467930(score=388) Regulation SRX1653203 level 1 Blood H.sapiens
PAX5 hsa-mir-146a chr5: 160468252 chr5: 160468084-160468268(score=411) Regulation SRX100407 level 2 Blood H.sapiens
PAX5 hsa-mir-146a chr5: 160468252 chr5: 160468143-160468250(score=367) Regulation SRX100436 level 2 Blood H.sapiens
PAX5 hsa-mir-146a chr5: 160468251(NR_132748) chr5: 160467718-160467922(score=560) Regulation SRX100407 level 1 Blood H.sapiens
PBX1 hsa-mir-146a chr5: 160468251(NR_132748) chr5: 160467580-160468133(score=1000) Regulation SRX2043232 level 1 Blood H.sapiens
PBX1 hsa-mir-146a chr5: 160468251(NR_132748) chr5: 160467614-160468030(score=1000) Regulation SRX656349 level 1 Blood H.sapiens
PBX3 hsa-mir-146a chr5: 160468251(NR_132748) chr5: 160463775-160464145(score=1000) Regulation SRX190343 level 1 Neural H.sapiens
PLZF hsa-mir-146a n/a n/a Repression 18568019 literature n/a H.sapiens

PML hsa-mir-146a chr5: 160468251(NR_132748) chr5: 160468153-
160468376(score=531) Regulation SRX190227 level 1 Blood H.sapiens
POU2F2 hsa-mir-146a chr5: 160468251(NR_132748) chr5: 160467784-
160467900(score=322) Regulation SRX100442 level 1 Blood H.sapiens
POU2F2 hsa-mir-146a chr5: 160468251(NR_132748) chr5: 160467949-
160468368(score=1000) Regulation SRX100442 level 1 Blood H.sapiens
POU2F2 hsa-mir-146a chr5: 160468251(NR_132748) chr5: 160468052-
160468374(score=1000) Regulation SRX100579 level 1 Blood H.sapiens
RARA hsa-mir-146a chr5: 160468251(NR_132748) chr5: 160467732-
160468050(score=956) Regulation SRX682379 level 1 Blood H.sapiens
RARA hsa-mir-146a chr5: 160468251(NR_132748) chr5: 160467768-
160467961(score=384) Regulation SRX014782 level 1 Blood H.sapiens
RBBP5 hsa-mir-146a n/a n/a Activation 24699235 literature n/a
H.sapiens
RCOR1 hsa-mir-146a chr5: 160468251(NR_132748) chr5: 160463863-
160463965(score=354) Regulation SRX150657 level 1 Uterus
H.sapiens
RELA hsa-mir-146a chr5: 160468251(NR_132748) chr5: 160467712-
160468258(score=689) Regulation SRX813775 level 1 Adipocyte
H.sapiens
RELA hsa-mir-146a chr5: 160468252 chr5: 160468020-
160468307(score=980) Regulation SRX017913 level 2 Blood H.sapiens
RELA hsa-mir-146a chr5: 160468252 chr5: 160468021-
160468307(score=1000) Regulation SRX017912 level 2 Blood H.sapiens
RELA hsa-mir-146a chr5: 160468252 chr5: 160468030-
160468234(score=485) Regulation SRX017891 level 2 Blood H.sapiens
RELA hsa-mir-146a chr5: 160468252 chr5: 160468034-
160468273(score=486) Regulation SRX017872 level 2 Blood H.sapiens
RELA hsa-mir-146a chr5: 160468252 chr5: 160468035-
160468235(score=1000) Regulation SRX1460847 level 2 Blood H.sapiens
RELA hsa-mir-146a chr5: 160468252 chr5: 160468036-
160468281(score=862) Regulation SRX150362 level 2 Blood H.sapiens
RELA hsa-mir-146a chr5: 160468252 chr5: 160468039-
160468299(score=1000) Regulation SRX150557 level 2 Blood H.sapiens
RELA hsa-mir-146a chr5: 160468252 chr5: 160468045-
160468288(score=874) Regulation SRX150608 level 2 Blood H.sapiens
RELA hsa-mir-146a chr5: 160468252 chr5: 160468046-
160468301(score=1000) Regulation SRX150610 level 2 Blood H.sapiens
RELA hsa-mir-146a chr5: 160468252 chr5: 160468056-
160468263(score=571) Regulation SRX017906 level 2 Blood H.sapiens
RELA hsa-mir-146a chr5: 160468252 chr5: 160468056-
160468268(score=943) Regulation SRX150606 level 2 Blood H.sapiens
RELA hsa-mir-146a chr5: 160468252 chr5: 160468058-
160468272(score=548) Regulation SRX017870 level 2 Blood H.sapiens
RELA hsa-mir-146a chr5: 160468252 chr5: 160468061-
160468254(score=961) Regulation SRX017895 level 2 Blood H.sapiens
RELA hsa-mir-146a chr5: 160468252 chr5: 160468068-
160468235(score=633) Regulation SRX017860 level 2 Blood H.sapiens
RELA hsa-mir-146a chr5: 160468252 chr5: 160468069-
160468244(score=488) Regulation SRX017897 level 2 Blood H.sapiens
RELA hsa-mir-146a chr5: 160468252 chr5: 160468075-
160468261(score=436) Regulation SRX017867 level 2 Blood H.sapiens

RELA hsa-mir-146a chr5: 160468252 chr5: 160468076-
160468247(score=445) Regulation SRX017908 level 2 Blood H.sapiens
RELA hsa-mir-146a chr5: 160468252 chr5: 160468083-
160468244(score=540) Regulation SRX150365 level 2 Blood H.sapiens
RELA hsa-mir-146a chr5: 160468252 chr5: 160468092-
160468235(score=433) Regulation SRX017878 level 2 Blood H.sapiens
RELA hsa-mir-146a chr5: 160468252 chr5: 160468096-
160468238(score=422) Regulation SRX017884 level 2 Blood H.sapiens
RELA hsa-mir-146a chr5: 160468252 chr5: 160468101-
160468245(score=344) Regulation SRX017879 level 2 Blood H.sapiens
RELA hsa-mir-146a chr5: 160468252 chr5: 160468110-
160468240(score=418) Regulation SRX017907 level 2 Blood H.sapiens
RELA hsa-mir-146a chr5: 160468252 chr5: 160468120-
160468221(score=407) Regulation SRX150361 level 2 Blood H.sapiens
RELA hsa-mir-146a chr5: 160468251(NR_132748) chr5: 160467642-
160468362(score=1000) Regulation SRX150605 level 1 Blood H.sapiens
RELA hsa-mir-146a chr5: 160468251(NR_132748) chr5: 160467648-
160468301(score=1000) Regulation SRX150353 level 1 Blood H.sapiens
RELA hsa-mir-146a chr5: 160468251(NR_132748) chr5: 160467650-
160467986(score=802) Regulation SRX150557 level 1 Blood H.sapiens
RELA hsa-mir-146a chr5: 160468251(NR_132748) chr5: 160467677-
160467934(score=562) Regulation SRX017906 level 1 Blood H.sapiens
RELA hsa-mir-146a chr5: 160468251(NR_132748) chr5: 160467682-
160467959(score=965) Regulation SRX017912 level 1 Blood H.sapiens
RELA hsa-mir-146a chr5: 160468251(NR_132748) chr5: 160467683-
160468279(score=1000) Regulation SRX150359 level 1 Blood H.sapiens
RELA hsa-mir-146a chr5: 160468251(NR_132748) chr5: 160467688-
160467951(score=645) Regulation SRX017913 level 1 Blood H.sapiens
RELA hsa-mir-146a chr5: 160468251(NR_132748) chr5: 160467694-
160467915(score=678) Regulation SRX150610 level 1 Blood H.sapiens
RELA hsa-mir-146a chr5: 160468251(NR_132748) chr5: 160467700-
160468280(score=1000) Regulation SRX017871 level 1 Blood H.sapiens
RELA hsa-mir-146a chr5: 160468251(NR_132748) chr5: 160467701-
160467981(score=653) Regulation SRX150606 level 1 Blood H.sapiens
RELA hsa-mir-146a chr5: 160468251(NR_132748) chr5: 160467716-
160467927(score=546) Regulation SRX150362 level 1 Blood H.sapiens
RELA hsa-mir-146a chr5: 160468251(NR_132748) chr5: 160467719-
160467915(score=498) Regulation SRX017891 level 1 Blood H.sapiens
RELA hsa-mir-146a chr5: 160468251(NR_132748) chr5: 160467721-
160468250(score=757) Regulation SRX017859 level 1 Blood H.sapiens
RELA hsa-mir-146a chr5: 160468251(NR_132748) chr5: 160467722-
160467923(score=557) Regulation SRX150608 level 1 Blood H.sapiens
RELA hsa-mir-146a chr5: 160468251(NR_132748) chr5: 160467732-
160467916(score=509) Regulation SRX150365 level 1 Blood H.sapiens
RELA hsa-mir-146a chr5: 160468251(NR_132748) chr5: 160467767-
160467889(score=463) Regulation SRX017895 level 1 Blood H.sapiens
RELA hsa-mir-146a chr5: 160468251(NR_132748) chr5: 160467778-
160467918(score=604) Regulation SRX1460847 level 1 Blood H.sapiens
RELA hsa-mir-146a chr5: 160468251(NR_132748) chr5: 160467796-
160468294(score=484) Regulation SRX017911 level 1 Blood H.sapiens

RELA hsa-mir-146a chr5: 160468252 chr5: 160468047-160468223(score=601) Regulation SRX2355085 level 2 Cardiovascular H.sapiens
RELA hsa-mir-146a chr5: 160468252 chr5: 160468049-160468222(score=967) Regulation SRX2355081 level 2 Cardiovascular H.sapiens
RELA hsa-mir-146a chr5: 160468252 chr5: 160468053-160468178(score=329) Regulation SRX112016 level 2 Cardiovascular H.sapiens
RELA hsa-mir-146a chr5: 160468251(NR_132748) chr5: 160467702-160468242(score=820) Regulation SRX425263 level 1 Cardiovascular H.sapiens
RELA hsa-mir-146a chr5: 160468251(NR_132748) chr5: 160467706-160468171(score=1000) Regulation SRX294971 level 1 Cardiovascular H.sapiens
RELA hsa-mir-146a chr5: 160468251(NR_132748) chr5: 160467717-160468253(score=832) Regulation SRX425264 level 1 Cardiovascular H.sapiens
RELA hsa-mir-146a chr5: 160468251(NR_132748) chr5: 160467762-160468181(score=437) Regulation SRX2355083 level 1 Cardiovascular H.sapiens
RELA hsa-mir-146a chr5: 160468251(NR_132748) chr5: 160467764-160468258(score=704) Regulation SRX2355079 level 1 Cardiovascular H.sapiens
RELA hsa-mir-146a chr5: 160468251(NR_132748) chr5: 160467784-160467925(score=421) Regulation SRX112016 level 1 Cardiovascular H.sapiens
RELA hsa-mir-146a chr5: 160468251(NR_132748) chr5: 160467628-160467852(score=485) Regulation SRX3102571 level 1 Digestive tract H.sapiens
RELA hsa-mir-146a chr5: 160468251(NR_132748) chr5: 160467672-160468381(score=1000) Regulation SRX2055843 level 1 Kidney H.sapiens
RELA hsa-mir-146a chr5: 160468251(NR_132748) chr5: 160467756-160468213(score=597) Regulation SRX2270129 level 1 Liver H.sapiens
RELA hsa-mir-146a chr5: 160468251(NR_132748) chr5: 160467743-160468170(score=341) Regulation SRX1672133 level 1 Lung H.sapiens
RELA hsa-mir-146a chr5: 160468252 chr5: 160468049-160468208(score=816) Regulation SRX378805 level 2 Uterus H.sapiens
RELA hsa-mir-146a chr5: 160468251(NR_132748) chr5: 160467781-160467892(score=362) Regulation SRX378805 level 1 Uterus H.sapiens
RELA hsa-mir-146a n/a n/a Activation 25623956 literature n/a H.sapiens
RELA hsa-mir-146a n/a n/a Activation 25757558 literature n/a H.sapiens
RELA hsa-mir-146a n/a n/a Activation 26224317 literature n/a H.sapiens
RELA hsa-mir-146a n/a n/a Activation 26718614 literature n/a H.sapiens

RUNX1 hsa-mir-146a chr5: 160468251(NR_132748) chr5: 160467626-
160467920(score=699) Regulation SRX259622 level 1 Blood H.sapiens
RUNX1 hsa-mir-146a chr5: 160468251(NR_132748) chr5: 160467628-
160467945(score=816) Regulation SRX259621 level 1 Blood H.sapiens
RUNX1 hsa-mir-146a chr5: 160468251(NR_132748) chr5: 160467631-
160468000(score=1000) Regulation SRX202967 level 1 Blood H.sapiens
RUNX1 hsa-mir-146a chr5: 160468251(NR_132748) chr5: 160467637-
160468022(score=542) Regulation SRX259612 level 1 Blood H.sapiens
RUNX1 hsa-mir-146a chr5: 160468251(NR_132748) chr5: 160467640-
160468100(score=1000) Regulation SRX1803059 level 1 Blood H.sapiens
RUNX1 hsa-mir-146a chr5: 160468251(NR_132748) chr5: 160467653-
160468009(score=1000) Regulation SRX682384 level 1 Blood H.sapiens
RUNX1 hsa-mir-146a chr5: 160468251(NR_132748) chr5: 160467657-
160468206(score=1000) Regulation SRX062339 level 1 Blood H.sapiens
RUNX1 hsa-mir-146a chr5: 160468251(NR_132748) chr5: 160467657-
160468206(score=1000) Regulation SRX838202 level 1 Blood H.sapiens
RUNX1 hsa-mir-146a chr5: 160468251(NR_132748) chr5: 160467687-
160468018(score=1000) Regulation SRX063913 level 1 Blood H.sapiens
RUNX1 hsa-mir-146a chr5: 160468251(NR_132748) chr5: 160467688-
160467943(score=877) Regulation SRX747571 level 1 Blood H.sapiens
RUNX1 hsa-mir-146a chr5: 160468251(NR_132748) chr5: 160467709-
160467882(score=454) Regulation SRX1041800 level 1 Blood H.sapiens
RUNX1 hsa-mir-146a chr5: 160468251(NR_132748) chr5: 160467709-
160467882(score=456) Regulation SRX1492212 level 1 Blood H.sapiens
RUNX1 hsa-mir-146a chr5: 160468251(NR_132748) chr5: 160467711-
160468073(score=1000) Regulation SRX1514291 level 1 Blood H.sapiens
RUNX1 hsa-mir-146a chr5: 160468251(NR_132748) chr5: 160467716-
160467987(score=878) Regulation SRX265222 level 1 Blood H.sapiens
RUNX1 hsa-mir-146a chr5: 160468251(NR_132748) chr5: 160467718-
160468091(score=1000) Regulation SRX669872 level 1 Blood H.sapiens
RUNX1 hsa-mir-146a chr5: 160468251(NR_132748) chr5: 160467718-
160468092(score=1000) Regulation SRX1036365 level 1 Blood H.sapiens
RUNX1 hsa-mir-146a chr5: 160468251(NR_132748) chr5: 160467719-
160467883(score=325) Regulation SRX952434 level 1 Blood H.sapiens
RUNX1 hsa-mir-146a chr5: 160468251(NR_132748) chr5: 160467730-
160467910(score=327) Regulation SRX259611 level 1 Blood H.sapiens
RUNX1 hsa-mir-146a chr5: 160468251(NR_132748) chr5: 160467753-
160467934(score=682) Regulation SRX858324 level 1 Blood H.sapiens
RUNX1 hsa-mir-146a chr5: 160468251(NR_132748) chr5: 160467756-
160467986(score=311) Regulation SRX1036364 level 1 Blood H.sapiens
RUNX1 hsa-mir-146a chr5: 160468251(NR_132748) chr5: 160467774-
160467949(score=360) Regulation SRX112545 level 1 Blood H.sapiens
RUNX1 hsa-mir-146a chr5: 160468251(NR_132748) chr5: 160467782-
160467931(score=357) Regulation SRX112544 level 1 Blood H.sapiens
RUNX1 hsa-mir-146a chr5: 160468251(NR_132748) chr5: 160467784-
160467973(score=690) Regulation SRX858323 level 1 Blood H.sapiens
RUNX1T1 hsa-mir-146a chr5: 160468251(NR_132748) chr5: 160467666-
160467965(score=1000) Regulation SRX747572 level 1 Blood H.sapiens
RUNX1T1 hsa-mir-146a chr5: 160468251(NR_132748) chr5: 160467686-
160468059(score=1000) Regulation SRX1514278 level 1 Blood H.sapiens
RUNX1T1 hsa-mir-146a chr5: 160468251(NR_132748) chr5: 160467751-
160467955(score=743) Regulation SRX236112 level 1 Blood H.sapiens

RUNX3 hsa-mir-146a chr5: 160468252 chr5: 160468064-160468344(score=730) Regulation SRX190349 level 2 Blood H.sapiens
RUNX3 hsa-mir-146a chr5: 160468251(NR_132748) chr5: 160467629-160467994(score=1000) Regulation SRX190349 level 1 Blood H.sapiens
RXRA hsa-mir-146a chr5: 160468251(NR_132748) chr5: 160463840-160464019(score=350) Regulation SRX190223 level 1 Neural H.sapiens
SFPQ hsa-mir-146a chr5: 160468251(NR_132748) chr5: 160467726-160467951(score=407) Regulation SRX593748 level 1 Breast H.sapiens
SMAD1 hsa-mir-146a chr5: 160468251(NR_132748) chr5: 160467681-160467982(score=1000) Regulation SRX097097 level 1 Blood H.sapiens
SMAD1 hsa-mir-146a chr5: 160468251(NR_132748) chr5: 160467757-160467907(score=488) Regulation SRX097073 level 1 Blood H.sapiens
SMARCA4 hsa-mir-146a chr5: 160485352 chr5: 160485567-160485729(score=369) Regulation SRX027488 level 1 Pluripotent stem cell H.sapiens
SP1 hsa-mir-146a chr5: 160468252 chr5: 160468072-160468260(score=770) Regulation SRX100408 level 2 Blood H.sapiens
SP1 hsa-mir-146a chr5: 160468251(NR_132748) chr5: 160467746-160467920(score=390) Regulation SRX100408 level 1 Blood H.sapiens
SP1 hsa-mir-146a chr5: 160468251(NR_132748) chr5: 160467565-160467734(score=366) Regulation SRX100552 level 1 Liver H.sapiens
SPI1 hsa-mir-146a chr5: 160468252 chr5: 160468006-160468216(score=414) Regulation SRX627428 level 2 Blood H.sapiens
SPI1 hsa-mir-146a chr5: 160468252 chr5: 160468036-160468200(score=630) Regulation SRX1023791 level 2 Blood H.sapiens
SPI1 hsa-mir-146a chr5: 160468252 chr5: 160468040-160468300(score=599) Regulation SRX2770854 level 2 Blood H.sapiens
SPI1 hsa-mir-146a chr5: 160468252 chr5: 160468043-160468193(score=450) Regulation SRX100576 level 2 Blood H.sapiens
SPI1 hsa-mir-146a chr5: 160468252 chr5: 160468068-160468216(score=302) Regulation SRX1048461 level 2 Blood H.sapiens
SPI1 hsa-mir-146a chr5: 160468252 chr5: 160468069-160468238(score=483) Regulation SRX100443 level 2 Blood H.sapiens
SPI1 hsa-mir-146a chr5: 160468251(NR_132748) chr5: 160467721-160468367(score=1000) Regulation SRX2770855 level 1 Blood H.sapiens
SPI1 hsa-mir-146a chr5: 160468251(NR_132748) chr5: 160467751-160468314(score=1000) Regulation SRX1023792 level 1 Blood H.sapiens
SPI1 hsa-mir-146a chr5: 160468251(NR_132748) chr5: 160467752-160467972(score=491) Regulation SRX698189 level 1 Blood H.sapiens
SPI1 hsa-mir-146a chr5: 160468251(NR_132748) chr5: 160467754-160467918(score=469) Regulation SRX794057 level 1 Blood H.sapiens
SPI1 hsa-mir-146a chr5: 160468251(NR_132748) chr5: 160467754-160468288(score=968) Regulation SRX2770857 level 1 Blood H.sapiens
SPI1 hsa-mir-146a chr5: 160468251(NR_132748) chr5: 160467757-160467934(score=557) Regulation SRX698188 level 1 Blood H.sapiens
SPI1 hsa-mir-146a chr5: 160468251(NR_132748) chr5: 160467773-160468290(score=1000) Regulation SRX1023793 level 1 Blood H.sapiens
SPI1 hsa-mir-146a chr5: 160468251(NR_132748) chr5: 160467774-160468283(score=1000) Regulation SRX1023790 level 1 Blood H.sapiens

SPI1 hsa-mir-146a n/a n/a Activation 20375304 literature n/a H.sapiens
SPI1 hsa-mir-146a n/a n/a Activation 21730352 literature n/a H.sapiens
SPI1 hsa-mir-146a n/a n/a Activation 28399410 literature n/a H.sapiens
STAT1 hsa-mir-146a chr5: 160468252 chr5: 160468038-160468246(score=569) Regulation(feedback) SRX212648 level 2 Blood H.sapiens
STAT1 hsa-mir-146a chr5: 160468252 chr5: 160468052-160468325(score=548) Regulation(feedback) SRX212649 level 2 Blood H.sapiens
STAT1 hsa-mir-146a chr5: 160468252 chr5: 160468066-160468189(score=356) Regulation(feedback) SRX212651 level 2 Blood H.sapiens
STAT1 hsa-mir-146a chr5: 160468251(NR_132748) chr5: 160467725-160468369(score=1000) Regulation(feedback) SRX212650 level 1 Blood H.sapiens
STAT1 hsa-mir-146a chr5: 160468251(NR_132748) chr5: 160467794-160467908(score=352) Regulation(feedback) SRX212648 level 1 Blood H.sapiens
STAT3 hsa-mir-146a chr5: 160468252 chr5: 160468085-160468291(score=1000) Regulation SRX347424 level 2 Blood H.sapiens
STAT3 hsa-mir-146a chr5: 160468252 chr5: 160468090-160468285(score=1000) Regulation SRX347427 level 2 Blood H.sapiens
STAT3 hsa-mir-146a chr5: 160468252 chr5: 160468139-160468266(score=493) Regulation SRX347425 level 2 Blood H.sapiens
STAT3 hsa-mir-146a chr5: 160468252 chr5: 160468162-160468247(score=264) Regulation SRX347428 level 2 Blood H.sapiens
STAT3 hsa-mir-146a chr5: 160468251(NR_132748) chr5: 160463895-160464079(score=457) Regulation SRX150536 level 1 Breast H.sapiens
STAT3 hsa-mir-146a chr5: 160468251(NR_132748) chr5: 160463867-160464028(score=495) Regulation SRX150356 level 1 Uterus H.sapiens
STAT3 hsa-mir-146a n/a n/a Activation 25607648 literature n/a H.sapiens
STAT5B hsa-mir-146a chr5: 160468251(NR_132748) chr5: 160467731-160467948(score=377) Regulation(feedback) SRX831874 level 1 Blood H.sapiens
TAF1 hsa-mir-146a chr5: 160468252 chr5: 160468189-160468339(score=595) Regulation SRX100534 level 2 Blood H.sapiens
TAF1 hsa-mir-146a chr5: 160468251(NR_132748) chr5: 160468115-160468360(score=752) Regulation SRX100582 level 1 Blood H.sapiens
TAF1 hsa-mir-146a chr5: 160468251(NR_132748) chr5: 160468163-160468360(score=827) Regulation SRX100438 level 1 Blood H.sapiens
TAF1 hsa-mir-146a chr5: 160468251(NR_132748) chr5: 160467837-160468032(score=793) Regulation SRX100412 level 1 Liver H.sapiens
TAL1 hsa-mir-146a chr5: 160468251(NR_132748) chr5: 160467642-160467982(score=1000) Regulation SRX158111 level 1 Blood H.sapiens
TAL1 hsa-mir-146a chr5: 160468251(NR_132748) chr5: 160467685-160467930(score=952) Regulation SRX725589 level 1 Blood H.sapiens

TAL1 hsa-mir-146a chr5: 160468251(NR_132748) chr5: 160467688-160467966(score=1000) Regulation SRX158113 level 1 Blood H.sapiens
TAL1 hsa-mir-146a chr5: 160468251(NR_132748) chr5: 160467689-160467990(score=1000) Regulation SRX682388 level 1 Blood H.sapiens
TAL1 hsa-mir-146a chr5: 160468251(NR_132748) chr5: 160467719-160467931(score=565) Regulation ERX626846 level 1 Blood H.sapiens
TAL1 hsa-mir-146a chr5: 160468251(NR_132748) chr5: 160467723-160467957(score=616) Regulation ERX626834 level 1 Blood H.sapiens
TAL1 hsa-mir-146a chr5: 160468251(NR_132748) chr5: 160467752-160467975(score=437) Regulation SRX1514280 level 1 Blood H.sapiens
TAL1 hsa-mir-146a chr5: 160468251(NR_132748) chr5: 160463730-160463972(score=917) Regulation SRX393518 level 1 Cardiovascular H.sapiens
TAL1 hsa-mir-146a chr5: 160468251(NR_132748) chr5: 160463732-160463971(score=852) Regulation SRX393516 level 1 Cardiovascular H.sapiens
TAL1 hsa-mir-146a chr5: 160468251(NR_132748) chr5: 160467688-160468006(score=1000) Regulation SRX393518 level 1 Cardiovascular H.sapiens
TAL1 hsa-mir-146a chr5: 160468251(NR_132748) chr5: 160467697-160468061(score=1000) Regulation SRX393516 level 1 Cardiovascular H.sapiens
TBL1XR1 hsa-mir-146a chr5: 160468251(NR_132748) chr5: 160467752-160467890(score=314) Regulation SRX150732 level 1 Blood H.sapiens
TBP hsa-mir-146a chr5: 160468251(NR_132748) chr5: 160467600-160467751(score=477) Regulation SRX150357 level 1 Blood H.sapiens
TBP hsa-mir-146a chr5: 160468251(NR_132748) chr5: 160468105-160468391(score=1000) Regulation SRX150357 level 1 Blood H.sapiens
TBP hsa-mir-146a chr5: 160468251(NR_132748) chr5: 160467617-160467849(score=270) Regulation SRX150360 level 1 Liver H.sapiens
TCF12 hsa-mir-146a chr5: 160468252 chr5: 160468127-160468271(score=401) Regulation SRX100434 level 2 Blood H.sapiens
TCF12 hsa-mir-146a chr5: 160468251(NR_132748) chr5: 160467643-160468012(score=1000) Regulation SRX158110 level 1 Blood H.sapiens
TCF12 hsa-mir-146a chr5: 160468251(NR_132748) chr5: 160467705-160467948(score=940) Regulation SRX026635 level 1 Blood H.sapiens
TCF12 hsa-mir-146a chr5: 160468251(NR_132748) chr5: 160467715-160467929(score=699) Regulation SRX158109 level 1 Blood H.sapiens
TCF12 hsa-mir-146a chr5: 160468251(NR_132748) chr5: 160467722-160467976(score=620) Regulation SRX265225 level 1 Blood H.sapiens
TCF12 hsa-mir-146a chr5: 160468251(NR_132748) chr5: 160467740-160468010(score=1000) Regulation SRX236114 level 1 Blood H.sapiens
TCF12 hsa-mir-146a chr5: 160468251(NR_132748) chr5: 160463739-160464118(score=1000) Regulation SRX190356 level 1 Neural H.sapiens
TCF12 hsa-mir-146a chr5: 160468251(NR_132748) chr5: 160467581-160467838(score=544) Regulation SRX190356 level 1 Neural H.sapiens
TCF21 hsa-mir-146a chr5: 160468251(NR_132748) chr5: 160463766-160463940(score=477) Regulation SRX699733 level 1 Cardiovascular H.sapiens

TCF3 hsa-mir-146a chr5: 160468251(NR_132748) chr5: 160467582-160468231(score=1000) Regulation SRX236115 level 1 Blood H.sapiens
TCF3 hsa-mir-146a chr5: 160468251(NR_132748) chr5: 160467588-160468232(score=1000) Regulation SRX2043230 level 1 Blood H.sapiens
TCF3 hsa-mir-146a chr5: 160468251(NR_132748) chr5: 160467632-160467993(score=1000) Regulation SRX656347 level 1 Blood H.sapiens
TCF3 hsa-mir-146a chr5: 160468251(NR_132748) chr5: 160467635-160468078(score=1000) Regulation SRX2043226 level 1 Blood H.sapiens
TCF3 hsa-mir-146a chr5: 160468251(NR_132748) chr5: 160467659-160467939(score=1000) Regulation SRX158106 level 1 Blood H.sapiens
TCF4 hsa-mir-146a n/a n/a Activation 24561623 literature n/a H.sapiens
TCF7L2 hsa-mir-146a chr5: 160468251(NR_132748) chr5: 160467698-160467949(score=612) Regulation SRX097074 level 1 Blood H.sapiens
TCF7L2 hsa-mir-146a chr5: 160468251(NR_132748) chr5: 160467706-160467932(score=675) Regulation SRX097075 level 1 Blood H.sapiens
TCF7L2 hsa-mir-146a chr5: 160468251(NR_132748) chr5: 160467536-160467960(score=1000) Regulation SRX100942 level 1 Pancreas H.sapiens
TEAD4 hsa-mir-146a chr5: 160468251(NR_132748) chr5: 160463859-160464066(score=382) Regulation SRX190314 level 1 Neural H.sapiens
TFAP2C hsa-mir-146a chr5: 160468251(NR_132748) chr5: 160467567-160467993(score=1000) Regulation SRX190406 level 1 Epidermis H.sapiens
TGFB1 hsa-mir-146a n/a n/a Activation(feedback) 20375304 literature n/a H.sapiens
TLR2 hsa-mir-146a n/a n/a Activation(feedback) 20375304 literature n/a H.sapiens
TNFSF12 hsa-mir-146a n/a n/a Activation 20098732 literature n/a H.sapiens
TP53 hsa-mir-146a n/a n/a Repression 25757558 literature n/a H.sapiens
TRIM28 hsa-mir-146a chr5: 160468251(NR_132748) chr5: 160467761-160468103(score=851) Regulation SRX391683 level 1 Blood H.sapiens
USF1 hsa-mir-146a chr5: 160468251(NR_132748) chr5: 160467045-160467223(score=1000) Regulation SRX100572 level 1 Liver H.sapiens
USF2 hsa-mir-146a chr5: 160468251(NR_132748) chr5: 160467074-160467192(score=435) Regulation SRX150725 level 1 Liver H.sapiens
YAP1 hsa-mir-146a chr5: 160468251(NR_132748) chr5: 160463773-160464133(score=1000) Regulation SRX757326 level 1 Lung H.sapiens
YAP1 hsa-mir-146a chr5: 160468251(NR_132748) chr5: 160463776-160464132(score=1000) Regulation SRX757325 level 1 Lung H.sapiens
YAP1 hsa-mir-146a chr5: 160468251(NR_132748) chr5: 160463856-160464019(score=331) Regulation SRX1038945 level 1 Lung H.sapiens
YY1 hsa-mir-146a chr5: 160468252 chr5: 160468193-160468343(score=343) Regulation SRX100451 level 2 Blood H.sapiens
ZBTB16 hsa-mir-146a n/a n/a Repression 25705792 literature n/a H.sapiens
ZBTB33 hsa-mir-146a chr5: 160468251(NR_132748) chr5: 160463860-160463990(score=272) Regulation(feedback) SRX190255 level 1 Neural H.sapiens

ZMYND8	hsa-mir-146a	chr5: 160468251(NR_132748)	chr5: 160467864-160468195(score=485)	Regulation	SRX1819616	level 1	Prostate	H.sapiens
ZNF143	hsa-mir-146a	chr5: 160468252	chr5: 160468168-160468277(score=356)	Regulation	SRX150692	level 2	Blood	H.sapiens

Chr-chromosome; SRAID- ChIP-seq derived regulations; PMID-Reference in PubMed or literature-curated regulations; Level 1-predicted interactions; Level 2- promoter- supported by high-throughput experimental data; Tissue-The tissue from which the ChIP-seq data was derived [21].
