# Supplementary material for: Expression and Functional Analysis of Immuno-Micro-RNAs mir-146a and mir-326 in Colorectal Cancer
Source: Curr Issues Mol Biol. 2024 Jul 5;46(7):7065–85. doi: 10.3390/cimb46070421 (PMC11276483; doi:10.3390/cimb46070421)
Supplement: Supplementary file 1 [file cimb-46-00421-s001.zip › Table S2-Transcription factors that regulate the expression of mir-326.pdf]

Table S2-Transcription factors that regulate the expression of mir-326

TF name	miRNA name	TSS	Binding site	Action type	SRAID/PMID	Evidence	Tissue	Species
AHR	hsa-mir-326	chr11: 75335186	chr11: 75337251-75337452(score=1000)	Regulation	SRX2378695	level 1	Breast	H.sapiens
AR	hsa-mir-326	chr11: 75351660	chr11: 75351832-75351943(score=353)	Regulation	SRX250092	level 2	Breast	H.sapiens
AR	hsa-mir-326	chr11: 75351831(NM_004041)	chr11: 75351212-75351373(score=268)	Regulation	SRX250092	level 1	Breast	H.sapiens
AR	hsa-mir-326	chr11: 75351831(NM_020251)	chr11: 75351212-75351373(score=268)	Regulation	SRX250092	level 1	Breast	H.sapiens
AR	hsa-mir-326	chr11: 75351831(NM_004041)	chr11: 75355177-75355348(score=538)	Regulation	SRX433201	level 1	Prostate	H.sapiens
AR	hsa-mir-326	chr11: 75351831(NM_020251)	chr11: 75355177-75355348(score=538)	Regulation	SRX433201	level 1	Prostate	H.sapiens
ARNTL	hsa-mir-326	chr11: 75351831(NM_004041)	chr11: 75351034-75351370(score=640)	Regulation	SRX666557	level 1	Breast	H.sapiens
ARNTL	hsa-mir-326	chr11: 75351831(NM_004041)	chr11: 75351769-75352160(score=1000)	Regulation	SRX666557	level 1	Breast	H.sapiens
ARNTL	hsa-mir-326	chr11: 75351831(NM_020251)	chr11: 75351034-75351370(score=640)	Regulation	SRX666557	level 1	Breast	H.sapiens
ARNTL	hsa-mir-326	chr11: 75351831(NM_020251)	chr11: 75351769-75352160(score=1000)	Regulation	SRX666557	level 1	Breast	H.sapiens
BRD2	hsa-mir-326	chr11: 75351831(NM_004041)	chr11: 75351048-75351288(score=366)	Regulation	SRX471867	level 1	Prostate	H.sapiens
BRD2	hsa-mir-326	chr11: 75351831(NM_004041)	chr11: 75351065-75351228(score=314)	Regulation	SRX471868	level 1	Prostate	H.sapiens
BRD2	hsa-mir-326	chr11: 75351831(NM_020251)	chr11: 75351048-75351288(score=366)	Regulation	SRX471867	level 1	Prostate	H.sapiens
BRD2	hsa-mir-326	chr11: 75351831(NM_020251)	chr11: 75351065-75351228(score=314)	Regulation	SRX471868	level 1	Prostate	H.sapiens
BRD3	hsa-mir-326	chr11: 75351831(NM_004041)	chr11: 75350995-75351222(score=322)	Regulation	SRX471871	level 1	Prostate	H.sapiens

BRD3 hsa-mir-326 chr11: 75351831(NM_004041) chr11: 75351038-75351314(score=342) Regulation SRX471870 level 1 Prostate H.sapiens
BRD3 hsa-mir-326 chr11: 75351831(NM_020251) chr11: 75350995-75351222(score=322) Regulation SRX471871 level 1 Prostate H.sapiens
BRD3 hsa-mir-326 chr11: 75351831(NM_020251) chr11: 75351038-75351314(score=342) Regulation SRX471870 level 1 Prostate H.sapiens
BRD4 hsa-mir-326 chr11: 75351831(NM_004041) chr11: 75351873-75352044(score=291) Regulation SRX1134739 level 1 Blood H.sapiens
BRD4 hsa-mir-326 chr11: 75351831(NM_004041) chr11: 75351878-75352106(score=397) Regulation SRX448946 level 1 Blood H.sapiens
BRD4 hsa-mir-326 chr11: 75351831(NM_004041) chr11: 75352300-75352466(score=429) Regulation SRX1134763 level 1 Blood H.sapiens
BRD4 hsa-mir-326 chr11: 75351831(NM_020251) chr11: 75351873-75352044(score=291) Regulation SRX1134739 level 1 Blood H.sapiens
BRD4 hsa-mir-326 chr11: 75351831(NM_020251) chr11: 75351878-75352106(score=397) Regulation SRX448946 level 1 Blood H.sapiens
BRD4 hsa-mir-326 chr11: 75351831(NM_020251) chr11: 75352300-75352466(score=429) Regulation SRX1134763 level 1 Blood H.sapiens
BRD4 hsa-mir-326 chr11: 75351831(NM_004041) chr11: 75351749-75352007(score=441) Regulation SRX2267450 level 1 Digestive tract H.sapiens
BRD4 hsa-mir-326 chr11: 75351831(NM_020251) chr11: 75351749-75352007(score=441) Regulation SRX2267450 level 1 Digestive tract H.sapiens
BRD4 hsa-mir-326 chr11: 75335186 chr11: 75334588-75334911(score=402) Regulation SRX2267458 level 1 Lung H.sapiens
BRD4 hsa-mir-326 chr11: 75335186 chr11: 75334604-75334837(score=383) Regulation SRX2267454 level 1 Lung H.sapiens
BRD4 hsa-mir-326 chr11: 75351831(NM_004041) chr11: 75351708-75352037(score=523) Regulation SRX2267458 level 1 Lung H.sapiens
BRD4 hsa-mir-326 chr11: 75351831(NM_004041) chr11: 75351743-75352350(score=568) Regulation SRX2267454 level 1 Lung H.sapiens
BRD4 hsa-mir-326 chr11: 75351831(NM_020251) chr11: 75351708-75352037(score=523) Regulation SRX2267458 level 1 Lung H.sapiens
BRD4 hsa-mir-326 chr11: 75351831(NM_020251) chr11: 75351743-75352350(score=568) Regulation SRX2267454 level 1 Lung H.sapiens
BRD4 hsa-mir-326 chr11: 75351831(NM_004041) chr11: 75351050-75351246(score=260) Regulation SRX471874 level 1 Prostate H.sapiens

BRD4 hsa-mir-326 chr11: 75351831(NM_020251) chr11: 75351050-75351246(score=260) Regulation SRX471874 level 1 Prostate H.sapiens
CBFB hsa-mir-326 chr11: 75351831(NM_004041) chr11: 75351291-75351589(score=373) Regulation SRX265218 level 1 Blood H.sapiens
CBFB hsa-mir-326 chr11: 75351831(NM_020251) chr11: 75351291-75351589(score=373) Regulation SRX265218 level 1 Blood H.sapiens
CREB1 hsa-mir-326 chr11: 75351660 chr11: 75351659-75351911(score=483) Regulation SRX752907 level 2 Prostate H.sapiens
CREB1 hsa-mir-326 chr11: 75351831(NM_004041) chr11: 75351694-75351972(score=524) Regulation SRX1212238 level 1 Prostate H.sapiens
CREB1 hsa-mir-326 chr11: 75351831(NM_020251) chr11: 75351694-75351972(score=524) Regulation SRX1212238 level 1 Prostate H.sapiens
CTCF hsa-mir-326 chr11: 75335186 chr11: 75339746-75340014(score=363) Regulation SRX356482 level 1 Blood H.sapiens
CTCF hsa-mir-326 chr11: 75335186 chr11: 75339774-75340088(score=390) Regulation SRX356647 level 1 Blood H.sapiens
CTCF hsa-mir-326 chr11: 75335186 chr11: 75339775-75339899(score=757) Regulation SRX150690 level 1 Blood H.sapiens
CTCF hsa-mir-326 chr11: 75335186 chr11: 75339795-75339892(score=646) Regulation SRX381545 level 1 Blood H.sapiens
CTCF hsa-mir-326 chr11: 75335186 chr11: 75339802-75340047(score=329) Regulation SRX356786 level 1 Blood H.sapiens
CTCF hsa-mir-326 chr11: 75351831(NM_004041) chr11: 75352284-75352521(score=663) Regulation SRX186704 level 1 Blood H.sapiens
CTCF hsa-mir-326 chr11: 75351831(NM_004041) chr11: 75352288-75352506(score=493) Regulation SRX2655462 level 1 Blood H.sapiens
CTCF hsa-mir-326 chr11: 75351831(NM_004041) chr11: 75352307-75352521(score=941) Regulation SRX1738531 level 1 Blood H.sapiens
CTCF hsa-mir-326 chr11: 75351831(NM_004041) chr11: 75352319-75352488(score=906) Regulation SRX150487 level 1 Blood H.sapiens
CTCF hsa-mir-326 chr11: 75351831(NM_004041) chr11: 75352319-75352491(score=688) Regulation SRX1091813 level 1 Blood H.sapiens
CTCF hsa-mir-326 chr11: 75351831(NM_004041) chr11: 75352331-75352492(score=761) Regulation SRX190276 level 1 Blood H.sapiens
CTCF hsa-mir-326 chr11: 75351831(NM_004041) chr11: 75352349-75352482(score=596) Regulation SRX103010 level 1 Blood H.sapiens

CTCF hsa-mir-326 chr11: 75351831(NM_004041) chr11: 75352350-75352478(score=590) Regulation SRX031247 level 1 Blood H.sapiens
CTCF hsa-mir-326 chr11: 75351831(NM_004041) chr11: 75352365-75352462(score=675) Regulation SRX381545 level 1 Blood H.sapiens
CTCF hsa-mir-326 chr11: 75351831(NM_020251) chr11: 75352284-75352521(score=663) Regulation SRX186704 level 1 Blood H.sapiens
CTCF hsa-mir-326 chr11: 75351831(NM_020251) chr11: 75352288-75352506(score=493) Regulation SRX2655462 level 1 Blood H.sapiens
CTCF hsa-mir-326 chr11: 75351831(NM_020251) chr11: 75352307-75352521(score=941) Regulation SRX1738531 level 1 Blood H.sapiens
CTCF hsa-mir-326 chr11: 75351831(NM_020251) chr11: 75352319-75352488(score=906) Regulation SRX150487 level 1 Blood H.sapiens
CTCF hsa-mir-326 chr11: 75351831(NM_020251) chr11: 75352319-75352491(score=688) Regulation SRX1091813 level 1 Blood H.sapiens
CTCF hsa-mir-326 chr11: 75351831(NM_020251) chr11: 75352331-75352492(score=761) Regulation SRX190276 level 1 Blood H.sapiens
CTCF hsa-mir-326 chr11: 75351831(NM_020251) chr11: 75352349-75352482(score=596) Regulation SRX103010 level 1 Blood H.sapiens
CTCF hsa-mir-326 chr11: 75351831(NM_020251) chr11: 75352350-75352478(score=590) Regulation SRX031247 level 1 Blood H.sapiens
CTCF hsa-mir-326 chr11: 75351831(NM_020251) chr11: 75352365-75352462(score=675) Regulation SRX381545 level 1 Blood H.sapiens
CTCF hsa-mir-326 chr11: 75351660 chr11: 75351600-75351702(score=461) Regulation SRX103004 level 2 Breast H.sapiens
CTCF hsa-mir-326 chr11: 75351660 chr11: 75351600-75351707(score=608) Regulation SRX188952 level 2 Breast H.sapiens
CTCF hsa-mir-326 chr11: 75335186 chr11: 75339756-75339926(score=1000) Regulation SRX103007 level 1 Breast H.sapiens
CTCF hsa-mir-326 chr11: 75335186 chr11: 75339759-75339904(score=918) Regulation SRX188955 level 1 Breast H.sapiens
CTCF hsa-mir-326 chr11: 75335186 chr11: 75339774-75339889(score=686) Regulation SRX188952 level 1 Breast H.sapiens
CTCF hsa-mir-326 chr11: 75335186 chr11: 75339781-75339885(score=557) Regulation SRX103004 level 1 Breast H.sapiens
CTCF hsa-mir-326 chr11: 75351831(NM_004041) chr11: 75351144-75351307(score=368) Regulation SRX188955 level 1 Breast H.sapiens

CTCF hsa-mir-326 chr11: 75351831(NM_004041) chr11: 75351180-75351304(score=288) Regulation SRX188952 level 1 Breast H.sapiens
CTCF hsa-mir-326 chr11: 75351831(NM_020251) chr11: 75351144-75351307(score=368) Regulation SRX188955 level 1 Breast H.sapiens
CTCF hsa-mir-326 chr11: 75351831(NM_020251) chr11: 75351180-75351304(score=288) Regulation SRX188952 level 1 Breast H.sapiens
CTCF hsa-mir-326 chr11: 75351831(NM_004041) chr11: 75352335-75352467(score=486) Regulation SRX080411 level 1 Digestive tract H.sapiens
CTCF hsa-mir-326 chr11: 75351831(NM_004041) chr11: 75352356-75352476(score=628) Regulation SRX080352 level 1 Digestive tract H.sapiens
CTCF hsa-mir-326 chr11: 75351831(NM_020251) chr11: 75352335-75352467(score=486) Regulation SRX080411 level 1 Digestive tract H.sapiens
CTCF hsa-mir-326 chr11: 75351831(NM_020251) chr11: 75352356-75352476(score=628) Regulation SRX080352 level 1 Digestive tract H.sapiens
CTCF hsa-mir-326 chr11: 75351660 chr11: 75351575-75351707(score=358) Regulation SRX1091821 level 2 Gonad H.sapiens
CTCF hsa-mir-326 chr11: 75335186 chr11: 75339755-75339953(score=837) Regulation SRX1091821 level 1 Gonad H.sapiens
CTCF hsa-mir-326 chr11: 75351831(NM_004041) chr11: 75351489-75351751(score=489) Regulation SRX1594218 level 1 Kidney H.sapiens
CTCF hsa-mir-326 chr11: 75351831(NM_020251) chr11: 75351489-75351751(score=489) Regulation SRX1594218 level 1 Kidney H.sapiens
CTCF hsa-mir-326 chr11: 75351831(NM_004041) chr11: 75352345-75352472(score=559) Regulation SRX100531 level 1 Liver H.sapiens
CTCF hsa-mir-326 chr11: 75351831(NM_004041) chr11: 75352356-75352448(score=477) Regulation SRX080346 level 1 Liver H.sapiens
CTCF hsa-mir-326 chr11: 75351831(NM_020251) chr11: 75352345-75352472(score=559) Regulation SRX100531 level 1 Liver H.sapiens
CTCF hsa-mir-326 chr11: 75351831(NM_020251) chr11: 75352356-75352448(score=477) Regulation SRX080346 level 1 Liver H.sapiens
CTCF hsa-mir-326 chr11: 75335186 chr11: 75339696-75339979(score=566) Regulation SRX186777 level 1 Lung H.sapiens
CTCF hsa-mir-326 chr11: 75335186 chr11: 75339767-75339922(score=764) Regulation SRX102988 level 1 Lung H.sapiens
CTCF hsa-mir-326 chr11: 75335186 chr11: 75339784-75339898(score=869) Regulation SRX102984 level 1 Uterus H.sapiens

CTCF hsa-mir-326 chr11: 75335186 chr11: 75339784-75339898(score=878) Regulation SRX031245 level 1 Uterus H.sapiens
CTCFL hsa-mir-326 chr11: 75351831(NM_004041) chr11: 75351315-75351725(score=1000) Regulation SRX1091814 level 1 Blood H.sapiens
CTCFL hsa-mir-326 chr11: 75351831(NM_004041) chr11: 75351798-75351989(score=718) Regulation SRX1091814 level 1 Blood H.sapiens
CTCFL hsa-mir-326 chr11: 75351831(NM_020251) chr11: 75351315-75351725(score=1000) Regulation SRX1091814 level 1 Blood H.sapiens
CTCFL hsa-mir-326 chr11: 75351831(NM_020251) chr11: 75351798-75351989(score=718) Regulation SRX1091814 level 1 Blood H.sapiens
E2F1 hsa-mir-326 chr11: 75351660 chr11: 75351647-75351886(score=389) Regulation(feedback) SRX150446 level 2 Uterus H.sapiens
EBF1 hsa-mir-326 chr11: 75351831(NM_004041) chr11: 75351175-75351286(score=310) Regulation SRX100431 level 1 Blood H.sapiens
EBF1 hsa-mir-326 chr11: 75351831(NM_020251) chr11: 75351175-75351286(score=310) Regulation SRX100431 level 1 Blood H.sapiens
EGR1 hsa-mir-326 chr11: 75351660 chr11: 75351648-75351845(score=490) Regulation SRX100459 level 2 Blood H.sapiens
EGR1 hsa-mir-326 chr11: 75351831(NM_004041) chr11: 75352266-75352426(score=847) Regulation SRX100459 level 1 Blood H.sapiens
EGR1 hsa-mir-326 chr11: 75351831(NM_020251) chr11: 75352266-75352426(score=847) Regulation SRX100459 level 1 Blood H.sapiens
ELF1 hsa-mir-326 chr11: 75351831(NM_004041) chr11: 75352272-75352509(score=1000) Regulation SRX100539 level 1 Blood H.sapiens
ELF1 hsa-mir-326 chr11: 75351831(NM_020251) chr11: 75352272-75352509(score=1000) Regulation SRX100539 level 1 Blood H.sapiens
ELF1 hsa-mir-326 chr11: 75351831(NM_004041) chr11: 75352288-75352481(score=472) Regulation SRX100460 level 1 Liver H.sapiens
ELF1 hsa-mir-326 chr11: 75351831(NM_020251) chr11: 75352288-75352481(score=472) Regulation SRX100460 level 1 Liver H.sapiens
ELF3 hsa-mir-326 chr11: 75335186 chr11: 75337256-75337641(score=803) Regulation SRX825394 level 1 Adipocyte H.sapiens
ELF3 hsa-mir-326 chr11: 75335186 chr11: 75337258-75337641(score=555) Regulation SRX1389380 level 1 Adipocyte H.sapiens
EOMES hsa-mir-326 chr11: 75351831(NM_004041) chr11: 75352278-75352416(score=346) Regulation SRX035159 level 1 Pluripotent stem cell H.sapiens

EOMES hsa-mir-326 chr11: 75351831(NM_020251) chr11: 75352278-75352416(score=346) Regulation SRX035159 level 1 Pluripotent stem cell H.sapiens
EP300 hsa-mir-326 chr11: 75351831(NM_004041) chr11: 75352219-75352581(score=887) Regulation SRX265233 level 1 Blood H.sapiens
EP300 hsa-mir-326 chr11: 75351831(NM_020251) chr11: 75352219-75352581(score=887) Regulation SRX265233 level 1 Blood H.sapiens
EP300 hsa-mir-326 chr11: 75351831(NM_004041) chr11: 75351202-75351345(score=668) Regulation SRX176883 level 1 Breast H.sapiens
EP300 hsa-mir-326 chr11: 75351831(NM_020251) chr11: 75351202-75351345(score=668) Regulation SRX176883 level 1 Breast H.sapiens
EP300 hsa-mir-326 chr11: 75351831(NM_004041) chr11: 75352285-75352422(score=512) Regulation SRX100544 level 1 Liver H.sapiens
EP300 hsa-mir-326 chr11: 75351831(NM_020251) chr11: 75352285-75352422(score=512) Regulation SRX100544 level 1 Liver H.sapiens
EP300 hsa-mir-326 chr11: 75351831(NM_004041) chr11: 75355160-75355318(score=323) Regulation SRX190215 level 1 Uterus H.sapiens
EP300 hsa-mir-326 chr11: 75351831(NM_020251) chr11: 75355160-75355318(score=323) Regulation SRX190215 level 1 Uterus H.sapiens
ERG hsa-mir-326 chr11: 75351660 chr11: 75351602-75351914(score=1000) Regulation SRX063920 level 2 Blood H.sapiens
ERG hsa-mir-326 chr11: 75351660 chr11: 75351620-75351887(score=297) Regulation SRX063919 level 2 Blood H.sapiens
ERG hsa-mir-326 chr11: 75351831(NM_004041) chr11: 75351321-75351680(score=724) Regulation SRX265230 level 1 Blood H.sapiens
ERG hsa-mir-326 chr11: 75351831(NM_004041) chr11: 75351363-75351618(score=352) Regulation SRX026653 level 1 Blood H.sapiens
ERG hsa-mir-326 chr11: 75351831(NM_004041) chr11: 75351467-75351991(score=1000) Regulation SRX063922 level 1 Blood H.sapiens
ERG hsa-mir-326 chr11: 75351831(NM_004041) chr11: 75351486-75351947(score=1000) Regulation SRX063921 level 1 Blood H.sapiens
ERG hsa-mir-326 chr11: 75351831(NM_004041) chr11: 75351805-75352571(score=1000) Regulation SRX265230 level 1 Blood H.sapiens
ERG hsa-mir-326 chr11: 75351831(NM_004041) chr11: 75352078-75352540(score=849) Regulation SRX682375 level 1 Blood H.sapiens
ERG hsa-mir-326 chr11: 75351831(NM_004041) chr11: 75352217-75352624(score=1000) Regulation SRX026639 level 1 Blood H.sapiens

ERG hsa-mir-326 chr11: 75351831(NM_004041) chr11: 75352277-75352536(score=493) Regulation SRX1514277 level 1 Blood H.sapiens
ERG hsa-mir-326 chr11: 75351831(NM_004041) chr11: 75352280-75352518(score=809) Regulation SRX682376 level 1 Blood H.sapiens
ERG hsa-mir-326 chr11: 75351831(NM_020251) chr11: 75351321-75351680(score=724) Regulation SRX265230 level 1 Blood H.sapiens
ERG hsa-mir-326 chr11: 75351831(NM_020251) chr11: 75351363-75351618(score=352) Regulation SRX026653 level 1 Blood H.sapiens
ERG hsa-mir-326 chr11: 75351831(NM_020251) chr11: 75351467-75351991(score=1000) Regulation SRX063922 level 1 Blood H.sapiens
ERG hsa-mir-326 chr11: 75351831(NM_020251) chr11: 75351486-75351947(score=1000) Regulation SRX063921 level 1 Blood H.sapiens
ERG hsa-mir-326 chr11: 75351831(NM_020251) chr11: 75351805-75352571(score=1000) Regulation SRX265230 level 1 Blood H.sapiens
ERG hsa-mir-326 chr11: 75351831(NM_020251) chr11: 75352078-75352540(score=849) Regulation SRX682375 level 1 Blood H.sapiens
ERG hsa-mir-326 chr11: 75351831(NM_020251) chr11: 75352217-75352624(score=1000) Regulation SRX026639 level 1 Blood H.sapiens
ERG hsa-mir-326 chr11: 75351831(NM_020251) chr11: 75352277-75352536(score=493) Regulation SRX1514277 level 1 Blood H.sapiens
ERG hsa-mir-326 chr11: 75351831(NM_020251) chr11: 75352280-75352518(score=809) Regulation SRX682376 level 1 Blood H.sapiens
ERG hsa-mir-326 chr11: 75351831(NM_004041) chr11: 75351480-75351681(score=474) Regulation SRX063918 level 1 Breast H.sapiens
ERG hsa-mir-326 chr11: 75351831(NM_020251) chr11: 75351480-75351681(score=474) Regulation SRX063918 level 1 Breast H.sapiens
ESR1 hsa-mir-326 chr11: 75351831(NM_004041) chr11: 75351143-75351646(score=1000) Regulation SRX036635 level 1 Bone H.sapiens
ESR1 hsa-mir-326 chr11: 75351831(NM_020251) chr11: 75351143-75351646(score=1000) Regulation SRX036635 level 1 Bone H.sapiens
ESR1 hsa-mir-326 chr11: 75351831(NM_004041) chr11: 75351062-75351547(score=1000) Regulation SRX1995230 level 1 Breast H.sapiens
ESR1 hsa-mir-326 chr11: 75351831(NM_004041) chr11: 75351068-75351567(score=1000) Regulation SRX1995229 level 1 Breast H.sapiens
ESR1 hsa-mir-326 chr11: 75351831(NM_004041) chr11: 75351121-75351404(score=621) Regulation SRX1012625 level 1 Breast H.sapiens

ESR1 hsa-mir-326 chr11: 75351831(NM_004041) chr11: 75351131-75351409(score=1000) Regulation SRX2513520 level 1 Breast H.sapiens
ESR1 hsa-mir-326 chr11: 75351831(NM_004041) chr11: 75351132-75351479(score=1000) Regulation SRX261273 level 1 Breast H.sapiens
ESR1 hsa-mir-326 chr11: 75351831(NM_004041) chr11: 75351132-75351479(score=1000) Regulation SRX673710 level 1 Breast H.sapiens
ESR1 hsa-mir-326 chr11: 75351831(NM_004041) chr11: 75351133-75351482(score=1000) Regulation SRX323399 level 1 Breast H.sapiens
ESR1 hsa-mir-326 chr11: 75351831(NM_004041) chr11: 75351135-75351420(score=817) Regulation SRX3110725 level 1 Breast H.sapiens
ESR1 hsa-mir-326 chr11: 75351831(NM_004041) chr11: 75351139-75351405(score=584) Regulation SRX1012631 level 1 Breast H.sapiens
ESR1 hsa-mir-326 chr11: 75351831(NM_004041) chr11: 75351142-75351390(score=1000) Regulation SRX2513521 level 1 Breast H.sapiens
ESR1 hsa-mir-326 chr11: 75351831(NM_004041) chr11: 75351143-75351404(score=1000) Regulation SRX2513519 level 1 Breast H.sapiens
ESR1 hsa-mir-326 chr11: 75351831(NM_004041) chr11: 75351144-75351391(score=1000) Regulation SRX2513518 level 1 Breast H.sapiens
ESR1 hsa-mir-326 chr11: 75351831(NM_004041) chr11: 75351145-75351413(score=1000) Regulation SRX330067 level 1 Breast H.sapiens
ESR1 hsa-mir-326 chr11: 75351831(NM_004041) chr11: 75351149-75351416(score=1000) Regulation SRX2513517 level 1 Breast H.sapiens
ESR1 hsa-mir-326 chr11: 75351831(NM_004041) chr11: 75351150-75351478(score=1000) Regulation SRX747801 level 1 Breast H.sapiens
ESR1 hsa-mir-326 chr11: 75351831(NM_004041) chr11: 75351151-75351409(score=566) Regulation SRX3110722 level 1 Breast H.sapiens
ESR1 hsa-mir-326 chr11: 75351831(NM_004041) chr11: 75351154-75351411(score=1000) Regulation SRX176861 level 1 Breast H.sapiens
ESR1 hsa-mir-326 chr11: 75351831(NM_004041) chr11: 75351155-75351431(score=1000) Regulation SRX968963 level 1 Breast H.sapiens
ESR1 hsa-mir-326 chr11: 75351831(NM_004041) chr11: 75351156-75351444(score=1000) Regulation SRX1116525 level 1 Breast H.sapiens
ESR1 hsa-mir-326 chr11: 75351831(NM_004041) chr11: 75351158-75351351(score=659) Regulation SRX969013 level 1 Breast H.sapiens
ESR1 hsa-mir-326 chr11: 75351831(NM_004041) chr11: 75351159-75351365(score=785) Regulation SRX969014 level 1 Breast H.sapiens

ESR1 hsa-mir-326 chr11: 75351831(NM_004041) chr11: 75351159-75351383(score=497) Regulation SRX1161200 level 1 Breast H.sapiens
ESR1 hsa-mir-326 chr11: 75351831(NM_004041) chr11: 75351160-75351466(score=1000) Regulation SRX1116524 level 1 Breast H.sapiens
ESR1 hsa-mir-326 chr11: 75351831(NM_004041) chr11: 75351161-75351407(score=1000) Regulation SRX176860 level 1 Breast H.sapiens
ESR1 hsa-mir-326 chr11: 75351831(NM_004041) chr11: 75351162-75351365(score=973) Regulation SRX792932 level 1 Breast H.sapiens
ESR1 hsa-mir-326 chr11: 75351831(NM_004041) chr11: 75351163-75351394(score=512) Regulation SRX1012628 level 1 Breast H.sapiens
ESR1 hsa-mir-326 chr11: 75351831(NM_004041) chr11: 75351164-75351381(score=1000) Regulation SRX968964 level 1 Breast H.sapiens
ESR1 hsa-mir-326 chr11: 75351831(NM_004041) chr11: 75351167-75351430(score=1000) Regulation SRX1531791 level 1 Breast H.sapiens
ESR1 hsa-mir-326 chr11: 75351831(NM_004041) chr11: 75351168-75351368(score=1000) Regulation SRX792933 level 1 Breast H.sapiens
ESR1 hsa-mir-326 chr11: 75351831(NM_004041) chr11: 75351168-75351385(score=618) Regulation SRX1161201 level 1 Breast H.sapiens
ESR1 hsa-mir-326 chr11: 75351831(NM_004041) chr11: 75351169-75351395(score=919) Regulation SRX3110721 level 1 Breast H.sapiens
ESR1 hsa-mir-326 chr11: 75351831(NM_004041) chr11: 75351172-75351360(score=639) Regulation SRX371473 level 1 Breast H.sapiens
ESR1 hsa-mir-326 chr11: 75351831(NM_004041) chr11: 75351172-75351362(score=1000) Regulation SRX176856 level 1 Breast H.sapiens
ESR1 hsa-mir-326 chr11: 75351831(NM_004041) chr11: 75351172-75351393(score=1000) Regulation SRX176857 level 1 Breast H.sapiens
ESR1 hsa-mir-326 chr11: 75351831(NM_004041) chr11: 75351173-75351362(score=576) Regulation SRX371478 level 1 Breast H.sapiens
ESR1 hsa-mir-326 chr11: 75351831(NM_004041) chr11: 75351174-75351367(score=803) Regulation SRX2513523 level 1 Breast H.sapiens
ESR1 hsa-mir-326 chr11: 75351831(NM_004041) chr11: 75351179-75351383(score=1000) Regulation SRX176858 level 1 Breast H.sapiens
ESR1 hsa-mir-326 chr11: 75351831(NM_004041) chr11: 75351180-75351399(score=1000) Regulation SRX194575 level 1 Breast H.sapiens
ESR1 hsa-mir-326 chr11: 75351831(NM_004041) chr11: 75351181-75351382(score=1000) Regulation SRX176859 level 1 Breast H.sapiens

ESR1 hsa-mir-326 chr11: 75351831(NM_004041) chr11: 75351181-75351449(score=767) Regulation SRX747782 level 1 Breast H.sapiens
ESR1 hsa-mir-326 chr11: 75351831(NM_004041) chr11: 75351182-75351399(score=735) Regulation SRX1469949 level 1 Breast H.sapiens
ESR1 hsa-mir-326 chr11: 75351831(NM_004041) chr11: 75351183-75351437(score=1000) Regulation SRX730450 level 1 Breast H.sapiens
ESR1 hsa-mir-326 chr11: 75351831(NM_004041) chr11: 75351183-75351454(score=1000) Regulation SRX323400 level 1 Breast H.sapiens
ESR1 hsa-mir-326 chr11: 75351831(NM_004041) chr11: 75351184-75351375(score=438) Regulation SRX1961161 level 1 Breast H.sapiens
ESR1 hsa-mir-326 chr11: 75351831(NM_004041) chr11: 75351186-75351391(score=907) Regulation SRX1336216 level 1 Breast H.sapiens
ESR1 hsa-mir-326 chr11: 75351831(NM_004041) chr11: 75351187-75351363(score=488) Regulation SRX330070 level 1 Breast H.sapiens
ESR1 hsa-mir-326 chr11: 75351831(NM_004041) chr11: 75351189-75351410(score=804) Regulation SRX747778 level 1 Breast H.sapiens
ESR1 hsa-mir-326 chr11: 75351831(NM_004041) chr11: 75351190-75351395(score=656) Regulation SRX747777 level 1 Breast H.sapiens
ESR1 hsa-mir-326 chr11: 75351831(NM_004041) chr11: 75351191-75351349(score=467) Regulation SRX330069 level 1 Breast H.sapiens
ESR1 hsa-mir-326 chr11: 75351831(NM_004041) chr11: 75351193-75351336(score=507) Regulation SRX371469 level 1 Breast H.sapiens
ESR1 hsa-mir-326 chr11: 75351831(NM_004041) chr11: 75351193-75351386(score=878) Regulation SRX026530 level 1 Breast H.sapiens
ESR1 hsa-mir-326 chr11: 75351831(NM_004041) chr11: 75351195-75351385(score=558) Regulation SRX469396 level 1 Breast H.sapiens
ESR1 hsa-mir-326 chr11: 75351831(NM_004041) chr11: 75351195-75351386(score=564) Regulation SRX1469951 level 1 Breast H.sapiens
ESR1 hsa-mir-326 chr11: 75351831(NM_004041) chr11: 75351197-75351368(score=481) Regulation SRX1531796 level 1 Breast H.sapiens
ESR1 hsa-mir-326 chr11: 75351831(NM_004041) chr11: 75351198-75351373(score=475) Regulation SRX730451 level 1 Breast H.sapiens
ESR1 hsa-mir-326 chr11: 75351831(NM_004041) chr11: 75351198-75351381(score=519) Regulation SRX1469950 level 1 Breast H.sapiens
ESR1 hsa-mir-326 chr11: 75351831(NM_004041) chr11: 75351199-75351383(score=483) Regulation SRX1961156 level 1 Breast H.sapiens

ESR1 hsa-mir-326 chr11: 75351831(NM_004041) chr11: 75351199-75351400(score=987) Regulation SRX747805 level 1 Breast H.sapiens
ESR1 hsa-mir-326 chr11: 75351831(NM_004041) chr11: 75351200-75351371(score=833) Regulation SRX371467 level 1 Breast H.sapiens
ESR1 hsa-mir-326 chr11: 75351831(NM_004041) chr11: 75351200-75351416(score=453) Regulation SRX3110723 level 1 Breast H.sapiens
ESR1 hsa-mir-326 chr11: 75351831(NM_004041) chr11: 75351201-75351406(score=425) Regulation SRX747781 level 1 Breast H.sapiens
ESR1 hsa-mir-326 chr11: 75351831(NM_004041) chr11: 75351202-75351339(score=454) Regulation SRX107369 level 1 Breast H.sapiens
ESR1 hsa-mir-326 chr11: 75351831(NM_004041) chr11: 75351202-75351370(score=817) Regulation SRX194569 level 1 Breast H.sapiens
ESR1 hsa-mir-326 chr11: 75351831(NM_004041) chr11: 75351203-75351335(score=449) Regulation SRX100419 level 1 Breast H.sapiens
ESR1 hsa-mir-326 chr11: 75351831(NM_004041) chr11: 75351203-75351342(score=960) Regulation SRX003939 level 1 Breast H.sapiens
ESR1 hsa-mir-326 chr11: 75351831(NM_004041) chr11: 75351210-75351356(score=657) Regulation SRX1585244 level 1 Breast H.sapiens
ESR1 hsa-mir-326 chr11: 75351831(NM_004041) chr11: 75351211-75351377(score=399) Regulation SRX968967 level 1 Breast H.sapiens
ESR1 hsa-mir-326 chr11: 75351831(NM_004041) chr11: 75351211-75351378(score=406) Regulation SRX1961155 level 1 Breast H.sapiens
ESR1 hsa-mir-326 chr11: 75351831(NM_004041) chr11: 75351213-75351350(score=604) Regulation SRX003938 level 1 Breast H.sapiens
ESR1 hsa-mir-326 chr11: 75351831(NM_004041) chr11: 75351213-75351353(score=465) Regulation SRX371479 level 1 Breast H.sapiens
ESR1 hsa-mir-326 chr11: 75351831(NM_004041) chr11: 75351214-75351353(score=537) Regulation SRX100584 level 1 Breast H.sapiens
ESR1 hsa-mir-326 chr11: 75351831(NM_004041) chr11: 75351214-75351365(score=535) Regulation SRX261274 level 1 Breast H.sapiens
ESR1 hsa-mir-326 chr11: 75351831(NM_004041) chr11: 75351214-75351365(score=538) Regulation SRX730426 level 1 Breast H.sapiens
ESR1 hsa-mir-326 chr11: 75351831(NM_004041) chr11: 75351214-75351512(score=1000) Regulation SRX323398 level 1 Breast H.sapiens
ESR1 hsa-mir-326 chr11: 75351831(NM_004041) chr11: 75351218-75351352(score=460) Regulation SRX747802 level 1 Breast H.sapiens

ESR1 hsa-mir-326 chr11: 75351831(NM_004041) chr11: 75351229-75351351(score=412) Regulation SRX673753 level 1 Breast H.sapiens
ESR1 hsa-mir-326 chr11: 75351831(NM_004041) chr11: 75351241-75351365(score=378) Regulation SRX3110738 level 1 Breast H.sapiens
ESR1 hsa-mir-326 chr11: 75351831(NM_020251) chr11: 75351062-75351547(score=1000) Regulation SRX1995230 level 1 Breast H.sapiens
ESR1 hsa-mir-326 chr11: 75351831(NM_020251) chr11: 75351068-75351567(score=1000) Regulation SRX1995229 level 1 Breast H.sapiens
ESR1 hsa-mir-326 chr11: 75351831(NM_020251) chr11: 75351121-75351404(score=621) Regulation SRX1012625 level 1 Breast H.sapiens
ESR1 hsa-mir-326 chr11: 75351831(NM_020251) chr11: 75351131-75351409(score=1000) Regulation SRX2513520 level 1 Breast H.sapiens
ESR1 hsa-mir-326 chr11: 75351831(NM_020251) chr11: 75351132-75351479(score=1000) Regulation SRX261273 level 1 Breast H.sapiens
ESR1 hsa-mir-326 chr11: 75351831(NM_020251) chr11: 75351132-75351479(score=1000) Regulation SRX673710 level 1 Breast H.sapiens
ESR1 hsa-mir-326 chr11: 75351831(NM_020251) chr11: 75351133-75351482(score=1000) Regulation SRX323399 level 1 Breast H.sapiens
ESR1 hsa-mir-326 chr11: 75351831(NM_020251) chr11: 75351135-75351420(score=817) Regulation SRX3110725 level 1 Breast H.sapiens
ESR1 hsa-mir-326 chr11: 75351831(NM_020251) chr11: 75351139-75351405(score=584) Regulation SRX1012631 level 1 Breast H.sapiens
ESR1 hsa-mir-326 chr11: 75351831(NM_020251) chr11: 75351142-75351390(score=1000) Regulation SRX2513521 level 1 Breast H.sapiens
ESR1 hsa-mir-326 chr11: 75351831(NM_020251) chr11: 75351143-75351404(score=1000) Regulation SRX2513519 level 1 Breast H.sapiens
ESR1 hsa-mir-326 chr11: 75351831(NM_020251) chr11: 75351144-75351391(score=1000) Regulation SRX2513518 level 1 Breast H.sapiens
ESR1 hsa-mir-326 chr11: 75351831(NM_020251) chr11: 75351145-75351413(score=1000) Regulation SRX330067 level 1 Breast H.sapiens
ESR1 hsa-mir-326 chr11: 75351831(NM_020251) chr11: 75351149-75351416(score=1000) Regulation SRX2513517 level 1 Breast H.sapiens
ESR1 hsa-mir-326 chr11: 75351831(NM_020251) chr11: 75351150-75351478(score=1000) Regulation SRX747801 level 1 Breast H.sapiens
ESR1 hsa-mir-326 chr11: 75351831(NM_020251) chr11: 75351151-75351409(score=566) Regulation SRX3110722 level 1 Breast H.sapiens

ESR1 hsa-mir-326 chr11: 75351831(NM_020251) chr11: 75351154-75351411(score=1000) Regulation SRX176861 level 1 Breast H.sapiens
ESR1 hsa-mir-326 chr11: 75351831(NM_020251) chr11: 75351155-75351431(score=1000) Regulation SRX968963 level 1 Breast H.sapiens
ESR1 hsa-mir-326 chr11: 75351831(NM_020251) chr11: 75351156-75351444(score=1000) Regulation SRX1116525 level 1 Breast H.sapiens
ESR1 hsa-mir-326 chr11: 75351831(NM_020251) chr11: 75351158-75351351(score=659) Regulation SRX969013 level 1 Breast H.sapiens
ESR1 hsa-mir-326 chr11: 75351831(NM_020251) chr11: 75351159-75351365(score=785) Regulation SRX969014 level 1 Breast H.sapiens
ESR1 hsa-mir-326 chr11: 75351831(NM_020251) chr11: 75351159-75351383(score=497) Regulation SRX1161200 level 1 Breast H.sapiens
ESR1 hsa-mir-326 chr11: 75351831(NM_020251) chr11: 75351160-75351466(score=1000) Regulation SRX1116524 level 1 Breast H.sapiens
ESR1 hsa-mir-326 chr11: 75351831(NM_020251) chr11: 75351161-75351407(score=1000) Regulation SRX176860 level 1 Breast H.sapiens
ESR1 hsa-mir-326 chr11: 75351831(NM_020251) chr11: 75351162-75351365(score=973) Regulation SRX792932 level 1 Breast H.sapiens
ESR1 hsa-mir-326 chr11: 75351831(NM_020251) chr11: 75351163-75351394(score=512) Regulation SRX1012628 level 1 Breast H.sapiens
ESR1 hsa-mir-326 chr11: 75351831(NM_020251) chr11: 75351164-75351381(score=1000) Regulation SRX968964 level 1 Breast H.sapiens
ESR1 hsa-mir-326 chr11: 75351831(NM_020251) chr11: 75351167-75351430(score=1000) Regulation SRX1531791 level 1 Breast H.sapiens
ESR1 hsa-mir-326 chr11: 75351831(NM_020251) chr11: 75351168-75351368(score=1000) Regulation SRX792933 level 1 Breast H.sapiens
ESR1 hsa-mir-326 chr11: 75351831(NM_020251) chr11: 75351168-75351385(score=618) Regulation SRX1161201 level 1 Breast H.sapiens
ESR1 hsa-mir-326 chr11: 75351831(NM_020251) chr11: 75351169-75351395(score=919) Regulation SRX3110721 level 1 Breast H.sapiens
ESR1 hsa-mir-326 chr11: 75351831(NM_020251) chr11: 75351172-75351360(score=639) Regulation SRX371473 level 1 Breast H.sapiens
ESR1 hsa-mir-326 chr11: 75351831(NM_020251) chr11: 75351172-75351362(score=1000) Regulation SRX176856 level 1 Breast H.sapiens
ESR1 hsa-mir-326 chr11: 75351831(NM_020251) chr11: 75351172-75351393(score=1000) Regulation SRX176857 level 1 Breast H.sapiens

ESR1 hsa-mir-326 chr11: 75351831(NM_020251) chr11: 75351173-75351362(score=576) Regulation SRX371478 level 1 Breast H.sapiens
ESR1 hsa-mir-326 chr11: 75351831(NM_020251) chr11: 75351174-75351367(score=803) Regulation SRX2513523 level 1 Breast H.sapiens
ESR1 hsa-mir-326 chr11: 75351831(NM_020251) chr11: 75351179-75351383(score=1000) Regulation SRX176858 level 1 Breast H.sapiens
ESR1 hsa-mir-326 chr11: 75351831(NM_020251) chr11: 75351180-75351399(score=1000) Regulation SRX194575 level 1 Breast H.sapiens
ESR1 hsa-mir-326 chr11: 75351831(NM_020251) chr11: 75351181-75351382(score=1000) Regulation SRX176859 level 1 Breast H.sapiens
ESR1 hsa-mir-326 chr11: 75351831(NM_020251) chr11: 75351181-75351449(score=767) Regulation SRX747782 level 1 Breast H.sapiens
ESR1 hsa-mir-326 chr11: 75351831(NM_020251) chr11: 75351182-75351399(score=735) Regulation SRX1469949 level 1 Breast H.sapiens
ESR1 hsa-mir-326 chr11: 75351831(NM_020251) chr11: 75351183-75351437(score=1000) Regulation SRX730450 level 1 Breast H.sapiens
ESR1 hsa-mir-326 chr11: 75351831(NM_020251) chr11: 75351183-75351454(score=1000) Regulation SRX323400 level 1 Breast H.sapiens
ESR1 hsa-mir-326 chr11: 75351831(NM_020251) chr11: 75351184-75351375(score=438) Regulation SRX1961161 level 1 Breast H.sapiens
ESR1 hsa-mir-326 chr11: 75351831(NM_020251) chr11: 75351186-75351391(score=907) Regulation SRX1336216 level 1 Breast H.sapiens
ESR1 hsa-mir-326 chr11: 75351831(NM_020251) chr11: 75351187-75351363(score=488) Regulation SRX330070 level 1 Breast H.sapiens
ESR1 hsa-mir-326 chr11: 75351831(NM_020251) chr11: 75351189-75351410(score=804) Regulation SRX747778 level 1 Breast H.sapiens
ESR1 hsa-mir-326 chr11: 75351831(NM_020251) chr11: 75351190-75351395(score=656) Regulation SRX747777 level 1 Breast H.sapiens
ESR1 hsa-mir-326 chr11: 75351831(NM_020251) chr11: 75351191-75351349(score=467) Regulation SRX330069 level 1 Breast H.sapiens
ESR1 hsa-mir-326 chr11: 75351831(NM_020251) chr11: 75351193-75351336(score=507) Regulation SRX371469 level 1 Breast H.sapiens
ESR1 hsa-mir-326 chr11: 75351831(NM_020251) chr11: 75351193-75351386(score=878) Regulation SRX026530 level 1 Breast H.sapiens
ESR1 hsa-mir-326 chr11: 75351831(NM_020251) chr11: 75351195-75351385(score=558) Regulation SRX469396 level 1 Breast H.sapiens

ESR1 hsa-mir-326 chr11: 75351831(NM_020251) chr11: 75351195-75351386(score=564) Regulation SRX1469951 level 1 Breast H.sapiens
ESR1 hsa-mir-326 chr11: 75351831(NM_020251) chr11: 75351197-75351368(score=481) Regulation SRX1531796 level 1 Breast H.sapiens
ESR1 hsa-mir-326 chr11: 75351831(NM_020251) chr11: 75351198-75351373(score=475) Regulation SRX730451 level 1 Breast H.sapiens
ESR1 hsa-mir-326 chr11: 75351831(NM_020251) chr11: 75351198-75351381(score=519) Regulation SRX1469950 level 1 Breast H.sapiens
ESR1 hsa-mir-326 chr11: 75351831(NM_020251) chr11: 75351199-75351383(score=483) Regulation SRX1961156 level 1 Breast H.sapiens
ESR1 hsa-mir-326 chr11: 75351831(NM_020251) chr11: 75351199-75351400(score=987) Regulation SRX747805 level 1 Breast H.sapiens
ESR1 hsa-mir-326 chr11: 75351831(NM_020251) chr11: 75351200-75351371(score=833) Regulation SRX371467 level 1 Breast H.sapiens
ESR1 hsa-mir-326 chr11: 75351831(NM_020251) chr11: 75351200-75351416(score=453) Regulation SRX3110723 level 1 Breast H.sapiens
ESR1 hsa-mir-326 chr11: 75351831(NM_020251) chr11: 75351201-75351406(score=425) Regulation SRX747781 level 1 Breast H.sapiens
ESR1 hsa-mir-326 chr11: 75351831(NM_020251) chr11: 75351202-75351339(score=454) Regulation SRX107369 level 1 Breast H.sapiens
ESR1 hsa-mir-326 chr11: 75351831(NM_020251) chr11: 75351202-75351370(score=817) Regulation SRX194569 level 1 Breast H.sapiens
ESR1 hsa-mir-326 chr11: 75351831(NM_020251) chr11: 75351203-75351335(score=449) Regulation SRX100419 level 1 Breast H.sapiens
ESR1 hsa-mir-326 chr11: 75351831(NM_020251) chr11: 75351203-75351342(score=960) Regulation SRX003939 level 1 Breast H.sapiens
ESR1 hsa-mir-326 chr11: 75351831(NM_020251) chr11: 75351210-75351356(score=657) Regulation SRX1585244 level 1 Breast H.sapiens
ESR1 hsa-mir-326 chr11: 75351831(NM_020251) chr11: 75351211-75351377(score=399) Regulation SRX968967 level 1 Breast H.sapiens
ESR1 hsa-mir-326 chr11: 75351831(NM_020251) chr11: 75351211-75351378(score=406) Regulation SRX1961155 level 1 Breast H.sapiens
ESR1 hsa-mir-326 chr11: 75351831(NM_020251) chr11: 75351213-75351350(score=604) Regulation SRX003938 level 1 Breast H.sapiens
ESR1 hsa-mir-326 chr11: 75351831(NM_020251) chr11: 75351213-75351353(score=465) Regulation SRX371479 level 1 Breast H.sapiens

ESR1 hsa-mir-326 chr11: 75351831(NM_020251) chr11: 75351214-75351353(score=537) Regulation SRX100584 level 1 Breast H.sapiens
ESR1 hsa-mir-326 chr11: 75351831(NM_020251) chr11: 75351214-75351365(score=535) Regulation SRX261274 level 1 Breast H.sapiens
ESR1 hsa-mir-326 chr11: 75351831(NM_020251) chr11: 75351214-75351365(score=538) Regulation SRX730426 level 1 Breast H.sapiens
ESR1 hsa-mir-326 chr11: 75351831(NM_020251) chr11: 75351214-75351512(score=1000) Regulation SRX323398 level 1 Breast H.sapiens
ESR1 hsa-mir-326 chr11: 75351831(NM_020251) chr11: 75351218-75351352(score=460) Regulation SRX747802 level 1 Breast H.sapiens
ESR1 hsa-mir-326 chr11: 75351831(NM_020251) chr11: 75351229-75351351(score=412) Regulation SRX673753 level 1 Breast H.sapiens
ESR1 hsa-mir-326 chr11: 75351831(NM_020251) chr11: 75351241-75351365(score=378) Regulation SRX3110738 level 1 Breast H.sapiens
FGFR1 hsa-mir-326 chr11: 75351660 chr11: 75351720-75351926(score=441) Regulation SRX2442440 level 2 Pluripotent stem cell H.sapiens
FLI1 hsa-mir-326 chr11: 75351831(NM_004041) chr11: 75351408-75351701(score=773) Regulation SRX117011 level 1 Blood H.sapiens
FLI1 hsa-mir-326 chr11: 75351831(NM_004041) chr11: 75351409-75351688(score=705) Regulation SRX117010 level 1 Blood H.sapiens
FLI1 hsa-mir-326 chr11: 75351831(NM_004041) chr11: 75351809-75352490(score=846) Regulation SRX117011 level 1 Blood H.sapiens
FLI1 hsa-mir-326 chr11: 75351831(NM_004041) chr11: 75352083-75352332(score=670) Regulation SRX117010 level 1 Blood H.sapiens
FLI1 hsa-mir-326 chr11: 75351831(NM_004041) chr11: 75352126-75352673(score=1000) Regulation SRX026640 level 1 Blood H.sapiens
FLI1 hsa-mir-326 chr11: 75351831(NM_004041) chr11: 75352353-75352550(score=339) Regulation SRX1514276 level 1 Blood H.sapiens
FLI1 hsa-mir-326 chr11: 75351831(NM_020251) chr11: 75351408-75351701(score=773) Regulation SRX117011 level 1 Blood H.sapiens
FLI1 hsa-mir-326 chr11: 75351831(NM_020251) chr11: 75351409-75351688(score=705) Regulation SRX117010 level 1 Blood H.sapiens
FLI1 hsa-mir-326 chr11: 75351831(NM_020251) chr11: 75351809-75352490(score=846) Regulation SRX117011 level 1 Blood H.sapiens
FLI1 hsa-mir-326 chr11: 75351831(NM_020251) chr11: 75352083-75352332(score=670) Regulation SRX117010 level 1 Blood H.sapiens

FLI1 hsa-mir-326 chr11: 75351831(NM_020251) chr11: 75352126-75352673(score=1000) Regulation SRX026640 level 1 Blood H.sapiens
FLI1 hsa-mir-326 chr11: 75351831(NM_020251) chr11: 75352353-75352550(score=339) Regulation SRX1514276 level 1 Blood H.sapiens
FOS hsa-mir-326 chr11: 75335186 chr11: 75337367-75337581(score=506) Regulation SRX150664 level 1 Cardiovascular H.sapiens
FOXA1 hsa-mir-326 chr11: 75351660 chr11: 75351825-75351930(score=404) Regulation SRX250093 level 2 Breast H.sapiens
FOXA1 hsa-mir-326 chr11: 75335186 chr11: 75337476-75337680(score=549) Regulation SRX250093 level 1 Breast H.sapiens
FOXA1 hsa-mir-326 chr11: 75351831(NM_004041) chr11: 75351084-75351394(score=802) Regulation SRX250093 level 1 Breast H.sapiens
FOXA1 hsa-mir-326 chr11: 75351831(NM_004041) chr11: 75351157-75351349(score=355) Regulation SRX028635 level 1 Breast H.sapiens
FOXA1 hsa-mir-326 chr11: 75351831(NM_020251) chr11: 75351084-75351394(score=802) Regulation SRX250093 level 1 Breast H.sapiens
FOXA1 hsa-mir-326 chr11: 75351831(NM_020251) chr11: 75351157-75351349(score=355) Regulation SRX028635 level 1 Breast H.sapiens
FOXO1 hsa-mir-326 chr11: 75351831(NM_004041) chr11: 75351793-75352159(score=537) Regulation SRX1735651 level 1 Blood H.sapiens
FOXO1 hsa-mir-326 chr11: 75351831(NM_020251) chr11: 75351793-75352159(score=537) Regulation SRX1735651 level 1 Blood H.sapiens
GATA1 hsa-mir-326 chr11: 75335186 chr11: 75337256-75337420(score=612) Regulation SRX386202 level 1 Blood H.sapiens
GATA1 hsa-mir-326 chr11: 75335186 chr11: 75338989-75339158(score=956) Regulation SRX386202 level 1 Blood H.sapiens
GATA1 hsa-mir-326 chr11: 75351831(NM_004041) chr11: 75351184-75351300(score=234) Regulation SRX218418 level 1 Blood H.sapiens
GATA1 hsa-mir-326 chr11: 75351831(NM_004041) chr11: 75352235-75352510(score=1000) Regulation SRX386202 level 1 Blood H.sapiens
GATA1 hsa-mir-326 chr11: 75351831(NM_020251) chr11: 75351184-75351300(score=234) Regulation SRX218418 level 1 Blood H.sapiens
GATA1 hsa-mir-326 chr11: 75351831(NM_020251) chr11: 75352235-75352510(score=1000) Regulation SRX386202 level 1 Blood H.sapiens
GATA2 hsa-mir-326 chr11: 75351831(NM_004041) chr11: 75352163-75352491(score=428) Regulation SRX2574235 level 1 Blood H.sapiens

GATA2 hsa-mir-326 chr11: 75351831(NM_020251) chr11: 75352163-75352491(score=428) Regulation SRX2574235 level 1 Blood H.sapiens
GATA2 hsa-mir-326 chr11: 75335186 chr11: 75337227-75337518(score=809) Regulation SRX150427 level 1 Cardiovascular H.sapiens
GATA2 hsa-mir-326 chr11: 75335186 chr11: 75337251-75337486(score=524) Regulation SRX070877 level 1 Cardiovascular H.sapiens
GATA2 hsa-mir-326 chr11: 75351831(NM_004041) chr11: 75352216-75352444(score=601) Regulation SRX150668 level 1 Neural H.sapiens
GATA2 hsa-mir-326 chr11: 75351831(NM_020251) chr11: 75352216-75352444(score=601) Regulation SRX150668 level 1 Neural H.sapiens
GATA3 hsa-mir-326 chr11: 75335186 chr11: 75337256-75337420(score=612) Regulation SRX160844 level 1 Blood H.sapiens
GATA3 hsa-mir-326 chr11: 75335186 chr11: 75338989-75339158(score=956) Regulation SRX160844 level 1 Blood H.sapiens
GATA3 hsa-mir-326 chr11: 75351831(NM_004041) chr11: 75352235-75352510(score=1000) Regulation SRX160844 level 1 Blood H.sapiens
GATA3 hsa-mir-326 chr11: 75351831(NM_020251) chr11: 75352235-75352510(score=1000) Regulation SRX160844 level 1 Blood H.sapiens
GATA3 hsa-mir-326 chr11: 75335186 chr11: 75334633-75334751(score=317) Regulation SRX100559 level 1 Breast H.sapiens
GATA3 hsa-mir-326 chr11: 75335186 chr11: 75337266-75337454(score=783) Regulation SRX190194 level 1 Neural H.sapiens
GATA4 hsa-mir-326 chr11: 75351831(NM_004041) chr11: 75351852-75352499(score=1000) Regulation SRX367641 level 1 Digestive tract H.sapiens
GATA4 hsa-mir-326 chr11: 75351831(NM_020251) chr11: 75351852-75352499(score=1000) Regulation SRX367641 level 1 Digestive tract H.sapiens
GATA6 hsa-mir-326 chr11: 75351831(NM_004041) chr11: 75352171-75352524(score=1000) Regulation SRX288274 level 1 Pancreas H.sapiens
GATA6 hsa-mir-326 chr11: 75351831(NM_020251) chr11: 75352171-75352524(score=1000) Regulation SRX288274 level 1 Pancreas H.sapiens
GRHL2 hsa-mir-326 chr11: 75335186 chr11: 75334573-75334818(score=819) Regulation SRX1787658 level 1 Breast H.sapiens
GRHL2 hsa-mir-326 chr11: 75335186 chr11: 75334576-75334835(score=790) Regulation SRX1787657 level 1 Breast H.sapiens
GRHL2 hsa-mir-326 chr11: 75335186 chr11: 75334580-75334851(score=1000) Regulation SRX1787656 level 1 Breast H.sapiens

GRHL2 hsa-mir-326 chr11: 75351831(NM_004041) chr11: 75353152-75353473(score=1000) Regulation SRX1787657 level 1 Breast H.sapiens
GRHL2 hsa-mir-326 chr11: 75351831(NM_004041) chr11: 75353161-75353425(score=1000) Regulation SRX1787658 level 1 Breast H.sapiens
GRHL2 hsa-mir-326 chr11: 75351831(NM_004041) chr11: 75353172-75353436(score=1000) Regulation SRX1787656 level 1 Breast H.sapiens
GRHL2 hsa-mir-326 chr11: 75351831(NM_020251) chr11: 75353152-75353473(score=1000) Regulation SRX1787657 level 1 Breast H.sapiens
GRHL2 hsa-mir-326 chr11: 75351831(NM_020251) chr11: 75353161-75353425(score=1000) Regulation SRX1787658 level 1 Breast H.sapiens
GRHL2 hsa-mir-326 chr11: 75351831(NM_020251) chr11: 75353172-75353436(score=1000) Regulation SRX1787656 level 1 Breast H.sapiens
HAND2 hsa-mir-326 chr11: 75335186 chr11: 75338793-75339005(score=357) Regulation SRX2911578 level 1 Neural H.sapiens
HDAC2 hsa-mir-326 chr11: 75351831(NM_004041) chr11: 75352292-75352425(score=449) Regulation SRX100538 level 1 Liver H.sapiens
HDAC2 hsa-mir-326 chr11: 75351831(NM_020251) chr11: 75352292-75352425(score=449) Regulation SRX100538 level 1 Liver H.sapiens
HEY1 hsa-mir-326 chr11: 75351831(NM_004041) chr11: 75352186-75352307(score=345) Regulation SRX100493 level 1 Liver H.sapiens
HEY1 hsa-mir-326 chr11: 75351831(NM_020251) chr11: 75352186-75352307(score=345) Regulation SRX100493 level 1 Liver H.sapiens
HIF1A hsa-mir-326 chr11: 75351660 chr11: 75351568-75351693(score=399) Regulation SRX666556 level 2 Breast H.sapiens
HIF1A hsa-mir-326 chr11: 75351831(NM_004041) chr11: 75350980-75351394(score=988) Regulation SRX666556 level 1 Breast H.sapiens
HIF1A hsa-mir-326 chr11: 75351831(NM_004041) chr11: 75351754-75352201(score=1000) Regulation SRX666556 level 1 Breast H.sapiens
HIF1A hsa-mir-326 chr11: 75351831(NM_020251) chr11: 75350980-75351394(score=988) Regulation SRX666556 level 1 Breast H.sapiens
HIF1A hsa-mir-326 chr11: 75351831(NM_020251) chr11: 75351754-75352201(score=1000) Regulation SRX666556 level 1 Breast H.sapiens
JUN hsa-mir-326 chr11: 75335186 chr11: 75337355-75337582(score=724) Regulation SRX2355072 level 1 Cardiovascular H.sapiens
KDM2B hsa-mir-326 chr11: 75351831(NM_004041) chr11: 75351501-75352259(score=522) Regulation SRX1873447 level 1 Blood H.sapiens

KDM2B hsa-mir-326 chr11: 75351831(NM_020251) chr11: 75351501-75352259(score=522) Regulation SRX1873447 level 1 Blood H.sapiens
KDM5B hsa-mir-326 chr11: 75351660 chr11: 75351832-75351944(score=266) Regulation SRX116438 level 2 Blood H.sapiens
KDM5B hsa-mir-326 chr11: 75351660 chr11: 75351815-75351950(score=432) Regulation SRX265414 level 2 Breast H.sapiens
KDM5B hsa-mir-326 chr11: 75351831(NM_004041) chr11: 75351193-75351394(score=410) Regulation SRX265414 level 1 Breast H.sapiens
KDM5B hsa-mir-326 chr11: 75351831(NM_004041) chr11: 75351215-75351388(score=310) Regulation SRX265420 level 1 Breast H.sapiens
KDM5B hsa-mir-326 chr11: 75351831(NM_004041) chr11: 75351515-75351663(score=354) Regulation SRX265413 level 1 Breast H.sapiens
KDM5B hsa-mir-326 chr11: 75351831(NM_004041) chr11: 75351525-75351692(score=472) Regulation SRX265430 level 1 Breast H.sapiens
KDM5B hsa-mir-326 chr11: 75351831(NM_004041) chr11: 75351525-75351734(score=1000) Regulation SRX265420 level 1 Breast H.sapiens
KDM5B hsa-mir-326 chr11: 75351831(NM_004041) chr11: 75351801-75351983(score=589) Regulation SRX265423 level 1 Breast H.sapiens
KDM5B hsa-mir-326 chr11: 75351831(NM_004041) chr11: 75351803-75351993(score=620) Regulation SRX265420 level 1 Breast H.sapiens
KDM5B hsa-mir-326 chr11: 75351831(NM_004041) chr11: 75351826-75351980(score=555) Regulation SRX265413 level 1 Breast H.sapiens
KDM5B hsa-mir-326 chr11: 75351831(NM_004041) chr11: 75351828-75351971(score=530) Regulation SRX265412 level 1 Breast H.sapiens
KDM5B hsa-mir-326 chr11: 75351831(NM_020251) chr11: 75351193-75351394(score=410) Regulation SRX265414 level 1 Breast H.sapiens
KDM5B hsa-mir-326 chr11: 75351831(NM_020251) chr11: 75351215-75351388(score=310) Regulation SRX265420 level 1 Breast H.sapiens
KDM5B hsa-mir-326 chr11: 75351831(NM_020251) chr11: 75351515-75351663(score=354) Regulation SRX265413 level 1 Breast H.sapiens
KDM5B hsa-mir-326 chr11: 75351831(NM_020251) chr11: 75351525-75351692(score=472) Regulation SRX265430 level 1 Breast H.sapiens
KDM5B hsa-mir-326 chr11: 75351831(NM_020251) chr11: 75351525-75351734(score=1000) Regulation SRX265420 level 1 Breast H.sapiens
KDM5B hsa-mir-326 chr11: 75351831(NM_020251) chr11: 75351801-75351983(score=589) Regulation SRX265423 level 1 Breast H.sapiens

KDM5B hsa-mir-326 chr11: 75351831(NM_020251) chr11: 75351803-75351993(score=620) Regulation SRX265420 level 1 Breast H.sapiens
KDM5B hsa-mir-326 chr11: 75351831(NM_020251) chr11: 75351826-75351980(score=555) Regulation SRX265413 level 1 Breast H.sapiens
KDM5B hsa-mir-326 chr11: 75351831(NM_020251) chr11: 75351828-75351971(score=530) Regulation SRX265412 level 1 Breast H.sapiens
KLF1 hsa-mir-326 chr11: 75351660 chr11: 75351805-75351882(score=633) Regulation SRX218419 level 2 Blood H.sapiens
KLF1 hsa-mir-326 chr11: 75351831(NM_004041) chr11: 75351065-75351301(score=573) Regulation SRX218419 level 1 Blood H.sapiens
KLF1 hsa-mir-326 chr11: 75351831(NM_020251) chr11: 75351065-75351301(score=573) Regulation SRX218419 level 1 Blood H.sapiens
LARP7 hsa-mir-326 chr11: 75351831(NM_004041) chr11: 75351030-75351206(score=279) Regulation SRX2198831 level 1 Uterus H.sapiens
LARP7 hsa-mir-326 chr11: 75351831(NM_004041) chr11: 75351518-75351687(score=713) Regulation SRX2198835 level 1 Uterus H.sapiens
LARP7 hsa-mir-326 chr11: 75351831(NM_004041) chr11: 75351791-75352038(score=1000) Regulation SRX2198835 level 1 Uterus H.sapiens
LARP7 hsa-mir-326 chr11: 75351831(NM_020251) chr11: 75351030-75351206(score=279) Regulation SRX2198831 level 1 Uterus H.sapiens
LARP7 hsa-mir-326 chr11: 75351831(NM_020251) chr11: 75351518-75351687(score=713) Regulation SRX2198835 level 1 Uterus H.sapiens
LARP7 hsa-mir-326 chr11: 75351831(NM_020251) chr11: 75351791-75352038(score=1000) Regulation SRX2198835 level 1 Uterus H.sapiens
MAFF hsa-mir-326 chr11: 75335186 chr11: 75337265-75337444(score=955) Regulation SRX150599 level 1 Blood H.sapiens
MAFK hsa-mir-326 chr11: 75335186 chr11: 75337276-75337436(score=600) Regulation SRX150391 level 1 Blood H.sapiens
MAFK hsa-mir-326 chr11: 75335186 chr11: 75338642-75338819(score=633) Regulation SRX150483 level 1 Lung H.sapiens
MAX hsa-mir-326 chr11: 75351831(NM_004041) chr11: 75351720-75351974(score=364) Regulation SRX129084 level 1 Blood H.sapiens
MAX hsa-mir-326 chr11: 75351831(NM_004041) chr11: 75351803-75352053(score=507) Regulation SRX100568 level 1 Blood H.sapiens
MAX hsa-mir-326 chr11: 75351831(NM_020251) chr11: 75351720-75351974(score=364) Regulation SRX129084 level 1 Blood H.sapiens

MAX hsa-mir-326 chr11: 75351831(NM_020251) chr11: 75351803-75352053(score=507) Regulation SRX100568 level 1 Blood H.sapiens
MAX hsa-mir-326 chr11: 75351831(NM_004041) chr11: 75351010-75351318(score=531) Regulation SRX190319 level 1 Breast H.sapiens
MAX hsa-mir-326 chr11: 75351831(NM_020251) chr11: 75351010-75351318(score=531) Regulation SRX190319 level 1 Breast H.sapiens
MAX hsa-mir-326 chr11: 75335186 chr11: 75338933-75339188(score=807) Regulation SRX189237 level 1 Lung H.sapiens
MAX hsa-mir-326 chr11: 75335186 chr11: 75338957-75339183(score=560) Regulation SRX129111 level 1 Lung H.sapiens
MAZ hsa-mir-326 chr11: 75351831(NM_004041) chr11: 75352276-75352472(score=661) Regulation(feedback) SRX150417 level 1 Blood H.sapiens
MAZ hsa-mir-326 chr11: 75351831(NM_020251) chr11: 75352276-75352472(score=661) Regulation(feedback) SRX150417 level 1 Blood H.sapiens
MXI1 hsa-mir-326 chr11: 75351831(NM_004041) chr11: 75351156-75351343(score=297) Regulation SRX186636 level 1 Neural H.sapiens
MXI1 hsa-mir-326 chr11: 75351831(NM_020251) chr11: 75351156-75351343(score=297) Regulation SRX186636 level 1 Neural H.sapiens
MYC hsa-mir-326 chr11: 75351831(NM_004041) chr11: 75351031-75351332(score=547) Regulation SRX103000 level 1 Breast H.sapiens
MYC hsa-mir-326 chr11: 75351831(NM_004041) chr11: 75351043-75351338(score=482) Regulation SRX099865 level 1 Breast H.sapiens
MYC hsa-mir-326 chr11: 75351831(NM_004041) chr11: 75351127-75351288(score=443) Regulation SRX103003 level 1 Breast H.sapiens
MYC hsa-mir-326 chr11: 75351831(NM_004041) chr11: 75351152-75351410(score=637) Regulation SRX250091 level 1 Breast H.sapiens
MYC hsa-mir-326 chr11: 75351831(NM_004041) chr11: 75351158-75351310(score=427) Regulation SRX188943 level 1 Breast H.sapiens
MYC hsa-mir-326 chr11: 75351831(NM_004041) chr11: 75351174-75351316(score=564) Regulation SRX188954 level 1 Breast H.sapiens
MYC hsa-mir-326 chr11: 75351831(NM_004041) chr11: 75351792-75352011(score=1000) Regulation SRX250091 level 1 Breast H.sapiens
MYC hsa-mir-326 chr11: 75351831(NM_020251) chr11: 75351031-75351332(score=547) Regulation SRX103000 level 1 Breast H.sapiens
MYC hsa-mir-326 chr11: 75351831(NM_020251) chr11: 75351043-75351338(score=482) Regulation SRX099865 level 1 Breast H.sapiens

MYC hsa-mir-326 chr11: 75351831(NM_020251) chr11: 75351127-75351288(score=443) Regulation SRX103003 level 1 Breast H.sapiens
MYC hsa-mir-326 chr11: 75351831(NM_020251) chr11: 75351152-75351410(score=637) Regulation SRX250091 level 1 Breast H.sapiens
MYC hsa-mir-326 chr11: 75351831(NM_020251) chr11: 75351158-75351310(score=427) Regulation SRX188943 level 1 Breast H.sapiens
MYC hsa-mir-326 chr11: 75351831(NM_020251) chr11: 75351174-75351316(score=564) Regulation SRX188954 level 1 Breast H.sapiens
MYC hsa-mir-326 chr11: 75351831(NM_020251) chr11: 75351792-75352011(score=1000) Regulation SRX250091 level 1 Breast H.sapiens
MYC hsa-mir-326 chr11: 75335186 chr11: 75338925-75339186(score=822) Regulation SRX129110 level 1 Lung H.sapiens
MYCN hsa-mir-326 chr11: 75335186 chr11: 75338926-75339217(score=1000) Regulation SRX1690205 level 1 Neural H.sapiens
MYCN hsa-mir-326 chr11: 75351831(NM_004041) chr11: 75351738-75352038(score=459) Regulation SRX1690205 level 1 Neural H.sapiens
MYCN hsa-mir-326 chr11: 75351831(NM_020251) chr11: 75351738-75352038(score=459) Regulation SRX1690205 level 1 Neural H.sapiens
NANOG hsa-mir-326 chr11: 75335186 chr11: 75335100-75335294(score=496) Regulation SRX2881137 level 1 Pluripotent stem cell H.sapiens
NFE2 hsa-mir-326 chr11: 75351660 chr11: 75351815-75351878(score=445) Regulation SRX218420 level 2 Blood H.sapiens
NFIC hsa-mir-326 chr11: 75351831(NM_004041) chr11: 75355125-75355328(score=572) Regulation SRX190311 level 1 Uterus H.sapiens
NFIC hsa-mir-326 chr11: 75351831(NM_020251) chr11: 75355125-75355328(score=572) Regulation SRX190311 level 1 Uterus H.sapiens
NR3C1 hsa-mir-326 chr11: 75351660 chr11: 75351820-75351940(score=367) Regulation SRX100416 level 2 Lung H.sapiens
NR3C1 hsa-mir-326 chr11: 75335186 chr11: 75338514-75338785(score=557) Regulation SRX2609664 level 1 Lung H.sapiens
NR3C1 hsa-mir-326 chr11: 75335186 chr11: 75338519-75338812(score=639) Regulation SRX2609662 level 1 Lung H.sapiens
NR3C1 hsa-mir-326 chr11: 75335186 chr11: 75338523-75338692(score=368) Regulation ERX593132 level 1 Lung H.sapiens
NR3C1 hsa-mir-326 chr11: 75335186 chr11: 75338538-75338790(score=434) Regulation SRX2609665 level 1 Lung H.sapiens

NR3C1 hsa-mir-326 chr11: 75335186 chr11: 75338571-75338780(score=415) Regulation SRX2609666 level 1 Lung H.sapiens
NRF1 hsa-mir-326 chr11: 75351660 chr11: 75351780-75351954(score=767) Regulation SRX1280452 level 2 Breast H.sapiens
NRF1 hsa-mir-326 chr11: 75351660 chr11: 75351782-75351925(score=780) Regulation SRX1280451 level 2 Breast H.sapiens
NRF1 hsa-mir-326 chr11: 75351660 chr11: 75351828-75351931(score=407) Regulation SRX1280449 level 2 Breast H.sapiens
PHF8 hsa-mir-326 chr11: 75351831(NM_004041) chr11: 75351478-75351654(score=622) Regulation(feedback) SRX186646 level 1 Blood H.sapiens
PHF8 hsa-mir-326 chr11: 75351831(NM_004041) chr11: 75351498-75351644(score=497) Regulation(feedback) SRX116437 level 1 Blood H.sapiens
PHF8 hsa-mir-326 chr11: 75351831(NM_004041) chr11: 75351503-75351639(score=385) Regulation(feedback) SRX116436 level 1 Blood H.sapiens
PHF8 hsa-mir-326 chr11: 75351831(NM_004041) chr11: 75351807-75352272(score=1000) Regulation(feedback) SRX186646 level 1 Blood H.sapiens
PHF8 hsa-mir-326 chr11: 75351831(NM_004041) chr11: 75351813-75352258(score=853) Regulation(feedback) SRX116437 level 1 Blood H.sapiens
PHF8 hsa-mir-326 chr11: 75351831(NM_004041) chr11: 75351819-75352239(score=830) Regulation(feedback) SRX116436 level 1 Blood H.sapiens
PHF8 hsa-mir-326 chr11: 75351831(NM_020251) chr11: 75351478-75351654(score=622) Regulation(feedback) SRX186646 level 1 Blood H.sapiens
PHF8 hsa-mir-326 chr11: 75351831(NM_020251) chr11: 75351498-75351644(score=497) Regulation(feedback) SRX116437 level 1 Blood H.sapiens
PHF8 hsa-mir-326 chr11: 75351831(NM_020251) chr11: 75351503-75351639(score=385) Regulation(feedback) SRX116436 level 1 Blood H.sapiens
PHF8 hsa-mir-326 chr11: 75351831(NM_020251) chr11: 75351807-75352272(score=1000) Regulation(feedback) SRX186646 level 1 Blood H.sapiens
PHF8 hsa-mir-326 chr11: 75351831(NM_020251) chr11: 75351813-75352258(score=853) Regulation(feedback) SRX116437 level 1 Blood H.sapiens
PHF8 hsa-mir-326 chr11: 75351831(NM_020251) chr11: 75351819-75352239(score=830) Regulation(feedback) SRX116436 level 1 Blood H.sapiens
PML hsa-mir-326 chr11: 75351831(NM_004041) chr11: 75352357-75352452(score=281) Regulation SRX014780 level 1 Blood H.sapiens
PML hsa-mir-326 chr11: 75351831(NM_020251) chr11: 75352357-75352452(score=281) Regulation SRX014780 level 1 Blood H.sapiens

PRDM5 hsa-mir-326 chr11: 75351831(NM_004041) chr11: 75356672-75356798(score=841) Regulation SRX018654 level 1 Muscle H.sapiens
PRDM5 hsa-mir-326 chr11: 75351831(NM_020251) chr11: 75356672-75356798(score=841) Regulation SRX018654 level 1 Muscle H.sapiens
RAD21 hsa-mir-326 chr11: 75351831(NM_004041) chr11: 75352365-75352475(score=595) Regulation SRX100492 level 1 Blood H.sapiens
RAD21 hsa-mir-326 chr11: 75351831(NM_020251) chr11: 75352365-75352475(score=595) Regulation SRX100492 level 1 Blood H.sapiens
RAD21 hsa-mir-326 chr11: 75335186 chr11: 75339770-75339927(score=604) Regulation SRX190247 level 1 Breast H.sapiens
RAD21 hsa-mir-326 chr11: 75335186 chr11: 75339787-75339930(score=403) Regulation SRX1165105 level 1 Breast H.sapiens
RAD21 hsa-mir-326 chr11: 75335186 chr11: 75339785-75339925(score=541) Regulation SRX190304 level 1 Digestive tract H.sapiens
RAD21 hsa-mir-326 chr11: 75351831(NM_004041) chr11: 75352324-75352472(score=586) Regulation SRX100562 level 1 Liver H.sapiens
RAD21 hsa-mir-326 chr11: 75351831(NM_004041) chr11: 75352347-75352476(score=432) Regulation SRX1165089 level 1 Liver H.sapiens
RAD21 hsa-mir-326 chr11: 75351831(NM_004041) chr11: 75352350-75352457(score=373) Regulation SRX1165087 level 1 Liver H.sapiens
RAD21 hsa-mir-326 chr11: 75351831(NM_020251) chr11: 75352324-75352472(score=586) Regulation SRX100562 level 1 Liver H.sapiens
RAD21 hsa-mir-326 chr11: 75351831(NM_020251) chr11: 75352347-75352476(score=432) Regulation SRX1165089 level 1 Liver H.sapiens
RAD21 hsa-mir-326 chr11: 75351831(NM_020251) chr11: 75352350-75352457(score=373) Regulation SRX1165087 level 1 Liver H.sapiens
RAD21 hsa-mir-326 chr11: 75335186 chr11: 75339780-75339923(score=635) Regulation SRX190217 level 1 Lung H.sapiens
RAD21 hsa-mir-326 chr11: 75335186 chr11: 75339793-75339909(score=483) Regulation SRX100542 level 1 Neural H.sapiens
RAD21 hsa-mir-326 chr11: 75335186 chr11: 75339770-75339942(score=917) Regulation SRX100511 level 1 Pluripotent stem cell H.sapiens
RAD21 hsa-mir-326 chr11: 75335186 chr11: 75339800-75339922(score=578) Regulation SRX150650 level 1 Uterus H.sapiens
RARA hsa-mir-326 chr11: 75351831(NM_004041) chr11: 75351857-75352276(score=421) Regulation SRX682379 level 1 Blood H.sapiens

RARA hsa-mir-326 chr11: 75351831(NM_004041) chr11: 75352314-75352550(score=676) Regulation SRX014782 level 1 Blood H.sapiens
RARA hsa-mir-326 chr11: 75351831(NM_020251) chr11: 75351857-75352276(score=421) Regulation SRX682379 level 1 Blood H.sapiens
RARA hsa-mir-326 chr11: 75351831(NM_020251) chr11: 75352314-75352550(score=676) Regulation SRX014782 level 1 Blood H.sapiens
RELA hsa-mir-326 chr11: 75351831(NM_004041) chr11: 75351182-75351388(score=546) Regulation SRX968971 level 1 Breast H.sapiens
RELA hsa-mir-326 chr11: 75351831(NM_020251) chr11: 75351182-75351388(score=546) Regulation SRX968971 level 1 Breast H.sapiens
RELA hsa-mir-326 chr11: 75351660 chr11: 75351590-75351723(score=350) Regulation SRX2355085 level 2 Cardiovascular H.sapiens
RELA hsa-mir-326 chr11: 75335186 chr11: 75337397-75337537(score=483) Regulation SRX2355085 level 1 Cardiovascular H.sapiens
REST hsa-mir-326 chr11: 75351831(NM_004041) chr11: 75352434-75352625(score=888) Regulation SRX190210 level 1 Blood H.sapiens
REST hsa-mir-326 chr11: 75351831(NM_004041) chr11: 75352484-75352588(score=445) Regulation SRX475784 level 1 Blood H.sapiens
REST hsa-mir-326 chr11: 75351831(NM_004041) chr11: 75352487-75352568(score=473) Regulation SRX475777 level 1 Blood H.sapiens
REST hsa-mir-326 chr11: 75351831(NM_004041) chr11: 75352488-75352555(score=367) Regulation SRX392781 level 1 Blood H.sapiens
REST hsa-mir-326 chr11: 75351831(NM_004041) chr11: 75352488-75352589(score=473) Regulation SRX475782 level 1 Blood H.sapiens
REST hsa-mir-326 chr11: 75351831(NM_020251) chr11: 75352434-75352625(score=888) Regulation SRX190210 level 1 Blood H.sapiens
REST hsa-mir-326 chr11: 75351831(NM_020251) chr11: 75352484-75352588(score=445) Regulation SRX475784 level 1 Blood H.sapiens
REST hsa-mir-326 chr11: 75351831(NM_020251) chr11: 75352487-75352568(score=473) Regulation SRX475777 level 1 Blood H.sapiens
REST hsa-mir-326 chr11: 75351831(NM_020251) chr11: 75352488-75352555(score=367) Regulation SRX392781 level 1 Blood H.sapiens
REST hsa-mir-326 chr11: 75351831(NM_020251) chr11: 75352488-75352589(score=473) Regulation SRX475782 level 1 Blood H.sapiens
REST hsa-mir-326 chr11: 75351831(NM_004041) chr11: 75352484-75352583(score=438) Regulation SRX100410 level 1 Pluripotent stem cell H.sapiens

REST hsa-mir-326 chr11: 75351831(NM_020251) chr11: 75352484-75352583(score=438) Regulation SRX100410 level 1 Pluripotent stem cell H.sapiens
REST hsa-mir-326 chr11: 75351831(NM_004041) chr11: 75352464-75352608(score=540) Regulation SRX190297 level 1 Uterus H.sapiens
REST hsa-mir-326 chr11: 75351831(NM_020251) chr11: 75352464-75352608(score=540) Regulation SRX190297 level 1 Uterus H.sapiens
RUNX1 hsa-mir-326 chr11: 75351831(NM_004041) chr11: 75351468-75352026(score=1000) Regulation SRX265222 level 1 Blood H.sapiens
RUNX1 hsa-mir-326 chr11: 75351831(NM_004041) chr11: 75351511-75351978(score=675) Regulation SRX063913 level 1 Blood H.sapiens
RUNX1 hsa-mir-326 chr11: 75351831(NM_004041) chr11: 75352271-75352507(score=606) Regulation SRX682384 level 1 Blood H.sapiens
RUNX1 hsa-mir-326 chr11: 75351831(NM_020251) chr11: 75351468-75352026(score=1000) Regulation SRX265222 level 1 Blood H.sapiens
RUNX1 hsa-mir-326 chr11: 75351831(NM_020251) chr11: 75351511-75351978(score=675) Regulation SRX063913 level 1 Blood H.sapiens
RUNX1 hsa-mir-326 chr11: 75351831(NM_020251) chr11: 75352271-75352507(score=606) Regulation SRX682384 level 1 Blood H.sapiens
RUNX1T1 hsa-mir-326 chr11: 75351831(NM_004041) chr11: 75352316-75352487(score=533) Regulation SRX747572 level 1 Blood H.sapiens
RUNX1T1 hsa-mir-326 chr11: 75351831(NM_020251) chr11: 75352316-75352487(score=533) Regulation SRX747572 level 1 Blood H.sapiens
SMAD1 hsa-mir-326 chr11: 75335186 chr11: 75334589-75334688(score=363) Regulation SRX097106 level 1 Blood H.sapiens
SMC1A hsa-mir-326 chr11: 75351831(NM_004041) chr11: 75352330-75352488(score=352) Regulation SRX2770858 level 1 Blood H.sapiens
SMC1A hsa-mir-326 chr11: 75351831(NM_020251) chr11: 75352330-75352488(score=352) Regulation SRX2770858 level 1 Blood H.sapiens
SMC1A hsa-mir-326 chr11: 75351660 chr11: 75351561-75351910(score=553) Regulation SRX1013334 level 2 Liver H.sapiens
SMC1A hsa-mir-326 chr11: 75351831(NM_004041) chr11: 75352344-75352474(score=412) Regulation SRX1013333 level 1 Liver H.sapiens
SMC1A hsa-mir-326 chr11: 75351831(NM_004041) chr11: 75352352-75352510(score=404) Regulation SRX1013334 level 1 Liver H.sapiens
SMC1A hsa-mir-326 chr11: 75351831(NM_020251) chr11: 75352344-75352474(score=412) Regulation SRX1013333 level 1 Liver H.sapiens

SMC1A hsa-mir-326 chr11: 75351831(NM_020251) chr11: 75352352-75352510(score=404) Regulation SRX1013334 level 1 Liver H.sapiens
SPI1 hsa-mir-326 chr11: 75335186 chr11: 75337288-75337629(score=1000) Regulation SRX2770855 level 1 Blood H.sapiens
SPI1 hsa-mir-326 chr11: 75335186 chr11: 75337300-75337618(score=1000) Regulation SRX2770857 level 1 Blood H.sapiens
SPI1 hsa-mir-326 chr11: 75335186 chr11: 75337305-75337610(score=1000) Regulation SRX2770854 level 1 Blood H.sapiens
SPI1 hsa-mir-326 chr11: 75335186 chr11: 75337335-75337606(score=1000) Regulation SRX1023792 level 1 Blood H.sapiens
SPI1 hsa-mir-326 chr11: 75335186 chr11: 75337355-75337596(score=993) Regulation SRX1023793 level 1 Blood H.sapiens
SPI1 hsa-mir-326 chr11: 75335186 chr11: 75337359-75337564(score=687) Regulation SRX1023790 level 1 Blood H.sapiens
SPI1 hsa-mir-326 chr11: 75335186 chr11: 75337376-75337543(score=675) Regulation SRX1023791 level 1 Blood H.sapiens
SPI1 hsa-mir-326 chr11: 75335186 chr11: 75337386-75337529(score=416) Regulation SRX3244991 level 1 Blood H.sapiens
SPI1 hsa-mir-326 chr11: 75335186 chr11: 75337395-75337527(score=356) Regulation SRX2770856 level 1 Blood H.sapiens
SPI1 hsa-mir-326 chr11: 75351831(NM_004041) chr11: 75352269-75352543(score=822) Regulation SRX1089833 level 1 Blood H.sapiens
SPI1 hsa-mir-326 chr11: 75351831(NM_004041) chr11: 75352286-75352555(score=1000) Regulation SRX1023792 level 1 Blood H.sapiens
SPI1 hsa-mir-326 chr11: 75351831(NM_004041) chr11: 75352289-75352543(score=1000) Regulation SRX2770855 level 1 Blood H.sapiens
SPI1 hsa-mir-326 chr11: 75351831(NM_004041) chr11: 75352299-75352532(score=735) Regulation SRX794057 level 1 Blood H.sapiens
SPI1 hsa-mir-326 chr11: 75351831(NM_004041) chr11: 75352302-75352534(score=753) Regulation SRX1023790 level 1 Blood H.sapiens
SPI1 hsa-mir-326 chr11: 75351831(NM_004041) chr11: 75352311-75352491(score=458) Regulation SRX627430 level 1 Blood H.sapiens
SPI1 hsa-mir-326 chr11: 75351831(NM_004041) chr11: 75352332-75352514(score=560) Regulation SRX1023793 level 1 Blood H.sapiens
SPI1 hsa-mir-326 chr11: 75351831(NM_004041) chr11: 75352334-75352454(score=245) Regulation SRX100429 level 1 Blood H.sapiens

SPI1 hsa-mir-326 chr11: 75351831(NM_004041) chr11: 75352338-75352486(score=475) Regulation SRX2770857 level 1 Blood H.sapiens
SPI1 hsa-mir-326 chr11: 75351831(NM_004041) chr11: 75352338-75352521(score=479) Regulation SRX2268286 level 1 Blood H.sapiens
SPI1 hsa-mir-326 chr11: 75351831(NM_004041) chr11: 75352340-75352511(score=540) Regulation SRX2268287 level 1 Blood H.sapiens
SPI1 hsa-mir-326 chr11: 75351831(NM_004041) chr11: 75352383-75352507(score=408) Regulation SRX190299 level 1 Blood H.sapiens
SPI1 hsa-mir-326 chr11: 75351831(NM_004041) chr11: 75352384-75352489(score=303) Regulation SRX1431740 level 1 Blood H.sapiens
SPI1 hsa-mir-326 chr11: 75351831(NM_020251) chr11: 75352269-75352543(score=822) Regulation SRX1089833 level 1 Blood H.sapiens
SPI1 hsa-mir-326 chr11: 75351831(NM_020251) chr11: 75352286-75352555(score=1000) Regulation SRX1023792 level 1 Blood H.sapiens
SPI1 hsa-mir-326 chr11: 75351831(NM_020251) chr11: 75352289-75352543(score=1000) Regulation SRX2770855 level 1 Blood H.sapiens
SPI1 hsa-mir-326 chr11: 75351831(NM_020251) chr11: 75352299-75352532(score=735) Regulation SRX794057 level 1 Blood H.sapiens
SPI1 hsa-mir-326 chr11: 75351831(NM_020251) chr11: 75352302-75352534(score=753) Regulation SRX1023790 level 1 Blood H.sapiens
SPI1 hsa-mir-326 chr11: 75351831(NM_020251) chr11: 75352311-75352491(score=458) Regulation SRX627430 level 1 Blood H.sapiens
SPI1 hsa-mir-326 chr11: 75351831(NM_020251) chr11: 75352332-75352514(score=560) Regulation SRX1023793 level 1 Blood H.sapiens
SPI1 hsa-mir-326 chr11: 75351831(NM_020251) chr11: 75352334-75352454(score=245) Regulation SRX100429 level 1 Blood H.sapiens
SPI1 hsa-mir-326 chr11: 75351831(NM_020251) chr11: 75352338-75352486(score=475) Regulation SRX2770857 level 1 Blood H.sapiens
SPI1 hsa-mir-326 chr11: 75351831(NM_020251) chr11: 75352338-75352521(score=479) Regulation SRX2268286 level 1 Blood H.sapiens
SPI1 hsa-mir-326 chr11: 75351831(NM_020251) chr11: 75352340-75352511(score=540) Regulation SRX2268287 level 1 Blood H.sapiens
SPI1 hsa-mir-326 chr11: 75351831(NM_020251) chr11: 75352383-75352507(score=408) Regulation SRX190299 level 1 Blood H.sapiens
SPI1 hsa-mir-326 chr11: 75351831(NM_020251) chr11: 75352384-75352489(score=303) Regulation SRX1431740 level 1 Blood H.sapiens

STAG1 hsa-mir-326 chr11: 75351831(NM_004041) chr11: 75352286-75352525(score=795) Regulation SRX995501 level 1 Blood H.sapiens
STAG1 hsa-mir-326 chr11: 75351831(NM_020251) chr11: 75352286-75352525(score=795) Regulation SRX995501 level 1 Blood H.sapiens
STAT1 hsa-mir-326 chr11: 75335186 chr11: 75337371-75337631(score=851) Regulation SRX212650 level 1 Blood H.sapiens
STAT1 hsa-mir-326 chr11: 75335186 chr11: 75337381-75337596(score=495) Regulation SRX212649 level 1 Blood H.sapiens
STAT1 hsa-mir-326 chr11: 75335186 chr11: 75337408-75337618(score=626) Regulation SRX212648 level 1 Blood H.sapiens
STAT1 hsa-mir-326 chr11: 75351831(NM_004041) chr11: 75351817-75351980(score=488) Regulation SRX212648 level 1 Blood H.sapiens
STAT1 hsa-mir-326 chr11: 75351831(NM_004041) chr11: 75351830-75352031(score=424) Regulation SRX212650 level 1 Blood H.sapiens
STAT1 hsa-mir-326 chr11: 75351831(NM_004041) chr11: 75352324-75352530(score=504) Regulation SRX212648 level 1 Blood H.sapiens
STAT1 hsa-mir-326 chr11: 75351831(NM_004041) chr11: 75352331-75352520(score=554) Regulation SRX212650 level 1 Blood H.sapiens
STAT1 hsa-mir-326 chr11: 75351831(NM_020251) chr11: 75351817-75351980(score=488) Regulation SRX212648 level 1 Blood H.sapiens
STAT1 hsa-mir-326 chr11: 75351831(NM_020251) chr11: 75351830-75352031(score=424) Regulation SRX212650 level 1 Blood H.sapiens
STAT1 hsa-mir-326 chr11: 75351831(NM_020251) chr11: 75352324-75352530(score=504) Regulation SRX212648 level 1 Blood H.sapiens
STAT1 hsa-mir-326 chr11: 75351831(NM_020251) chr11: 75352331-75352520(score=554) Regulation SRX212650 level 1 Blood H.sapiens
STAT3 hsa-mir-326 chr11: 75335186 chr11: 75334551-75334719(score=499) Regulation SRX2020842 level 1 Breast H.sapiens
STAT3 hsa-mir-326 chr11: 75335186 chr11: 75334568-75334726(score=425) Regulation SRX2020843 level 1 Breast H.sapiens
SUMO2 hsa-mir-326 chr11: 75351660 chr11: 75351747-75351920(score=391) Regulation SRX769726 level 2 Prostate H.sapiens
SUMO2 hsa-mir-326 chr11: 75351831(NM_004041) chr11: 75351804-75351961(score=264) Regulation SRX1079388 level 1 Prostate H.sapiens
SUMO2 hsa-mir-326 chr11: 75351831(NM_020251) chr11: 75351804-75351961(score=264) Regulation SRX1079388 level 1 Prostate H.sapiens

TAL1 hsa-mir-326 chr11: 75335186 chr11: 75334578-75334734(score=870) Regulation SRX150575 level 1 Blood H.sapiens
TAL1 hsa-mir-326 chr11: 75351831(NM_004041) chr11: 75351483-75351697(score=607) Regulation SRX265227 level 1 Blood H.sapiens
TAL1 hsa-mir-326 chr11: 75351831(NM_020251) chr11: 75351483-75351697(score=607) Regulation SRX265227 level 1 Blood H.sapiens
TCF12 hsa-mir-326 chr11: 75351831(NM_004041) chr11: 75351466-75352005(score=879) Regulation SRX265225 level 1 Blood H.sapiens
TCF12 hsa-mir-326 chr11: 75351831(NM_020251) chr11: 75351466-75352005(score=879) Regulation SRX265225 level 1 Blood H.sapiens
TEAD4 hsa-mir-326 chr11: 75335186 chr11: 75336969-75337156(score=492) Regulation SRX1948680 level 1 Neural H.sapiens
TEAD4 hsa-mir-326 chr11: 75335186 chr11: 75338735-75339045(score=904) Regulation SRX1948680 level 1 Neural H.sapiens
TFAP2C hsa-mir-326 chr11: 75335186 chr11: 75335045-75335163(score=403) Regulation SRX128102 level 1 Breast H.sapiens
TFAP2C hsa-mir-326 chr11: 75335186 chr11: 75337495-75337661(score=566) Regulation SRX128100 level 1 Breast H.sapiens
TFAP2C hsa-mir-326 chr11: 75335186 chr11: 75337512-75337635(score=435) Regulation SRX128101 level 1 Breast H.sapiens
TFAP4 hsa-mir-326 chr11: 75351660 chr11: 75351690-75351843(score=374) Regulation SRX1892834 level 2 Digestive tract H.sapiens
TP53 hsa-mir-326 chr11: 75351831(NM_004041) chr11: 75351181-75351311(score=617) Regulation ERX181467 level 1 Bone H.sapiens
TP53 hsa-mir-326 chr11: 75351831(NM_004041) chr11: 75356618-75356779(score=399) Regulation SRX2060925 level 1 Bone H.sapiens
TP53 hsa-mir-326 chr11: 75351831(NM_004041) chr11: 75356619-75356791(score=399) Regulation SRX016980 level 1 Bone H.sapiens
TP53 hsa-mir-326 chr11: 75351831(NM_004041) chr11: 75356657-75356744(score=335) Regulation ERX181467 level 1 Bone H.sapiens
TP53 hsa-mir-326 chr11: 75351831(NM_020251) chr11: 75351181-75351311(score=617) Regulation ERX181467 level 1 Bone H.sapiens
TP53 hsa-mir-326 chr11: 75351831(NM_020251) chr11: 75356618-75356779(score=399) Regulation SRX2060925 level 1 Bone H.sapiens
TP53 hsa-mir-326 chr11: 75351831(NM_020251) chr11: 75356619-75356791(score=399) Regulation SRX016980 level 1 Bone H.sapiens

TP53 hsa-mir-326 chr11: 75351831(NM_020251) chr11: 75356657-75356744(score=335) Regulation ERX181467 level 1 Bone H.sapiens
TP63 hsa-mir-326 chr11: 75351831(NM_004041) chr11: 75356577-75356799(score=707) Regulation SRX663252 level 1 Epidermis H.sapiens
TP63 hsa-mir-326 chr11: 75351831(NM_004041) chr11: 75356584-75356804(score=772) Regulation SRX663251 level 1 Epidermis H.sapiens
TP63 hsa-mir-326 chr11: 75351831(NM_004041) chr11: 75356590-75356780(score=479) Regulation SRX663250 level 1 Epidermis H.sapiens
TP63 hsa-mir-326 chr11: 75351831(NM_020251) chr11: 75356577-75356799(score=707) Regulation SRX663252 level 1 Epidermis H.sapiens
TP63 hsa-mir-326 chr11: 75351831(NM_020251) chr11: 75356584-75356804(score=772) Regulation SRX663251 level 1 Epidermis H.sapiens
TP63 hsa-mir-326 chr11: 75351831(NM_020251) chr11: 75356590-75356780(score=479) Regulation SRX663250 level 1 Epidermis H.sapiens
TRIM24 hsa-mir-326 chr11: 75351831(NM_004041) chr11: 75351512-75351978(score=739) Regulation SRX1041842 level 1 Prostate H.sapiens
TRIM24 hsa-mir-326 chr11: 75351831(NM_020251) chr11: 75351512-75351978(score=739) Regulation SRX1041842 level 1 Prostate H.sapiens
TRIM25 hsa-mir-326 chr11: 75351831(NM_004041) chr11: 75351365-75351603(score=289) Regulation SRX1660873 level 1 Breast H.sapiens
TRIM25 hsa-mir-326 chr11: 75351831(NM_020251) chr11: 75351365-75351603(score=289) Regulation SRX1660873 level 1 Breast H.sapiens
TRIM28 hsa-mir-326 chr11: 75351831(NM_004041) chr11: 75351824-75352086(score=405) Regulation SRX391683 level 1 Blood H.sapiens
TRIM28 hsa-mir-326 chr11: 75351831(NM_004041) chr11: 75352306-75352537(score=646) Regulation SRX391683 level 1 Blood H.sapiens
TRIM28 hsa-mir-326 chr11: 75351831(NM_020251) chr11: 75351824-75352086(score=405) Regulation SRX391683 level 1 Blood H.sapiens
TRIM28 hsa-mir-326 chr11: 75351831(NM_020251) chr11: 75352306-75352537(score=646) Regulation SRX391683 level 1 Blood H.sapiens
WDR5 hsa-mir-326 chr11: 75351660 chr11: 75351568-75351906(score=312) Regulation SRX689366 level 2 Kidney H.sapiens
XBP1 hsa-mir-326 chr11: 75335186 chr11: 75334661-75334769(score=583) Regulation(feedback) SRX384490 level 1 Breast H.sapiens
XBP1 hsa-mir-326 chr11: 75335186 chr11: 75334665-75334763(score=488) Regulation(feedback) SRX389294 level 1 Breast H.sapiens

ZBTB7A hsa-mir-326 chr11: 75351831(NM_004041) chr11: 75351510-75351621(score=530) Regulation(feedback) SRX100518 level 1 Blood H.sapiens
ZBTB7A hsa-mir-326 chr11: 75351831(NM_004041) chr11: 75352195-75352476(score=938) Regulation(feedback) SRX100518 level 1 Blood H.sapiens
ZBTB7A hsa-mir-326 chr11: 75351831(NM_020251) chr11: 75351510-75351621(score=530) Regulation(feedback) SRX100518 level 1 Blood H.sapiens
ZBTB7A hsa-mir-326 chr11: 75351831(NM_020251) chr11: 75352195-75352476(score=938) Regulation(feedback) SRX100518 level 1 Blood H.sapiens
ZBTB7A hsa-mir-326 chr11: 75351831(NM_004041) chr11: 75352302-75352454(score=505) Regulation(feedback) SRX190287 level 1 Liver H.sapiens
ZBTB7A hsa-mir-326 chr11: 75351831(NM_020251) chr11: 75352302-75352454(score=505) Regulation(feedback) SRX190287 level 1 Liver H.sapiens
ZMYND8 hsa-mir-326 chr11: 75335186 chr11: 75337418-75337683(score=498) Regulation SRX1521297 level 1 Breast H.sapiens
ZNF384 hsa-mir-326 chr11: 75335186 chr11: 75337309-75337491(score=661) Regulation SRX186626 level 1 Blood H.sapiens

Chr-chromosome; SRAID- ChIP-seq derived regulations; PMID-Reference in PubMed or literature-curated regulations; Level 1-predicted interactions; Level 2- promoter- supported by high-throughput experimental data; Tissue-The tissue from which the ChIP-seq data was derived [21].
